# Intrinsic annealing in a hybrid memristor-magnetic tunnel junction Ising machine

**DOI:** 10.1038/s41467-026-71844-8

**Published:** 2026-04-16

**Authors:** Mohammed Akib Iftakher, Hugo Levices, Kamel-Eddine Harabi, Adrien Renaudineau, Mathieu-Coumba Faye, Corentin Bouchard, Florian Disdier, Bernard Viala, Elisa Vianello, Philippe Talatchian, Kevin Garello, Damien Querlioz, Louis Hutin

**Affiliations:** 1 Université Paris-Saclay, CNRS, Centre de Nanosciences et de Nanotechnologies, Palaiseau, France; 2 Université Grenoble-Alpes, CEA, LETI, Grenoble, France; 3 Université Grenoble-Alpes, CEA, CNRS, Grenoble INP, SPINTEC, Grenoble, France

**Keywords:** Electronic devices, Magnetic devices, Electronic and spintronic devices, Electrical and electronic engineering

## Abstract

Hardware implementations of the Ising model offer promising solutions to large-scale optimization tasks. In the literature, various nanodevices have been shown to emulate the spin dynamics for such Ising machines with remarkable effectiveness. Other nanodevices have been shown to implement spin-spin coupling with compact footprint and minimal energy dissipation. However, an ideal Ising machine would associate both types of nanodevices, and they must operate synergistically to support annealing: a progressive reduction of machine stochasticity that allows it to settle to an energy minimum. Here, we report an Ising machine that combines two nanotechnologies: memristor crossbar – storing multi-level couplings – and stochastic magnetic tunnel junction (SMTJ), acting as thermally driven spins. Because the same read voltage that interrogates the crossbar also biases the SMTJs, increasing this voltage automatically lowers the effective temperature of the machine, providing an intrinsic, analog-native annealing technique. Operating at zero magnetic field, our prototype consistently reaches the global optimum of a 24-vertex weighted MAX-CUT and a 10-vertex, three-color graph-coloring problem using an externally implemented feedback loop. Given that both nanotechnologies in our demonstrator are CMOS-integrated, this approach is compatible with advanced 3D integration, offering a scalable pathway toward compact, fast, and energy-efficient large-scale Ising solvers.

## Abstract (web summary)

Ising machines offer promising solutions for large-scale optimization problems. Iftakher et al. report a hybrid platform combining memristors with stochastic magnetic tunnel junctions to solve the Ising model by integrating multi-level spin couplings, stochastic spin behavior, and voltage-controlled annealing.

## Introduction

The growing demand for rapid solutions to large, combinatorial optimization tasks—including resource allocation, scheduling, and AI decision making—has renewed interest in the Ising model^1^. By mapping a problem onto a network of binary spins that minimize a global Ising Hamiltonian, one can cast many NP hard or NP complete instances—graph partitioning, Boolean satisfiability, traveling salesman tours—into a unified framework^2^. Physical “Ising machines” accelerate this search by allowing interacting elements to relax naturally toward low-energy configurations, and recent electronic demonstrations based on coupled oscillators^3–12^ or probabilistic bits^13–19^ highlight this momentum.

For practical deployment, an Ising machine must satisfy three intertwined requirements. First, it needs dense, reconfigurable memories that store multi-level spin-spin couplings, yet add negligible static power and area. Second, it must anneal—gradually lowering stochasticity during the search—without resorting to power-hungry supervisory logic. Third, because routing resources vanish quickly in two dimensions, both the spins and the coupling matrix should ideally reside in CMOS back-end-of-line (BEOL) layers to enable vertical integration.

Here, we demonstrate experimentally an Ising machine that satisfies all three requirements by associating two CMOS-integrated nanotechnologies. A hafnium oxide memristor crossbar stores the spin-spin couplings, while probabilistic spins are implemented using a perpendicular stochastic magnetic tunnel junction (SMTJ, used as p-bit^20–22^). In our approach, the same read voltage that interrogates the crossbar also biases the SMTJs: lowering this voltage increases spin fluctuations (higher effective temperature), whereas raising it suppresses flips (lower effective temperature). This built-in, device-level mechanism thus provides intrinsic annealing with almost no additional circuitry. Our prototype solves a 24-vertex weighted MAX CUT and a 10-vertex, three-color graph coloring problem. All measurements are performed at room temperature and zero external magnetic field. The memristor array and the SMTJ are both integrated above CMOS circuits, on separate dies, providing a feasible route to future three-dimensional integration.

Prior approaches address the problem only partially, relying on nanodevices to implement either spin dynamics or spin-spin couplings. For example, memristor-based Ising machines deliver dense coupling matrices but emulate spins in software or CMOS^23–27^. Conversely, networks of SMTJ p-bits supply intrinsic randomness yet rely on off-chip or on-chip CMOS for spin-spin couplings^13,14,16,18^. The sole hybrid demonstration to date, combining VO_2_ oscillators with FeFET couplers^28^, lacks an annealing path. By integrating memristors with SMTJs and harnessing a voltage-controlled effective temperature, we unify couplings, spins, and annealing in a compact, vertically integrable architecture.

The remainder of this Article describes the device integration and operating principle, presents experimental optimization results, and discusses scalability toward larger problem sizes enabled by three-dimensional integration.

## Results

### Hybrid Ising machine concept

Our objective is to resolve low-energy configurations of the Ising Hamiltonian:

1 $$H\,=\,-{\sum}_{i < j}{J}_{ij}\,{s}_{i}{s}_{j}\,-\,{\sum}_{i}{h}_{i}\,{s}_{i},$$

where each spin *s*_*i*_ takes binary values. The spin-spin coupling, or adjacency matrix *J* encodes pairwise interactions, while the vector *h* provides on-site biases. The probability of a spin configuration {*s*} obeys Boltzmann-style statistics,

2 $$P\,\left(\{s\}\right)\propto {e}^{-\beta H(\{s\})},\,\beta=\frac{1}{T}$$

with *T* a dimensionless pseudo-temperature. We generate samples with a canonical, sequential Gibbs sampler. Conditioned on all other spins (which we denote *s*_⧹*i*_), the probability for a spin *s*_*i*_ to be one is

3 $$P\,\left({s}_{i}=+ 1| {s}_{\backslash i}\right)\,=\,\sigma \,\left(2\beta {f}_{i}\right),\,{f}_{i}\,=\,{\sum}_{j}{J}_{ij}\,{s}_{j}\,+\,{h}_{i}$$

where *σ* is the sigmoid function, and the local effective field *f*_*i*_ sums contributions from neighboring spins, using a multiply-and-accumulate (MAC) operation weighted by *J*_*ij*_ and the on-site bias *h*_*i*_. By iterating these updates across all spins, the system randomly explores different configurations in proportion to their Boltzmann weight.

To avoid premature trapping in local minima, we employ simulated annealing^29^: we gradually increase the inverse pseudo-temperature *β* over successive updates. Early iterations at low *β* are highly stochastic, encouraging broad exploration; as *β* grows, the dynamics become more deterministic, steering the Ising machine toward low-energy states and—under a sufficiently slow schedule—toward global minima of the Hamiltonian.

**Fig. 1:**
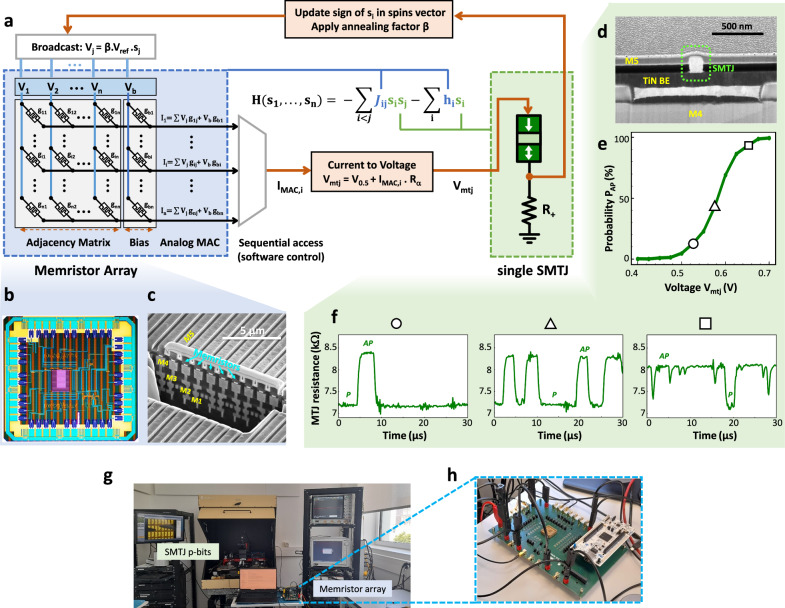
Hybrid memristor-SMTJ Ising machine architecture and device primitives. **a** System-level concept: A hafnium oxide memristor crossbar computes the weighted sum of neighbouring spins, while a stochastic magnetic tunnel junction (SMTJ) converts that current into a probabilistic spin update. This schematic highlights the core computational primitives of the Ising machine. The detailed experimental setups, including mixed-signal circuitry and external instrumentation are presented in Supplementary Note 2. **b** Die micrograph of the 32 × 64 memristor crossbar integrated above CMOS. **c** Scanning Electron Microscopy (SEM) image highlighting the integration of the TiN/HfO_2_/Ti memristors above the fourth layer of metal interconnects. **d** SEM image showing a perpendicular SMTJ integrated in the back-end-of-line (BEOL). **e** Experimentally determined switching probability of an SMTJ versus applied voltage, showing a sigmoid dependence that implements Gibbs sampling. **f** Sample measured time responses of the SMTJ above at *V*_*mtj*_ = 525 mV (circle), 575 mV (triangle), 650 mV (square). **g** Broader view of the experimental setup. **h** The memristor array is mounted on a custom-printed circuit board (PCB) and interfaced with a microcontroller for addressing and communication with the host computer.

Figure 1a provides a high-level conceptual overview of the hybrid hardware platform, which exploits two complementary nanotechnologies to realize the Gibbs sampling steps in Eq. (3) (detailed experimental wiring diagrams are provided in Supplementary Note 2). First, we compute the local field *f*_*i*_ by a MAC operation using a memristor crossbar array. Specifically, as shown in Fig. 1b, the memristors (fabricated from hafnium oxide) are integrated in a hybrid CMOS/memristor circuit, with the memristive devices located in the BEOL layers (Fig. 1c, see “Methods”). When each spin *s*_*j*_ is applied to the corresponding crossbar column, the device conductances (*g*_*ij*_ = *J*_*ij*_ and *g*_*bi*_ = *h*_*i*_) provide the spin-spin coupling and local biasing, while Kirchhoff’s current summation yields:

4 $${I}_{i}\,=\,\left({\sum}_{j}{J}_{ij}{s}_{j}+{h}_{i}\right){V}_{{{{\rm{read}}}}},\,{V}_{{{{\rm{read}}}}}\,=\,\beta {V}_{{{{\rm{ref}}}}}$$

as depicted schematically in Fig. 1d, where *V*_ref_ is a reference read voltage. This equation is here written in the case of nonnegative coupling *J*_*ij*_ and biases *h*_*i*_. (The negative case, which is commonplace in practice, is addressed later in this paper.) This direct electrical summation offers high parallelism and compactness, enabling large-scale networks of spins to be sampled with minimal overhead.

Next, we require a probabilistic element capable of implementing the spin-update rule in Eq. (3): we need to sample a binary value with a probability given by applying a sigmoid function to the MAC result. To achieve this, we leverage stochastic perpendicular magnetic tunnel junction (SMTJ) devices, similar to standard magnetoresistive random-access memory cells but engineered to have extremely low retention times (milliseconds to nanoseconds), making them unstable due to the available thermal noise at room temperature^13,30–32^. As seen in Fig. 1d, these SMTJs are also fabricated in the BEOL of a CMOS process, using an academic process (see “Methods”). These junctions can fluctuate spontaneously between parallel and antiparallel magnetic states under thermal agitation. The probability of each state is modulated by the applied voltage. Figure 1e, f depict how this probability smoothly transitions from near-zero to near-unity as voltage increases, reproducing the characteristic shape of a sigmoid function. Hence, by feeding the summed current from the memristor crossbar [Eq. (4)] as the input voltage of an SMTJ-based “p-bit,” we directly implement the probabilistic update prescribed by Eq. (3). This data was obtained at zero applied magnetic field, and we call *V*_0.5_ the voltage at which the junction has exactly a one half probability of being in each state.

A key advantage of our approach is that increasing the read voltages *V*_read_ naturally increases the effective *β* in Eq. (3). The voltage that we apply to the SMTJ is *V*_0.5_ + *I*_MAC_*R*_*α*_, where *R*_*α*_ is a conversion constant (see “Methods”). Lower *V*_read_ voltages keep the SMTJ voltage near *V*_0.5_, allowing frequent SMTJ switching, and mimicking a high temperature encouraging the system to explore more spin configurations. Increasing the read voltages *V*_read_ proportionally increases the current *I*_MAC_ [Eq. (4)], bringing the MTJ voltage further from the middle point *V*_0.5_. This reduces the likelihood of spontaneous state flips and thereby emulates a lower-temperature regime where spins become more deterministic. The “Methods” section “Annealing schedule” shows the mathematical equivalence between our technique and Gibbs sampling with simulated annealing.

**Fig. 2:**
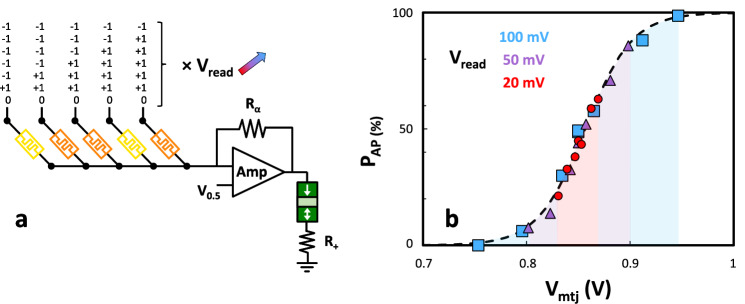
Principle enabling intrinsic annealing demonstrated with direct analog array-to-SMTJ coupling. **a** Schematic of the intrinsic annealing experiment. A partial row of memristors from the memristor array (Fig. 1b), programmed with the color-coded conductances (yellow: 99 *μ*S, orange: 66 *μ*S), are activated with spin inputs, applied as small offsets around a common reference level *V*_0.5_ (spin  × *V*_read_). An operational amplifier wired as current-to-voltage converter biases the SMTJ, and the resulting probability *P*_AP_ is obtained from time traces of the current through the load resistor *R*_+_ (see “Methods”). *V*_0.5_ is the voltage at which the SMTJ states are equiprobable. **b** Measured probability of the anti-parallel state *P*_AP_ as a function of the effective MTJ voltage *V*_mtj_, inferred from the memristor array (*I*_MAC_) and the feedback resistor *R*_*α*_. Data are shown for different read voltages: lower *V*_read_ values produce a more stochastic response, whereas higher *V*_read_ values drive the system toward deterministic plateaus. Thus, *V*_read_ acts as an inverse pseudo-temperature and allows intrinsic annealing. The dashed line is a sigmoidal fit to guide the eye. This experiment uses the full analog coupling setup (see “Methods” and Suppl. Fig. 5).

Figure 2 shows an experimental demonstration of how this intrinsic annealing works. The hybrid CMOS/memristor circuit is physically connected to an SMTJ using an operational amplifier wired as a current-to-voltage converter that naturally generates the voltage *V*_0.5_ + *I*_MAC_*R*_*α*_. We apply six representative spin configurations to the crossbar, yielding six distinct *I*_MAC_ values, and measure the antiparallel-state probability *P*_AP_ of the SMTJ. With a low read amplitude *V*_read_ = 20 mV, the resulting *I*_MAC_ values cluster near zero, keeping *V*_mtj_ close to *V*_0.5_ and *P*_AP_ ≈ 0.5 (high pseudo-temperature). Increasing *V*_read_ to 50 mV and then 100 mV scales the same inputs to larger *I*_MAC_, sweeping *V*_mtj_ further along the sigmoid and expanding *P*_AP_ toward deterministic plateaus near 0 and 1 (low pseudo-temperature). Thus, a single global knob, *V*_read_, continuously tunes the effective *β* in Eq. (3), realizing annealing without requiring any analog-to-digital conversion, which is usually the largest cost of memristor-based in-memory computing^33,34^.

This built-in annealing capability eliminates the need for elaborate external schedules, offering a straightforward means to transition from a stochastic state to a stable, low-energy solution. Consequently, the symbiosis between memristor-based MAC operations and intrinsically stochastic SMTJs provides an elegant solution to the annealing challenge that often constrains large-scale hardware Ising machines.

### Device-level characterization

**Fig. 3:**
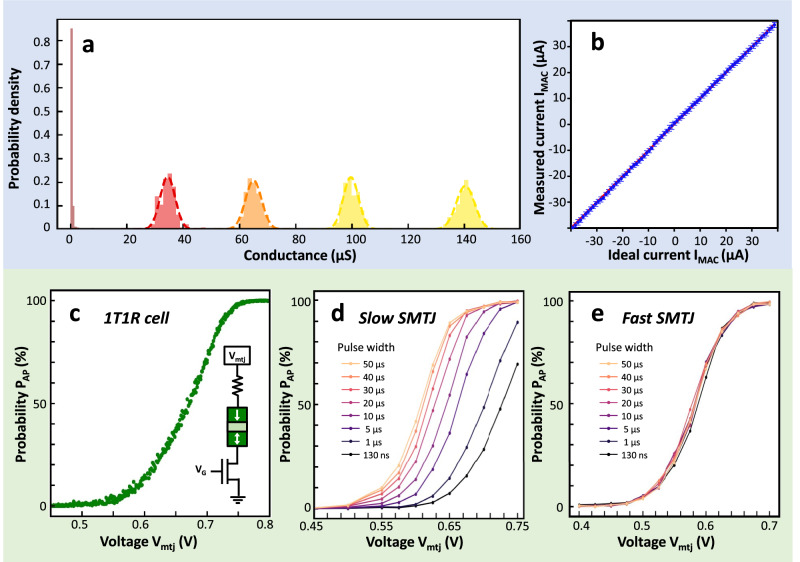
Device-level characterisation underpinning reliable, analog coupling and stochastic spins. **a** Histograms of programmed memristor state, with target conductance states (0, 33, 66, 99, and 140 *μ*S) after programming. **b** Measured multiply-and-accumulate (MAC) output current versus expected value, validating linear summation of *J*_*ij*_ and *h*_*i*_ (see “Methods”). The red line represents y = x, as a guide for the eyes. Error bars represent one standard deviation of the measured *I*_MAC_. **c** Switching statistics of a single-transistor/single-SMTJ (1T1R) bit-cell, as a function of the voltage across the entire 1T1R structure. **d** Pulse-width dependence of the switching probability in a slow SMTJ. The pronounced left-shift of the curves when increasing pulse width, gradually converging to the canonical sigmoidal response (*t*_*pulse*_ > 40 *μ*s), highlights the dynamics of thermally-assisted barrier crossing in this lower-speed device. **e** Same measurements on a faster SMTJ. The traces recorded with pulse widths from 130 ns (instrument-limited minimum) to 50 *μ*s collapse onto a single sigmoid, signalling the device’s suitability for high-throughput probabilistic bit generation. Devices are classified as slow or fast according to the minimum pulse width required for their switching-probability curves to reach full saturation within the accessible voltage window (see “Methods”).

Figure 3a provides further characterization of the hafnium oxide memristors. To achieve stable, finely tuned conductance levels for accurate MAC operations (Fig. 3b), we employ a program-and-verify procedure, which iteratively checks and refines each device’s conductance (see “Methods”). As shown by the histograms, this approach yields narrow distributions of programmed conductance values across the array. Notably, unlike the more common SET-based programming approach—which relies on controlling the compliance current—we instead rely on the RESET mechanism. This method proves superior for long-term device stability, as confirmed by subsequent experiments.

The SMTJs are fabricated through an academic BEOL process on a commercially produced CMOS-integrated circuit (see “Methods”). Figure 3c confirms that the expected sigmoidal relationship between switching probability and voltage is preserved when the device is embedded in an on chip one transistor/one resistor (1T1R) bit cell. To the best of our knowledge, these results constitute the first demonstration of SMTJs monolithically integrated onto a commercial CMOS chip.

Because the academic process does not yet guarantee perfect free-layer thickness uniformity, the SMTJs exhibit significant die-to-die variability. Figure 3d, e present data from devices that switch at different speeds. These measurements, performed at zero magnetic field, reveal the minimum pulse width that still produces a sigmoid-like characteristic for each SMTJ. For every tested pulse duration, we applied 1000 identical read pulses and recorded the fraction of antiparallel (AP) outcomes. In Fig. 3d, pulses shorter than  ~ 2 *μ*s deliver spin transfer torque during a time insufficient to reach the upper tail of the sigmoid curve. A 5 *μ*s pulse already spans almost the full 0–100 % probability range, making it adequate for Ising machine updates, while extending the pulse beyond 40 *μ*s produces no further change—behavior consistent with the modified Néel-Brown thermal activation model^35–37^.

By contrast, the fast SMTJ in Fig. 3e retains an identical sigmoid down to the instrument-limited minimum pulse width of 130 ns, implying that perpendicular SMTJs of this type can support update rates well above 10 MHz. Even faster, nanosecond-scale switching at low voltage has been reported previously in non CMOS processes^38–41^.

### Solving a weighted MAX-CUT instance

**Fig. 4:**
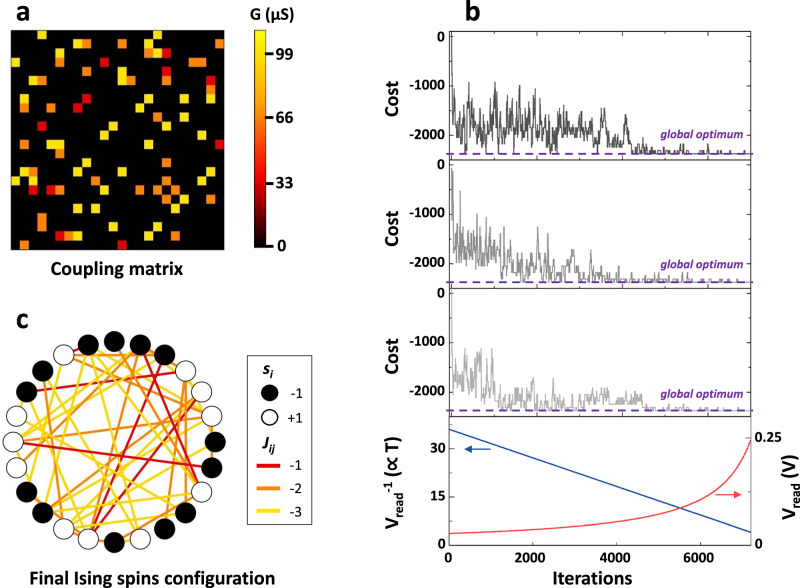
Hardware solution of a 24-node weighted MAX-CUT instance. **a** Conductance map programmed into the memristor array, implementing a three-level *J*_*ij*_ matrix (33, 66, and 99 *μ*S), and *h*_*i*_ = 0). **b** Linear temperature schedule (1/*β*) applied by ramping the read voltage *V*_read_ and evolution of Ising energy during 7200 sequential spin updates; the system escapes local minima early and converges to the optimum cut predicted by a software preliminary study. The three curves are three independent realizations of the experiment (the first two use the same SMTJ, while the third uses a different one). **c** Experimentally obtained final state solving the MAX-CUT task (white node: +1 spin, black node: −1 spin). These experiments use the computer-mediated coupling setup (see “Methods” and Suppl. Fig. 6).

With the device primitives validated, we next ask whether our two-nanotechnologies Ising machine can solve an optimization problem. We first evaluated it on the weighted maximum cut (MAX-CUT) problem, a classic benchmark in the field and an NP-hard task with applications ranging from circuit partitioning to combinatorial optimization. As illustrated in Fig. 4, the objective is to divide a graph’s nodes into two sets that maximize the total weight of edges cut by the partition.

Our experiment (shown in Fig. 1g, h) is performed using the CMOS-integrated memristor array shown in Fig. 1b, c. In our 24 × 24 demonstration, the coupling matrix terms *J*_*ij*_ must support multiple discrete levels (see “Methods”), which we implement directly via the analog conductance states of the memristors (in this instance, all bias terms *h*_*i*_ = 0). In this task, the spins assume values +1 and −1, which translate into voltages +*V*_read_ and −*V*_read_ on the memristor array (see “Methods”). Figure 4a shows the measured adjacency matrix programmed into our memristor array, demonstrating precise setting of conductance values.

To solve MAX-CUT, we evaluate spin values one-at-a-time to carry out the Gibbs sampling steps. We compute the value of the field *f*_*i*_ of one spin using the memristor array. The result is then directly applied to a unique SMTJ (Fig. 1d) to sample a corresponding spin value along Eq. (3). Note that in this experiment, to facilitate data analysis, the coupling between SMTJ and memristor is now computer-mediated: the local field is computed by the memristor array, read out by a computer/controller, and the corresponding feedback/read voltage is then applied to the SMTJ (see “Methods” and Suppl. Fig. 6). The process is then repeated for all spins. We progressively increased *V*_read_ throughout the process, thereby lowering the effective temperature (Fig. 4b, see “Methods”). This annealing schedule enabled the Ising system to escape local minima in early iterations and converge to a stable, low-energy configuration after around 7000 spin updates (Fig. 4c). Across multiple experimental trials (shown in Fig. 4b), the machine reliably found the correct MAX-CUT solution, with some variation in the exact number of iterations required. (We confirmed using a software solver that the final cost obtained experimentally is the task optimum and that the final state is a correct solution.) The three trials shown in Fig. 4b were performed using two different SMTJs (the two first trials use the same SMTJ, and third trial is using a different SMTJ). This reproducibility underscores the system’s robustness and confirms that the hybrid memristor-SMTJ architecture can effectively handle multi-level spin couplings, a key requirement for solving complex, weighted Ising problems.

### Solving a graph coloring instance with bias terms

**Fig. 5:**
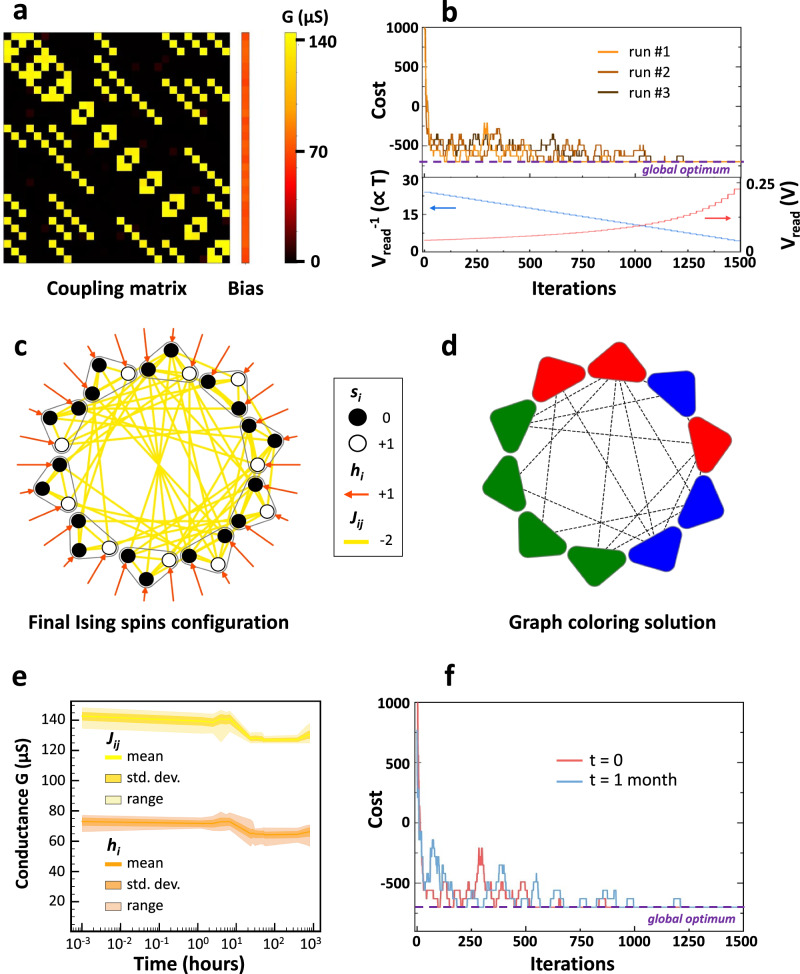
Graph-coloring benchmark solved with the same hardware. **a** One-hot encoding of a ten-node, three-color problem mapped to a sparse 30 × 30 memristor matrix (140 *μ*S negative couplings) plus a 70 *μ*S positive bias column. **b** Linear temperature schedule (1/*β*) applied by ramping the read voltage and evolution of Ising energy during 1500 sequential spin updates. The three curves are three independent realizations of the experiments (the first two use the same SMTJ, while the third uses a different one). **c**, **d** Final state of the machine (**c**, white node: spin of 1, black node, spin of 0), and corresponding color configuration (**d**) in which no adjacent vertices share a color. **e** Mean conductance (solid line), one standard deviation band (darker shading), and full range (light envelope) of the memristor conductances encoding the couplings *J*_*ij*_ (upper traces) and biases *h*_*i*_ (lower traces) measured from 10^−3^ h to 10^3^ h after programming. Both sets remain within a few *μ*S of their initial values over a month, indicating excellent retention of the parameters owing to the RESET-based programming algorithm. **f** Cost versus iteration traces obtained immediately after programming (red) and after one month of room temperature storage (blue) for the same graph coloring instance. The nearly identical convergence behavior and final cost demonstrate that the small conductance drift observed in (**e**) does not degrade optimization performance. These experiments use the computer-mediated coupling setup (see “Methods” and Suppl. Fig. 6).

To further showcase the versatility of our hardware Ising machine, we tested it on the graph coloring problem—another NP-hard task with broad relevance in scheduling, register allocation, and frequency assignment. In this problem, each node must be assigned one of several colors, with the constraint that adjacent nodes cannot share the same color. We targeted a 10-node graph colored with three colors, a setting that can be achieved using purely binary *J*_*ij*_ values and non-zero biases *h*_*i*_ (see “Methods”). Each color assignment is mapped to a spin subsystem, resulting in a 30 × 30 system of coupled spins whose adjacency matrix and biases are programmed onto the memristor array (Fig. 5a). In this task, the spins assume values one and zero. As the coupling values *J*_*ij*_ are nonpositive, we represent the spins using voltages −*V*_read_ and 0 onto the memristor array. Conversely, as the bias is positive, a voltage of *V*_read_ is applied onto the bias column of the memristor array. Note that this technique of choosing the sign of the read voltage to represent the sign of Hamiltonian coefficients only works if all the coefficients in a column have the same sign, which is the case here. For tasks where *J*_*ij*_ takes arbitrary signed values, it would be possible to use differential memristor structures to implement signed Hamiltonian coefficients (see Suppl. Note 6). The same differential technique is typically used to implement signed-weight synapses in memristor-based neural networks^42,43^.

Following the same annealing schedule concept (Fig. 5b), the machine consistently converged to a valid coloring in all experimental trials using different SMTJs (Fig. 5b–d). We also confirmed using a software solver that the final cost is the task optimum. Convergence occurred more quickly than for the MAX-CUT task, reflecting the simpler binary coupling structure in the graph coloring formulation.

We exploited the graph-coloring benchmark to test the long-term retention of the programmed spin-spin couplings and biases. Figure 5e tracks the mean conductance of the coupling (140 *μ*S) and bias (70 *μ*S) memristors over a one-month period while the packaged chip was stored at room temperature in ambient air. The drift remains below 15 *μ*S for both device populations, confirming the excellent stability of the RESET-programmed states. Without re-programming the array, we reran the graph-coloring experiment thirty days later; Fig. 5f shows that the solver still converges to the optimal coloring. (Because the SMTJ probe station was unavailable at that time, the spin-update sigmoid was emulated in software in the thirty-days experiment; only the memristor crossbar was operated in hardware.) This result demonstrates that the modest conductance drift does not impair computational accuracy.

## Discussion

We have shown experimentally that a hybrid memristor/SMTJ platform can solve non-trivial Ising problems while integrating three key functions directly in hardware: multi-level spin-spin couplings, stochastic spins, and voltage-controlled annealing. Both a 24-vertex weighted MAX CUT and a 30-spin graph coloring instance were solved at room temperature and zero magnetic field.

Table 1 places our results in the context of previous nanoelectronic Ising machines. To the best of our knowledge, only one earlier demonstrator combines a nanotechnology for spins and a nanotechnology for spin-spin couplings^28^; however, because its spins are encoded in the discretized phase of oscillators, implementing simulated annealing is a considerable challenge and was not demonstrated. All other prototypes fall back on CMOS for either the spins or the coupling matrix, limiting scalability in latency, area, or energy—points we examine below.

**Table 1:** Hardware implementations of nanotechnology-based Ising machines

Feature	This work	Cai et al.^23^	Jiang et al.^25^	Singh et al.^17^	Kim et al.^26^	Shan et al.^27^
Spins	**Nano (SMTJ)**	Software	Software	Nano (SMTJ-driven clocking of LFSR)	Sign of Bit Line current	Thresholding unit (comparator)
Coupling	**Nano (Memristors)**	Nano (Memristors)	Nano (Memristors)	FPGA	Nano (Memristors)	Nano (Memristors)
Biases	**Nano (Memristors)**	No	No	FPGA	No	No
Annealing Method	**Intrinsic:** *I*_MAC_ **modulation through** *V*_read_	Variable noise level introduced in thresholding function (software)	Gradual non-convexity annealing (software)	*β* adjustable digitally	Bit Line read noise at low *V*_read_ or low G to emulate high temperature	Decaying feedback conductance on the thresholding comparator (hardwired RC delay)
**Problems Solved (hardware size)**	**Weighted MAX-CUT** (24 × 24 **sparse***J*, **1 p-bit w/ TDM**) **Graph Coloring** (30 × 30 **sparse***J*, 30 × 1 *h*, **1 p-bit w/ TDM**)	Binary MAX-CUT (32 × 32 *J*, 50% dense)	Binary and Weighted MAX-CUT, 9-city TSP (64 × 64 *J*)	Invertible Full Adder (5 × 5 *J*, 5 spins clocked by 5 p-bits) Deep Boltzmann Machine (32 × 32 *J*, 32 × 1 *h*, 32 spins clocked by 5 p-bits)	Binary MAX-CUT (32 × 32 *J*, 50% dense)	Binary MAX-CUT (96 × 96 sparse *J*, 96 spins)

Feature	Yin et al.^67^	Borders et al.^13^	Kaiser et al.^14^	Yin et al.^16^	Si et al.^18^	Pantha et al.^28^
Spins	Software	Nano (SMTJ)	Nano (SMTJ)	Nano (SMTJ)	Nano (SMTJ)	Nano (VO_2_-based oscillator)
Coupling	Nano (FeFET)	MCU + DAC	MCU + DAC	MCU + DAC	MCU + DAC	Nano (BEOL FeFET)
Biases	No	MCU + DAC	MCU + DAC	MCU + DAC	MCU + DAC	No
Annealing Method	Software	No	No	No	MCU corrects update current through SMTJ vs. temp.	No
**Problems Solved (hardware size)**	Graph Coloring (16 × 15 *J* after ternary compression)	Factoring 945 (8 spins, coupling hypergraph with up to 4-body interactions)	Invertible Full Adder (5 × 5 *J*, 5 p-bits)	Factoring 945 (1 p-bit w/ TDM)	9-city TSP (81 × 81 *J*, 81 × 1 *h*, 81 p-bits)	Binary and Weighted MAX-CUT (24 bidirectional coupling elements, 50% density, 10 spins)

*SMTJ* stochastic magnetic tunnel junction, *TDM* time-division-multiplexed, *MCU* microcontroller unit, *DAC* digital-to-analog converter, *FPGA* Field-Programmable Gate Array, *TSP* traveling salesman problem.

In the sequential experiments reported here, the achievable time-to-solution is set by the laboratory instruments driving *V*_read_. Each spin is updated with a 50 *μ* s read pulse. With this pulse width, the 24-node MAX-CUT instance requires 7200 sequential updates (300 sweeps), corresponding to an intrinsic hardware time of about 0.36 s, and the 10-node, 3-coloring instance requires 1500 updates (50 sweeps), corresponding to about 0.075 s. However, our approach, in a final integrated version, has the potential to be orders of magnitude faster than current electronic Ising machines, for several reasons.

Our present prototype uses Gibbs sampling, with spins sampled one at a time. Still, it is fully compatible with parallel operations as the memristor array naturally computes the field of several spins simultaneously. Achieving such parallelism simply demands a set of physical p-bits that can switch concurrently. SMTJs meet this requirement because a single junction, plus a minimal selector, forms a p-bit; equivalent quality CMOS implementations of p-bits can consume thousands of transistors^17^. Therefore, our approach would operate ideally with the multiple parallel extensions of Gibbs sampling that have been introduced in recent years to accelerate convergence. Chromatic Gibbs sampling updates all non-interacting spins simultaneously by exploiting graph coloring^15,19^; replica-based tempering exchanges configurations among copies held at staggered effective temperatures^44,45^; and fully asynchronous dynamics allow each spin to flip whenever its local conditions are met^13,18,46^. Device-level refinements such as variability aware biasing and in situ learning of couplings^14^ could further boost scalability.

Such a parallel version of our approach has the potential to be very fast. Our work uses, to the best of our knowledge, the first CMOS-integrated SMTJs. Our fastest CMOS-integrated SMTJs sample p-bits in 130 ns, but SMTJs can be much faster. Suppl. Note 6 shows sample measurement of non CMOS-integrated SMTJs using a similar stack^40^ and that switch in nanoseconds. Other groups have also reported non-CMOS-integrated SMTJs that switch in nanoseconds at femtojoule energies^38,39,41^, two to three orders of magnitude better than all-CMOS p-bits^17^. The memristor crossbars, which we use in this work to implement spin-spin couplings, also have the potential for nanosecond operation, because they do not need an ADC at the output: the MAC current feeds into an SMTJ. This means that our approach can, in principle, sample spins in parallel, at nanosecond speeds. Previous studies have shown that the capability to sample spins in nanoseconds and in parallel could reduce time to solution by several orders of magnitude compared with conventional electronic implementations^15,17^.

The final version of our machine also holds the potential for outstanding energy efficiency. Memristor crossbars are the gold standard for energy-efficient MAC operations, and, until now, have been mostly used for low-energy in-memory computing of neural networks^33,42,43,47,48^. For Ising workloads, the benefits are even larger than for neural networks, as we do not need an ADC: the MAC current directly biases the stochastic p-bits at the periphery. Additionally, the annealing schedule needs only two global DACs (*V*_read_ and −*V*_read_, see Supplementary Note 6), rather than one DAC per input as is usually required for neural networks, so that the annealing logic does not scale with problem size. In Supplementary Note 6, we provide conservative, order-of-magnitude estimates for an integrated implementation, which indicate the high potential of our approach in terms of energy per solution compared to existing CMOS, CMOS + SMTJ, and memristor- or FeFET-based Ising machines.

Moreover, the *V*_read_-dependence of the MAC signal-to-noise ratio aligns naturally with simulated annealing: the lower-SNR regime at small *V*_read_ promotes randomness during the exploratory phase, while the higher-SNR regime at large *V*_read_ suppresses noise as the system settles. This behaviour is consistent with the measurements presented in Supplementary Note 4.

Finally, when the target Ising graph is sparse—as in our benchmarks, where 85.4% of MAX CUT and 82% of graph coloring couplings were zero—most memristors can remain in a high resistance, near zero power state, making the architecture energetically advantageous. More generally, there is no intrinsic sparsity requirement: dense coupling matrices are naturally supported by the crossbar, with the main practical difference being the larger aggregate MAC current and the correspondingly reduced noise margin at low *V*_read_. As discussed in Supplementary Note 1, minor adjustments to the annealing schedule compensate for this effect.

In our current prototype, the memristor arrays and SMTJ-based stochastic units are separated by centimeter-scale PCB traces and laboratory-bench cabling. Achieving the targeted nanosecond-level coupling, therefore, requires tighter integration, eliminating all off-board interconnects. Because both devices are already fabricated in the CMOS BEOL, they are compatible with multiple advanced integration techniques. A pragmatic roadmap for scaling our machine begins with 2.5-D integration: relocating the two dies onto a silicon interposer removes board-level parasitics while leveraging existing advanced-packaging infrastructure. The next milestone is 3-D hybrid bonding, in which prefabricated wafers are joined face-to-face through copper-copper/oxide-oxide bonds, delivering sub-micron pitches without the cumulative thermal budget of sequential BEOL steps^49,50^. The ultimate goal is monolithic 3-D integration, where layer-by-layer fabrication places both devices in the same BEOL stack. Demonstrations of multi-tier memristor arrays connected by sub-100 nm vias confirm the manufacturability of such stacks^51–53^, and technology roadmaps identify monolithic 3-D as pivotal for future high-density, high-bandwidth compute-in-memory and neuromorphic architectures^54,55^. Across this progression, interconnect lengths shrink from millimeters (2.5-D) to micrometers (hybrid bonding) and ultimately to sub-micrometers (monolithic 3-D), bringing memory-access latencies in line with the intrinsic nanosecond dynamics of SMTJs.

A further consideration for scalability is the resolution of memristor conductance values. The RESET- programmed HfO_*x*_ memristors used here met the accuracy demands of our benchmarks, but larger or more intricate problems may require finer conductance granularity. Higher precision analog memristors^56–58^ or multi-cell techniques already employed in neural networks accelerators^47,48^ can supply that resolution. Such devices would also enable in situ training and dynamic retuning of couplings^59^, broadening the scope of hardware Ising machines.

In addition, practical large-scale deployments must accommodate device-to-device dispersion in both the memristor conductances and the SMTJ sampling characteristics. As quantified in Supplementary Note 1, variations in memristor programming, SMTJ offset (*V*_equi_), SMTJ slope (*S*_*v*_), and read-voltage noise all produce only modest degradation for realistic variability levels, and have straightforward mitigation paths (tighter program-and-verify, multi-cell weights, or simple *V*_0.5_ calibration). Supplementary Note 5 further shows that SMTJ transfer curves are temporally stable (drift < 2 mV over five days), indicating that long-term evolution of p-bit characteristics does not materially impact convergence.

The present prototype uses a single SMTJ that is time-multiplexed across spins, a constraint imposed by the wiring of this first demonstrator rather than by the computational model itself. Future implementations would instantiate one SMTJ per spin so that many updates can occur concurrently, as illustrated by the prospective parallel architecture in Supplementary Note 6.

General signed couplings are also supported in hardware. In the present prototype, the sign of each weight is implemented through voltage polarity and column assignment; for fully mixed-sign instances, the standard differential-pair encoding (*g*^+^, *g*^−^) can be used, as detailed in Supplementary Note 6.

Scalability is also bounded by the practical size of analog in-memory computing tiles: interconnect resistance and capacitance cause IR drops and RC delays that distort the effective couplings and slow the analog settling dynamics. These limitations are well known in in-memory neural-network accelerators^60^. A standard mitigation is to avoid very large crossbars and instead tile the coupling matrix into moderate-sized arrays, then synthesize larger MAC operations by aggregating per-tile partial sums, optionally with per-tile calibration to compensate systematic attenuation and offsets^61^. Such composition can be realized either with reconfigurable, fully analog interconnect fabrics that avoid ADCs^48^ or with hybrid analog/digital schemes that digitize at tile boundaries^47^. These approaches are highly effective: in-memory-computing neural networks with millions of weights can approach software accuracy^47,48,62^. For the present Ising machine, the ADC-less approach of Ref. ^48^ is particularly appealing because the analog sums can directly bias SMTJ p-bits, preserving end-to-end array-to-spin analog coupling while keeping annealing under global control.

Scaling also increases sensitivity to nominally zero couplings. For example, in an  ≈ 800-spin graph-coloring study (Supplementary Note 8), small residual offsets on programmed-zero entries accumulate over many terms and can effectively act as a dense background coupling. We show that in Supplementary Note 8 that choosing a differential memristor encoding, beyond enabling signed couplings, mitigates this effect by suppressing common-mode contributions and re-centering near-zero weights, so that residual offsets largely cancel in the MAC. This mapping preserves high success rates (> 90%) for this instance under realistic variability.

By unifying memory, compute, and randomness in a vertically integrable stack, hybrid memristor-SMTJ hardware offers a credible path toward compact, highly energy-efficient optimizers capable of tackling real time scheduling, embedded AI, and scientific computing tasks that are currently impractical on von Neumann machines. This work paves the way for a fully integrated prototype that would shift the state of the art in hardware accelerators for combinatorial optimization.

## Methods

### Fabrication of the memristor arrays

The memristor arrays used in our experiments were fabricated by following a three-phase process flow, building on techniques reported in previous integrated-circuit studies^63–65^. In the first phase, the complementary metal-oxide-semiconductor (CMOS) portion was produced at a commercial foundry employing a 130-nm low-power process with four metal interconnect layers. During the second phase, the hafnium oxide (HfO_*x*_) memristors were integrated between the fourth and fifth metal layers. Each memristor features a stacked structure of titanium nitride (TiN), HfO_*x*_, titanium (Ti), and a top TiN layer, with the 10-nm HfO_*x*_ active layer deposited by atomic layer deposition. The Ti interface layer was also set to 10 nm, while each memristor device was patterned to a 300-nm diameter. Finally, a fifth metal interconnect layer was deposited above the memristors, aligned over vias to ensure reliable electrical contact.

In the third phase, the completed wafers underwent packaging in J-leaded Ceramic Chip Carrier modules, handled by a commercial vendor. This packaging step ensures robust protection and facilitates efficient testing, eliminating the need for specialized probe stations or probe cards in subsequent measurements.

### Design and programming of the memristor arrays

Our memristor platform is designed for unconventional, memristor-based computations. It comprises a 32 × 64 two-transistor-one-resistor (2T1R) array, where each cell includes two transistors and one memristor. Although the array is capable of both vertical and horizontal transistor addressing, here we operate it in a 1T1R configuration by employing only the horizontal addressing. The on-chip peripheral circuitry supports multiple operational modes. (1) A device-characterization mode provides the infrastructure to develop forming and programming techniques, which we used in this study. (2) An analog computing mode enables the memristors to perform MAC operations by multiplying an input voltage vector with memristor conductance values. In our work, this capability underpins the Ising Hamiltonian operations, representing spins by the former and encoding the adjacency matrix through the latter. Additional modes of operation, not relevant to the present experiments, are also available but were not utilized here.

Forming and programming pulses were applied by a Keysight B1530A waveform generator/fast measurement unit. We formed each memristor once, applying twenty-microsecond voltage pulses of gradually increasing amplitude until the device reached a resistance of approximately 10 k*Ω*. For subsequent programming, we adopted a program-and-verify scheme, issuing successive ten-microsecond RESET pulses (voltages ranging between 2.0 and 2.5 V) until achieving the target conductance. The verification step used the Keysight B1530A in fast IV mode, with a multi-second delay after each pulse to ensure stable settling of the device’s resistive state, following guidelines in ref. ^66^. Unlike^66^, however, our approach relies on RESET pulses (rather than SET pulses) to achieve more stable and reproducible conductance levels. More technical details on the memristor-programming procedure are provided in Suppl. Note 7.

### Fabrication of the stochastic magnetic tunnel junctions

As with the memristor arrays, the fabrication of the SMTJ chip used in our experiments begins with a CMOS process, followed by the deposition of four metal interconnect layers. This front-end fabrication is performed at a commercial foundry using a 130-nm process. After forming a TiN bottom electrode with low surface roughness, the 200-mm wafers are downsized to 50 mm for subsequent processing. The magnetic tunnel junction stack is deposited in the following sequence: Ta(7)/W(1.5)/CoFeB(1.1)/MgO(RA = 6.7 Ω.*μm*^2^)/CoFeB(1.1)/W(0.3)/[Co(0.6)/Ta(0.2)/Pt(1.1)]_3_/Co(0.6)/Ru(0.9)/Co(0.6)/[Co(0.5)/Pt(0.25)]_6_/Ru(5), where subscripted brackets indicate the number of repetitions in each multilayer, and numbers in parentheses denote nominal thicknesses in nanometers (RA corresponds to the resistance-area product, which strongly depends on the thickness of the insulating MgO layer). The first CoFeB layer is the free layer; the second one is the reference layer, which is strongly pinned by the neighboring synthetic antiferromagnetic (SAF) structure. The SAF is composed of two Co/Pt multi-layer blocks antiferromagnetically coupled through a Ruderman–Kittel–Kasuya–Yosida (RKKY) interaction, provided by a thin Ru interface layer separating the two blocks.

Following stack deposition, the SMTJs are patterned by electron beam lithography with a nominal diameter of 60 nm. From electrical characterization, we estimate an electrical diameter eCD = 36 nm. Each junction is locally encapsulated with a spin-on polymer, which is patterned and then recessed to expose the top of the conductive hard mask. A fifth interconnect layer is subsequently defined using Cr/Al 200-nm metallization and a lift-off process.

After patterning, the effective perpendicular anisotropy of the free layer is sufficiently small that its magnetization can switch in a stochastic manner because of the available thermal noise when a low current is applied, at room temperature and at zero magnetic field. The effective perpendicular anisotropy remains positive, allowing to maintain the magnetization out-of-plane.

### Measurements of stochastic magnetic tunnel junctions

#### Initial device characterization

To extract the voltage-dependent switching characteristics of the studied magnetic tunnel junctions, we applied voltage pulses across an electrical branch composed of the SMTJ in series with a static resistor *R*_+_ of 1 k*Ω*. To evaluate the resistance of the SMTJ, we sample the current *I*_mtj_ flowing in the branch at given times using a Keysight B1530A waveform generator/fast measurement unit. The investigated SMTJs exhibit two metastable resistance states: a low-resistance parallel state (*R*_P_) and a high-resistance antiparallel state (*R*_AP_). We determine the state of the junction by comparing its measured resistance to the mean point between *R*_P_ and *R*_AP_, i.e., $\overline{{R}_{{{{\rm{mtj}}}}}}=\frac{{R}_{{{{\rm{AP}}}}}+{R}_{{{{\rm{P}}}}}}{2}$

The waveforms presented in Fig. 1f show measurements where the state of the junction is sampled every 130 ns. To obtain the sigmoids-like curves presented in Figs. 1e and 3, for each input voltage *V*_mtj_, we applied 1000 voltage pulses of duration 50 *μ*s to the device, with current measurements at times 130 ns, 1, 5, 10, 20, 30, 40, and 50 *μ*s. We plot the proportion of these measurements where the junction was in the AP state. (Fig. 1e shows this proportion at time 50 *μ*s, while Fig. 3d, e show it at all measured times).

In the resulting curves, we see that as the voltage pulse amplitude *V*_mtj_ is swept from lower to higher values, the extracted probability *P*_AP_ increases from zero to one following a sigmoid-like pattern. We fitted this dependence of *P*_AP_ with respect to *V*_mtj_ by a sigmoid function:

5 $${P}_{{{{\rm{AP}}}}}({V}_{{{{\rm{mtj}}}}})=\frac{1}{1+{e}^{-K({V}_{{{{\rm{mtj}}}}}-{V}_{0.5})}}$$

Here, *K* and *V*_0.5_ are constant parameters of the fit, which respectively control the slope (positive) of the sigmoid function, and the voltage at which *P*_AP_ is exactly 0.5. This empirical relationship forms the basis for our device model for simulation and informs subsequent inference-phase parameters.

Devices were categorized as fast or slow according to the minimum pulse width required for their switching-probability curves to reach full 0–100% saturation within the accessible voltage window. Junctions whose response saturated at sub-microsecond pulses were labeled fast, while those requiring microsecond-scale pulses were labeled slow.

#### When performing optimization tasks

When the machine operates in inference mode, the memristor crossbar outputs a MAC current *I*_MAC_. This current is converted to a voltage pulse across the SMTJ branch using

6 $${V}_{{{{\rm{mtj}}}}}={V}_{0.5}+{I}_{{{{\rm{MAC}}}}}{R}_{\alpha }$$

where *R*_*α*_ is a conversion constant. In our experiments, we use a value ${R}_{\alpha }=2\left(\overline{{R}_{{{{\rm{mtj}}}}}}+{R}_{+}\right)$.

In all optimization experiments reported in this work, the memristor array is biased around the same reference level *V*_0.5_ that defines the equiprobable point of the SMTJ. The column voltages that encode the spins are implemented as small signed offsets around *V*_0.5_. Thus, when we refer in the main text and Supplementary Information to applying +*V*_read_, −*V*_read_, or 0 to a column of the memristor array, the corresponding absolute voltages are *V*_0.5_ + *V*_read_, *V*_0.5_ − *V*_read_, and *V*_0.5_, respectively. Because the MAC current depends only on voltage differences, this common-mode shift by *V*_0.5_ does not modify Eq. (6) or the expressions for the local fields; it simply ensures that *V*_mtj_ remains centered around *V*_0.5_.

In the experiments of Fig. 2, the memristor array and the spintronic magnetic tunnel junction (SMTJ) are directly coupled through an analog MCP6002 operational amplifier (see Fig. 2a and Suppl. Fig. 5), wired as a current-to-voltage converter, which implements Eq. (6) intrinsically.

The full annealing experiments shown in Figs. 4 and 5 were performed using the computer-mediated configuration of Suppl. Fig. 6. In this arrangement, a Keysight B1530A waveform generator/fast measurement unit measures *I*_MAC_, the control computer computes the corresponding *V*_mtj_ following Eq. (6) and a second Keysight B1530A channel applies this voltage to the SMTJ. This setup has two advantages for a proof-of-concept experiment: (i) it logs every voltage and current in the setup, enabling advanced analysis of the results; and (ii) it is configured with a compliance that prevents applying excessive *V*_mtj_, thereby protecting the SMTJ from experimental errors in long experiment sessions. For each 50-*μ*s reading pulse, we acquire a single measurement of the SMTJ’s resistance, which is then converted into a binary state by comparing it to the threshold resistance level $\overline{{R}_{{{{\rm{mtj}}}}}}$.

### Experimental setup

The memristor-array integrated circuit was mounted on a custom-printed circuit board (PCB) and interfaced with an STMicroelectronics STM32F746ZGT6 microcontroller, which provided digital I/O signals for array addressing and communication with the host computer. Two Keysight B1530A waveform generator/fast measurement channels supplied programming pulses for the memristors, as well as the *V*_read_ signal during analog MAC operations. A Keithley 2230G triple-channel power supply provided stable bias voltages for all on-board components.

For the SMTJ measurements, individual SMTJ dies were probe-tested using a 25-probe card. A 1 k*Ω* external resistor *R*_+_was added in series to permit real-time observation of the SMTJ’s binary fluctuations on the oscilloscope. All equipment was orchestrated through a Python-based environment in a Jupyter notebook, which ran the MAX-CUT and graph-coloring experiments.

### Mapping tasks to the Ising model

Our benchmark graphs were synthetically generated with sizes and weight distributions selected to match the throughput of our hardware experiment. For each instance, an exhaustive enumeration on a classical workstation confirmed that the solution obtained by our machine experimentally was the global optimum.

### Weighted MAX-CUT

For a graph $G=({{{\mathcal{V}}}},{{{\mathcal{E}}}})$ with vertex set ${{{\mathcal{V}}}}=\{1,\ldots,N\}$ and weighted edges ${W}_{uv} > 0\,(u,v)\in {{{\mathcal{E}}}}$, MAX-CUT maximizes $\frac{1}{2}{\sum }_{(u,v)\in {{{\mathcal{E}}}}}{W}_{uv}\left(1-{s}_{u}{s}_{v}\right)$ with binary spins *s*_*u*_ ∈ {−1, +1}. Discarding the constant term yields the Ising Hamiltonian

7 $$H=A{\sum}_{(u,v)\in {{{\mathcal{E}}}}}{W}_{uv}\,{s}_{u}{s}_{v}$$

where the positive scale factor *A* is arbitrary and can be used to match device ranges. Identifying the generic Ising form *H* = −∑_*i*<*j*_ *J*_*ij*_ *s*_*i*_*s*_*j*_ − ∑_*i*_*h*_*i*_*s*_*i*_, we directly obtain

8 $${J}_{uv}=-A\,{W}_{uv},\,{h}_{i}=0$$

Each non-zero *J*_*uv*_ is realized by programming the corresponding memristor to one of three conductance levels (33, 66, and 99 *μ*S), proportional to the allowed edge weights. Because the spin-spin couplings *J*_*ij*_ are nonpositive, we apply the read voltage with inverted polarity: a logical spin *s* = +1 is driven by −*V*_read_ and *s* = −1 by +*V*_read_. The MAX-CUT formulation contains no on-site fields, hence the bias column is left unprogrammed (*h*_*i*_ = 0).

### Mapping graph coloring to the Ising Hamiltonian

We consider an undirected graph $G=({{{\mathcal{V}}}},{{{\mathcal{E}}}})$ with $| {{{\mathcal{V}}}}|=N$ vertices and a fixed palette of *C* colors. A one-hot binary variable *s*_*v*,*k*_ ∈ {0, 1} is introduced for each vertex $v\in {{{\mathcal{V}}}}$ and color *k* ∈ {1, …, *C*}: *s*_*v*,*k*_ = 1 if and only if vertex *v* is painted with color *k*. The problem is cast in quadratic unconstrained binary optimization form following ref. ^2^:

9 $$H=A{\sum}_{v\in {{{\mathcal{V}}}}}{\left(1-{\sum}_{k=1}^{C}{s}_{v,k}\right)}^{2}\,+\,A{\sum}_{(u,v)\in {{{\mathcal{E}}}}}{\sum}_{k=1}^{C}{s}_{u,k}\,{s}_{v,k}$$

where the first term enforces the one-hot condition (exactly one color per vertex) and the second term penalizes equal colors on adjacent vertices. Expanding the square in (9) and using ${s}_{v,k}^{2}={s}_{v,k}$ yields

10 $$H=2A{\sum}_{v\in {{{\mathcal{V}}}}}\,{\sum}_{k < c}{s}_{v,k}{s}_{v,c}+2A{\sum}_{(u,v)\in {{{\mathcal{E}}}}}{\sum}_{k=1}^{C}{s}_{u,k}{s}_{v,k}-A{\sum}_{v\in {{{\mathcal{V}}}}}{\sum}_{k=1}^{C}{s}_{v,k}+\,{{{\rm{const}}}}\,.$$

To match the standard Ising notation *H* = − ∑_*i*<*j*_ *J*_*ij*_*s*_*i*_*s*_*j*_ − ∑_*i*_*h*_*i*_*s*_*i*_ we index the binary variables as *i* = *C* (*v* − 1) + *k* and *j* = *C* (*u* − 1) + *c*, so that the coefficient mapping is

11 $${J}_{ij} 	=-2A\,\,{{{\rm{if}}}}\,\left(v=u\,{{{\rm{and}}}}\,k\ne c\right)\,\,\,({{{\rm{one}}}}\; -\; {{{\rm{hot}}}})\\ {J}_{ij} 	=-2A\,\,{{{\rm{if}}}}\,\left(u,v\right)\in {{{\mathcal{E}}}}\,{{{\rm{and}}}}\,k=c\,\,({{{\rm{adjacency}}}})\\ {h}_{i} 	=\,\,\,\,\, A\,\,\,\,{{{\rm{for}}}}\; {{{\rm{all}}}}\,i.$$

All non-zero couplings therefore share the same magnitude ∣ *J*_*ij*_∣ = 2*A* and are negative, favoring opposite spin values. In hardware, we realize these entries with identical conductances (2*A* = 140 *μ*S in our prototype) and drive them with −*V*_read_. The positive fields *h*_*i*_ = *A* are implemented with a dedicated bias column (*A* = 70 *μ*S) accessed by +*V*_read_. The resulting *J* matrix is sparse—every row contains at most *C* − 1 intra-vertex links and $| {{{\mathcal{N}}}}(v)|$ inter-vertex links.

### Graph generation procedure

Random graph instances were generated in Python using NumPy, following a common procedure for both MAX-CUT and graph-coloring tests. Each problem was first specified by a symmetric adjacency (or coupling) matrix. We sampled the upper-triangular entries according to a chosen sparsity and a discrete set of allowed weights: for each vertex pair (*i*, *j*), a uniform random draw determined whether an edge was present, and if so, a weight was selected from the predefined values. The matrix was then symmetrized with a zero diagonal. For the MAX-CUT experiment, we used sparse weighted graphs whose discrete edge weights matched the conductance levels supported by the hardware. For graph coloring, a smaller constraint graph was first generated and then expanded into its 3-coloring formulation by assigning three spins per vertex and adding a bias vector to enforce the one-hot condition. All random instances were generated with fixed seeds for reproducibility. The exact matrices used in the experiments are provided in Suppl. Note 3.

### Annealing schedule

We implement annealing by raising the voltage applied to the memristor crossbar in proportion to the inverse pseudo-temperature parameter *β*. Specifically, we define

12 $${V}_{{{{\rm{read}}}}}=\beta \,{V}_{{{{\rm{ref}}}}}$$

We exploit the fact that our SMTJs exhibit a voltage-dependent probability [Eq. (5)]. If we reinject the voltage *V*_mtj_ [Eq. (6)] in Eq. (5), and the result of the MAC operation [Eq. (4)], we find

13 $${P}_{{{{\rm{}}}}AP{{{\rm{}}}}}=\frac{1}{1+{e}^{-\beta K{V}_{{{{\rm{ref}}}}}{R}_{\alpha }\left({\sum }_{j}{J}_{ij}{s}_{j}+{h}_{i}\right)}}$$

This result maps to the equation of Gibbs sampling [Eq. (3)], with *β* playing the role of a pseudo-temperature.

In practice, we use a linear schedule that decreases *T* (or equivalently increases *β*) from a high initial value (corresponding to a read voltage *V*_read_ in the 30–40 mV range), which promotes frequent spin flips, to a low final value (corresponding to a read voltage *V*_read_ in the 250 mV range), where spins become nearly deterministic. The temperature is changed after each full cycle of attempted spin updates across the graph (i.e., every 24 iterations for MAX-CUT; every 30 iterations for graph coloring), forming a uniformly discretized linear ramp. The exact annealing schedules are shown in Figs. 4b and 5b. We use a different schedule for both tasks, as our benchmark graph coloring task can converge faster than our benchmark weighted MAX-CUT task. The annealing schedules were chosen based on prior simulations of the Ising machine. In the present computer-in-the-loop setup, *β* is defined digitally and converted into the corresponding *V*_read_ value applied by an external DC supply, providing direct hardware control of the pseudo-temperature. The solver’s pseudo-temperature is governed by *V*_read_ and its conversion into MAC current through the memristor crossbar. At the beginning of the annealing process, *V*_read_ assumes low values, which might be difficult to generate precisely on-chip, and where memristor conductance can be partly nonlinear. However, precise control of these low voltages is not critical: annealing schedules are tuned empirically to perform robustly across families of problem instances rather than to hit an exact temperature at each iteration, and the smallest *V*_read_ values coincide with the high-temperature phase, where stochastic fluctuations dominate spin updates and damp the effect of small voltage errors.

## Supplementary information

Supplementary Information

Transparent Peer Review file

## Data Availability

The data measured in this study are available from the corresponding author upon request.

## Code availability

The software programs used for modeling Ising machines are available from the corresponding author upon request.
