# Supplementary material for: Intrinsic annealing in a hybrid memristor-magnetic tunnel junction Ising machine
Source: Nat Commun. 2026 Apr 16;17:5246. doi: 10.1038/s41467-026-71844-8 (PMC13260843; doi:10.1038/s41467-026-71844-8)
Supplement: Supplementary file 1 — Supplementary Information [file 41467_2026_71844_MOESM1_ESM.pdf]

Supplementary Information: Intrinsic Annealing in a Hybrid Memristor-Magnetic Tunnel Junction Ising Machine

Mohammed Akib Iftakher[1,+], Hugo Levices[2,+], Kamel-Eddine Harabi[1,2], Adrien Renaudineau[1], Mathieu-Coumba Faye[2], Corentin Bouchard[2], Florian Disdier[3], Bernard Viala[2], Elisa Vianello[2], Philippe Talatchian[3], Kevin Garello[3], Damien Querlioz[1,*], and Louis Hutin[2]

[1]Université Paris-Saclay, CNRS, Centre de Nanosciences et de Nanotechnologies, Palaiseau, France
[2]Université Grenoble-Alpes, CEA, LETI, Grenoble, France
[3]Université Grenoble-Alpes, CEA, CNRS, Grenoble INP, SPINTEC, Grenoble, France
[*]damien.querlioz@universite-paris-saclay.fr
[+]These authors contributed equally to this work

Contents

Supplementary Note 1: Variability and Scalability 1
Supplementary Note 2: Experimental setups 10
Supplementary Note 3: Benchmark tasks solved experimentally 12
Supplementary Note 4: Signal-to-noise ratio of memristor in-memory computation 16
Supplementary Note 5: Stability of stochastic magnetic tunnel junction 17
Supplementary Note 6: Design and device assumptions for projections 18
Supplementary Note 7: Pulse-based programming for mapping matrices 23
Supplementary Note 8: Large-scale Graph-Coloring benchmark on the Les Misérables network 25
Supplementary References 27

Supplementary Note 1: Variability and Scalability

This Note reports simulations that probe the scalability of the hybrid memristor–SMTJ Ising machine beyond the experiments of the main Article. They also quantify how device- and circuit-level imperfections (memristor programming variability, read-voltage noise (V_{read}), and SMTJ transfer-curve deviations) impact solver performance.

As representative benchmarks, we use a weighted MAX-CUT instance from the BiqMac Library[1] (instance *pw09*) and additional MAX-CUT graphs generated with the `rudy` tool from the same library to stress the solver under differing sparsities and weight alphabets. Unless otherwise stated, all simulations use a uniform annealing schedule of 2 400 attempted updates per node, with pseudo-temperature linearly decreased from $T_{\text{start}} = 44$ to $T_{\text{end}} = 4$. Consistent with the hardware mapping in Methods ($V_{\text{read}} = \beta V_{\text{ref}}$ with $\beta = 1/T$), these endpoints correspond to $V_{\text{read}} = 22\,\text{mV}$ and $250\,\text{mV}$, respectively.

Memristor programming variability

We evaluate the effect of programming dispersion by Monte Carlo simulations in which memristor conductances are perturbed with i.i.d. Gaussian noise of standard deviation $\sigma_G \in [0, 5]\,\mu\text{S}$ (Suppl. Fig. 1a). Tests are run on a BiqMac weighted MAX-CUT instance (*pw09*, 100 nodes, 90% density, 10 weight levels) and on a `rudy`-generated instance of identical size and density but restricted to five weight levels. The success rate, defined as the probability of reaching the global optimum at the end of the annealing schedule (2 400 attempted updates per node), is reported in Fig. 1a. The schedule is tuned to stabilize the system at the terminal pseudo-temperature under nominal conductances.

The five-level instance is very resilient, maintaining a $\sim$95% success rate even at $\sigma_G = 5\,\mu\text{S}$. In contrast, the ten-level instance degrades more rapidly (about 90% at $\sigma_G = 2\,\mu\text{S}$ and 60% at $\sigma_G = 5\,\mu\text{S}$). For reference, our current hardware exhibits $\sim$5 µS dispersion (main Fig. 2a); with more sophisticated program-and-verify using the same devices and hybrid reset/set programming, a dispersion of $\sim$2 µS is attainable[2].

Device-level dispersion impacts convergence through two pathways.

(i) Weight mis-mapping: deviations from nominal couplings distort symmetries and weight ratios, reshaping the effective energy landscape and reducing schedule efficacy. When programmed level spacing is tight, distributions can overlap, creating mapping ambiguities; graphs that rely on fine weight resolution are most sensitive (see Suppl. Figs. 1b,c), consistent with the gap between the five- and ten-level curves in Fig. 1a.

(ii) Boundary-spin instability: variability injects row-level noise on the SMTJ input

$$\sigma_{V_{\text{mtj}}} = V_{\text{read}}\, R_\beta \sqrt{\sum_i \sigma_{g_i}^2} \tag{1}$$

where g_i are the row conductances. Spins whose nominal V_{mtj} sits near the sigmoid knee can be driven back into the stochastic region at the end of annealing, triggering late flips (Suppl. Figs. 1d–f). Robust convergence in this regime benefits from a slightly lower terminal pseudo-temperature (or a short constant-temperature tail).

For instances demanding higher parameter precision, higher-fidelity analog memristors[3–5] or multi-

cell weight representations, which are routinely used in analog neural accelerators[6,7], can provide the required effective resolution and margin.

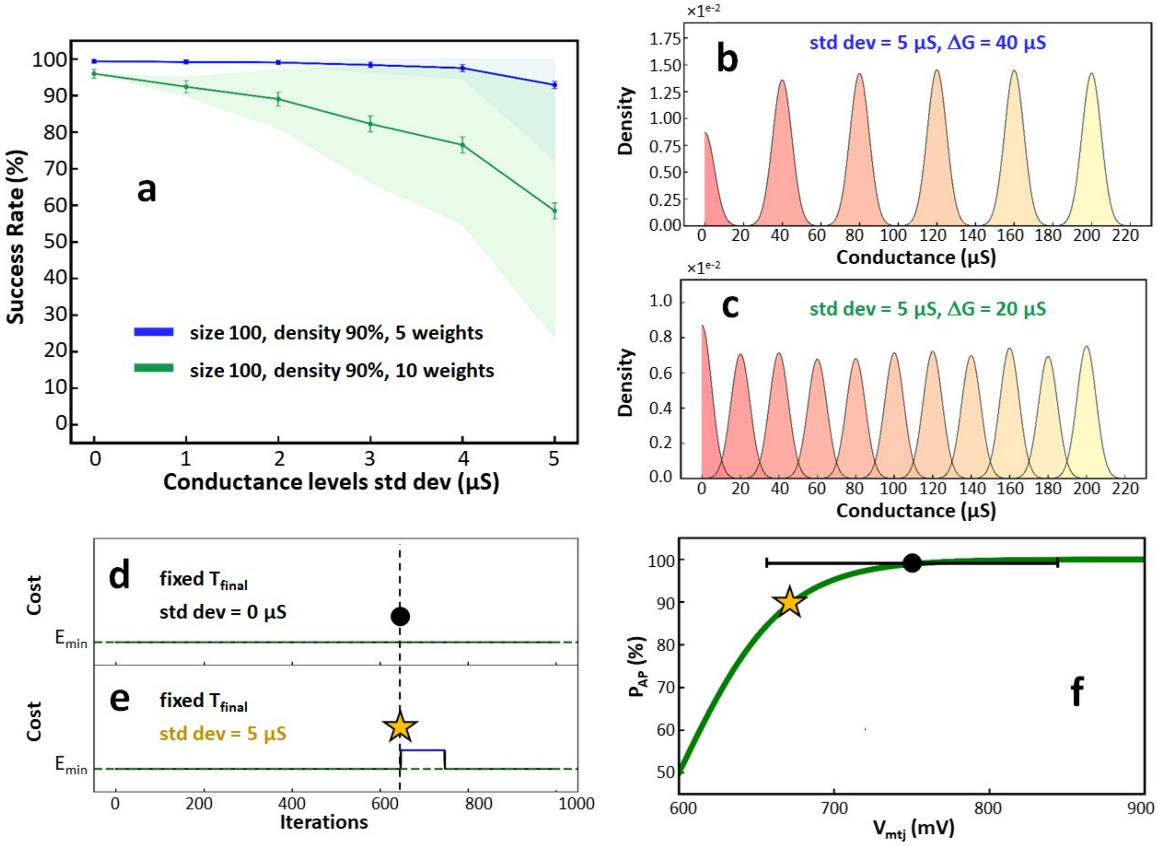

Supplementary Figure 1. **Impact of conductance variability.** **a** Success rate as a function of the standard deviation of programmed conductance values. Each problem instance was solved 1 000 times with fixed noise parameters; the resulting mean and standard deviation (shown as error bars at one standard deviation) represent repeated calculations on the same array of conductance values. Inter-array variability was estimated by resampling the entire conductance matrix 20 times, with the corresponding standard deviation shown as shaded regions. The green curve corresponds to instance *pw09* from the BiqMac library[1], a weighted MAX-CUT problem of size 100, density 90%, and 10 non-zero weight levels. The blue curve corresponds to a `rudy`-generated weighted MAX-CUT instance of the same size and density, but constrained to 5 non-zero weight levels. **b–c** Conductance mapping for the blue-curve instance (**b**) and the green-curve instance (**c**), with variability modeled by Gaussian distributions of standard deviation 5 µS and mean values evenly spaced over a 200 µS range. **d–e** Cost function evolution over 100 attempted updates per node at the lowest pseudo-temperature for the blue-curve instance, with conductance values drawn from distributions of standard deviation 0 µS (**d**) and 5 µS (**e**). **f** Sigmoidal response of the SMTJ. The black marker indicates the nominal input signal, the error bar shows the standard deviation of V_{mtj} for a conductance standard deviation of 5 µS, and the yellow star marks the perturbed input signal corresponding to the unstable node responsible for the switching event on the dashed line in **e**. At the nominal mapping, the final pseudo-temperature was sufficiently low for this point to remain in the deterministic plateau. With conductance variability, however, the input can be shifted back into the stochastic region of the sigmoid, leading to instability and contributing to the observed decrease in success rate, despite the absence of overlap between conductance distributions in **b**.

Noise on memristor array read voltage

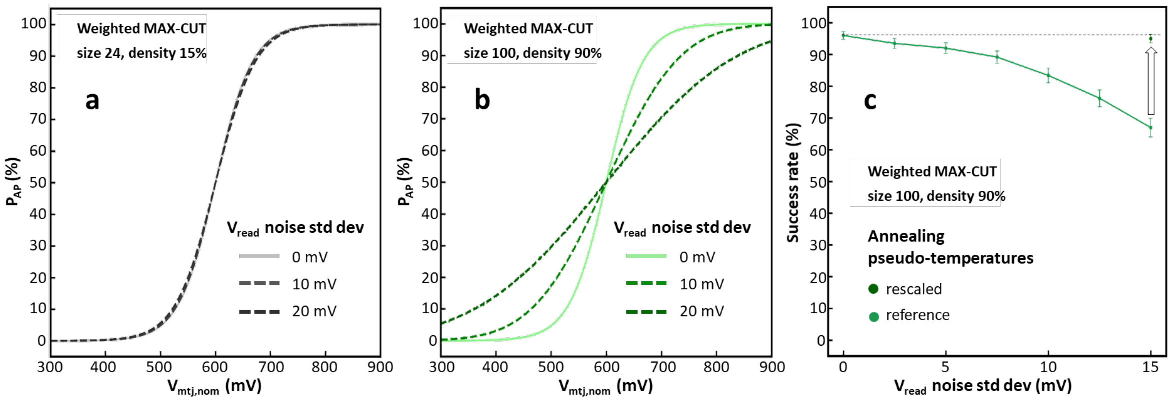

Supplementary Figure 2. Impact of $V_{\mathbf{read}}$ noise. Effective sigmoid response of an SMTJ as a function of the nominal input voltage $V_{\mathrm{mtj,nom}}$, obtained under Gaussian V_{read} noise with standard deviations 0, 10, and 20 mV. **a** The memristor conductances map to a weighted MAX-CUT instance of size 24 and 15% density, solved experimentally in this work. **b** The memristor conductances map to a weighted MAX-CUT instance of size 100 and 90% density, taken from the BiqMac library[1] (instance *pw09*). **c** Success rate of instance *pw09* as a function of the standard deviation of the V_{read} noise. The "reference" and "rescaled" data points correspond to annealing schedules used with and without compensating for the noise-induced broadening of the SMTJ sigmoid, respectively.

In a deployed system, fluctuations on the read amplitude V_{read} can arise from supply noise or shared-bias distribution, unlike our benchtop experiments, where V_{read} is tightly regulated. We quantify the effect by Monte Carlo sampling $V_{\mathrm{read}} = V_{\mathrm{read,nom}} + \delta V$ with $\delta V \sim \mathcal{N}(0, \sigma_V)$ and $\sigma_V \in \{0, 10, 20\}$ mV, for (i) the 24-node, 15%-dense weighted MAX-CUT instance used experimentally and (ii) the reference BiqMac *pw09* graph (100 nodes, 90% density). For each graph, we draw 1 000 noise realizations and evaluate the induced perturbation of the SMTJ input.

A fluctuation δV produces a per-row input variation

$$\delta V_{\mathrm{row}} = R_\beta \sqrt{\sum_i g_i^2}\, \delta V \tag{2}$$

where the sum runs over the conductances g_i in that row. We apply this scaling independently to each row. This hypothesis corresponds to a worst-case scenario: uncorrelated across columns, e.g., when columns are read sequentially, and the noise decorrelates between reads. The effective sigmoid is obtained by averaging the SMTJ response over rows and noise samples as a function of $V_{\mathrm{mtj,nom}}$.

Results are in Suppl. Fig. 2. For the small, sparse instance (panel a) the sigmoid is essentially unchanged up to $\sigma_V = 20$ mV, reflecting the smaller $\sum_i g_i^2$ and hence weaker sensitivity in (2). For the dense 100-node graph (panel b), increasing the noise on V_{read} progressively smooths (flattens) the sigmoid. This flattening is the expected Gaussian convolution of the nominal transfer, effectively reducing its slope around the knee (equivalently, acting as a modest increase in pseudo-temperature near the operating point). Panel c illustrates how this affects the success rate on the same 100-node instance. In the noise-free case, the nominal annealing schedule yields a 96% success rate. Introducing a 15 mV

standard deviation on V_{read} reduces this to 68%. A simple compensation recovers the performance: by uniformly rescaling the pseudo-temperatures according to the ratio of slopes between the zero-noise and $\sigma_V = 15\,\mathrm{mV}$ conditions, the success rate is restored to 95%, indicating that the impact of V_{read} noise can be largely mitigated by an adjustment of the annealing schedule.

SMTJ variability

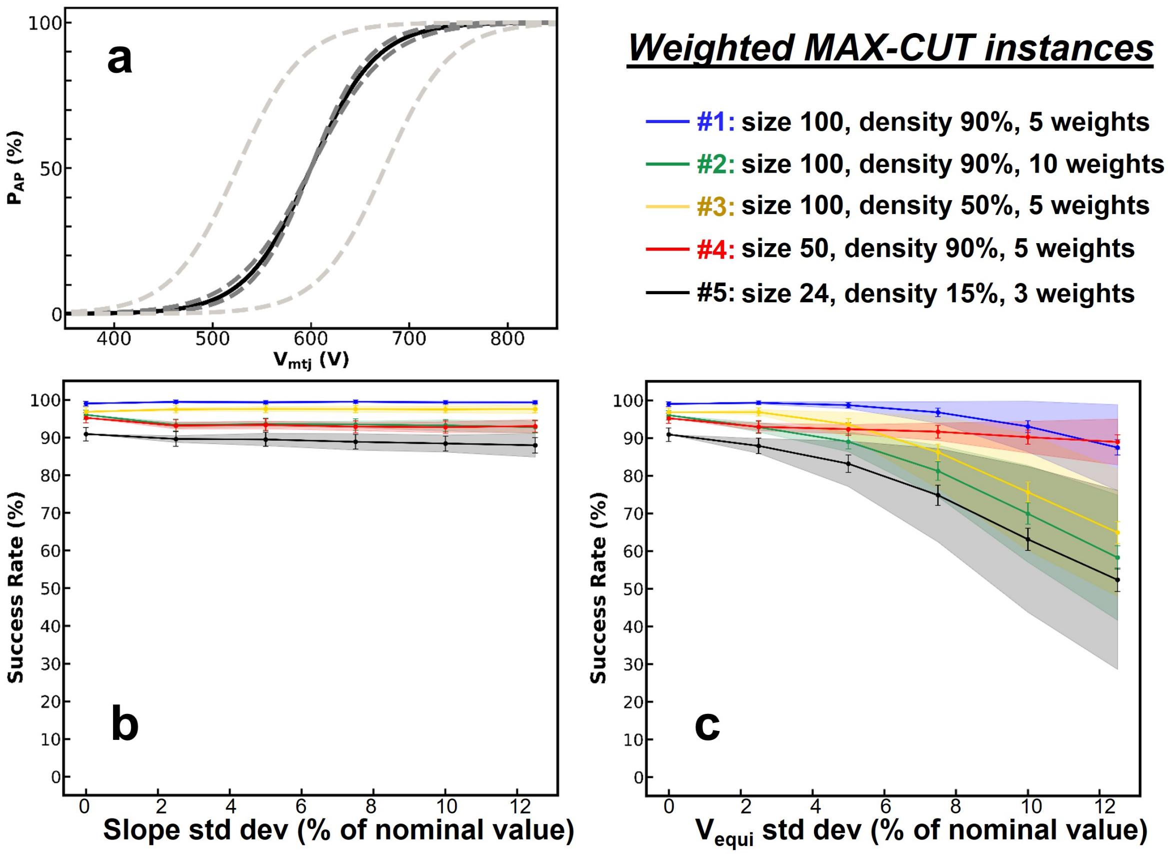

Supplementary Figure 3. Impact of SMTJ slope and offset variability. a Probability of being in the antiparallel (AP) state, P_{AP}, as a function of the applied voltage V_{mtj}. The solid black curve shows the nominal device characteristic, while the dashed grey curves illustrate variability corresponding to $\pm 10\%$ standard deviation in slope factor S_v (darker grey) and offset V_{equi} (lighter grey). **b** Success rate as a function of S_v standard deviation for different weighted MAX-CUT instances, defined by problem size, graph density, and number of weight levels. **c** Success rate as a function of V_{equi} standard deviation. Colored curves correspond to the instances indicated in the legend (#1–#5).

Variations in SMTJ characteristics manifest primarily as shifts of the equiprobable point (offset) and changes in the sigmoid steepness (slope). In an architecture with many concurrently updated spins, such device-to-device differences translate into row-to-row variations of the effective sampling law. We now study these variations and their impact in detail.

In an SMTJ, the probability of the high-resistance (AP) state is

$$P_{AP} = \frac{\tau_{AP}}{\tau_P + \tau_{AP}} \tag{3}$$

with dwell times governed by a Néel–Brown model (no external field)[8]:

$$\begin{cases} \tau_P = \tau_0 \ \exp\left[\dfrac{\Delta E}{k_B T}\left(1 + \dfrac{V_{\text{app}}}{V_c}\right)\left(1 + \dfrac{H_0}{H_k}\right)^2\right] \\ \tau_{AP} = \tau_0 \ \exp\left[\dfrac{\Delta E}{k_B T}\left(1 - \dfrac{V_{\text{app}}}{V_c}\right)\left(1 - \dfrac{H_0}{H_k}\right)^2\right] \end{cases} \tag{4}$$

where V_{app} is the voltage drop directly across the SMTJ (distinct from V_{mtj}, which includes the drop on R_+), ΔE is the SMTJ energy barrier separating the P and AP states, V_c is a critical voltage required to deterministically switch the magnetization in the absence of thermal fluctuations (i.e., at zero temperature), H_0 is the stray field associated with the SMTJ synthetic antiferromagnet, and H_k is the SMTJ anisotropy field.

From Eqs. (3)–(4), we derive the applied voltage V_{equi} where the P and AP states are equiprobable (different from $V_{0.5}$, which includes the drop across R_+):

$$V_{equi} = \frac{-2\frac{H_0}{H_k}V_c}{1 + (\frac{H_0}{H_k})^2} \tag{5}$$

We also derive the slope parameter S_{v} materializing a device-specific voltage sensitivity factor, by differentiating the Néel–Brown expressions for the dwell times at the equiprobable point:

$$S_v = \frac{2\Delta E}{k_B T V_c}\left(1 + \left(\frac{H_0}{H_k}\right)^2\right) \tag{6}$$

To quantify the impact of inhomogeneous p-bit responses, we vary independently the SMTJ offset (V_{equi}) (equiprobable point) and the slope factor (S_v) (voltage sensitivity) and measure solver success rate. We consider a fully parallel setting in which each of the (N) spins is implemented by a distinct SMTJ. For a given MAX-CUT instance of size (N), the pair (V_{equi}, S_v) of each SMTJ is drawn from a Gaussian distribution centered on the nominal value with standard deviation (σ) (independently across devices).

The nominal device operates at $V_{\text{equi}} = 600$ mV and $V_c = 900$ mV (consistent with typical values[9]). The energy barrier is set to $\Delta E \simeq 10 k_B T$, with effective field ratio $H_0/H_k = 0.3$ and $H_0 = 30$ mT. Variations of V_{equi} primarily model changes in the effective field parameters H_0 and H_k via Eq. (5), whereas variations of S_v capture changes in ΔE, T, and V_c through Eq. (6). Representative transfer curves illustrating these perturbations are shown in Suppl. Fig. 3a.

We evaluate a suite of weighted MAX-CUT instances: the BiqMac benchmark *pw09* (100 nodes, 90% density, 10 weight levels), three additional `rudy`-generated graphs spanning sizes, densities, and alphabets, and the instance solved experimentally in the main text. Results are summarized in Suppl.

Figs. 3b,c. Across all cases, success rate is largely insensitive to slope dispersion over the tested range; the annealing schedule tolerates changes in sigmoid steepness because most spins operate on the deterministic plateaus at late times. In contrast, offset dispersion degrades performance: increasing $\sigma_{V_{\text{equi}}}$ lowers the success rate, with sensitivity that depends on the graph (size, density, and weight resolution). Intuitively, V_{equi} variations shift the sampling midpoint of each p-bit, which is equivalent to injecting an additive, device-specific bias on the local field.

This sensitivity is straightforward to mitigate in practice. A simple calibration of $V_{0.5}$, globally for time-division-multiplexed operation, or per-device/per-row for parallel arrays, recenters each sigmoid and restores the nominal mapping.

Summary and comparison of the impact of different imperfections

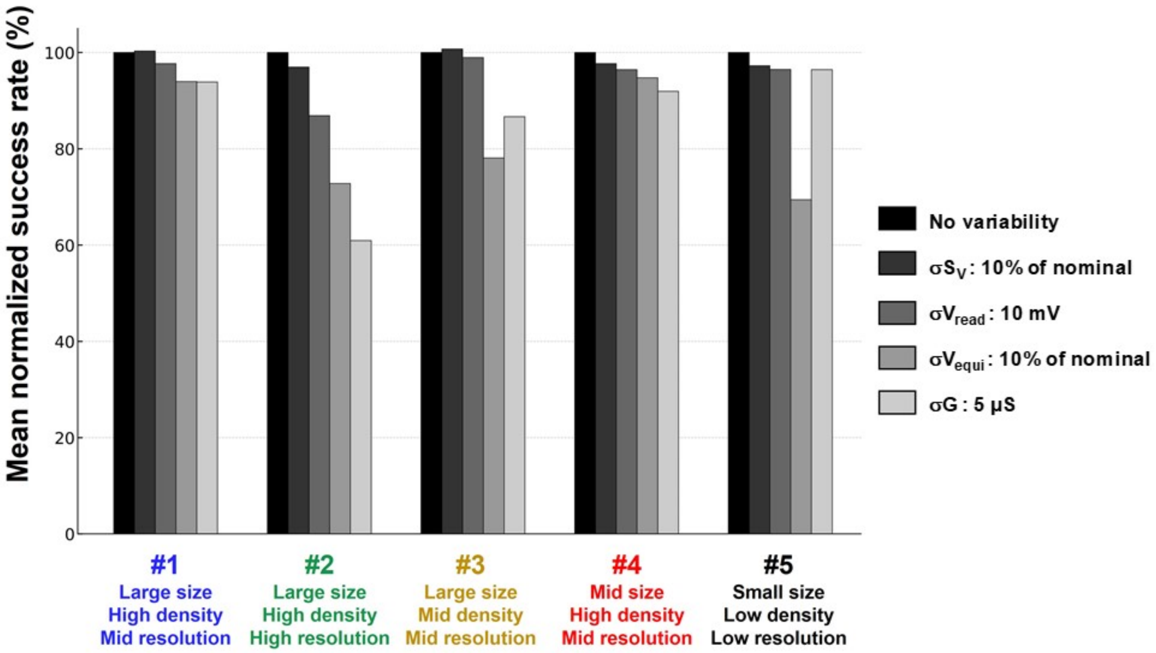

Supplementary Figure 4. Impact of device variability sources on solver performance for weighted MAX-CUT instances. Mean success rate normalized to the nominal (no-variability) case for five representative problem instances (#1–#5), characterized by different graph sizes, densities, and weight resolutions (as indicated below each group). Each bar shows the impact of a specific variability source: slope factor variability $\sigma_{S_v} = 10\%$ of the nominal value, read voltage noise $\sigma_{V_{\text{read}}} = 10$ mV, equilibrium voltage variability $\sigma_{V_{\text{equi}}} = 10\%$ of the nominal value, and conductance variability $\sigma_G = 5$ µS. The un-normalized success rate values corresponding to the black bars all lie between 90% and 100%, as shown in Suppl. Fig. 3 b–c, with differences between instances partly attributable to the use of a single, common annealing schedule.

Suppl. Fig. 4 compares the normalized success rate under four variability sources across five weighted MAX-CUT instances (#1–#5; #2 is the BiqMac reference and #5 is the instance solved experimentally in the main text). Normalization to the zero-variability baseline highlights relative sensitivity independent of absolute difficulty.

As a summary:

- **Slope variability ($\sigma_{S_v} = 10\%$).** Impact is negligible across all instances. Changes in sigmoid steepness primarily affect the vicinity of the knee; late-stage updates occur on deterministic plateaus, so convergence is robust.
- **Read-voltage noise ($\sigma_{V_{\text{read}}} = 10$ mV).** Effect is minor. V_{read} noise acts like a small, zero-mean temperature jitter: early iterations are already high-temperature, and late iterations are dominated by plateau regions. Sensitivity can grow with graph density, but remains modest at this noise level.
- **Conductance dispersion ($\sigma_G = 5$ µS).** This is the dominant degradation for high-resolution instances (dense graphs, many weight levels). Practical mitigations include tighter program-and-verify (RESET-centric with settle delays), multi-cell weight encoding, modest schedule deepening at terminal temperature, or higher-fidelity devices.
- **Offset variability ($\sigma_{V_{\text{equi}}} = 10\%$).** No universal trend across instances; sensitivity depends on graph parameters. This is the easiest imperfection to cancel: per-device (parallel) or global (time-multiplexed) calibration of $V_{0.5}$ can recenter each sigmoid.

Supplementary Note 2: Experimental setups

This supplementary note illustrates the instrument connectivity and signal flow corresponding to the experiments in Figs. 2, 4, and 5 of the main text.

Suppl. Fig. 5 illustrates the configuration used for the experiment in Fig. 2, where the memristor array and the spintronic magnetic tunnel junction (SMTJ) are directly coupled through an analog amplifier.

The full annealing experiments shown in Figs. 4 and 5 were performed using the computer-mediated configuration of Suppl. Fig. 6. In this arrangement, the B1530A parameter analyzer measures I_{MAC}, the control computer computes the corresponding V_{mtj}, limiting it if necessary, and the B1530A parameter analyzer applies this voltage to the SMTJ. This setup has two advantages for a proof-of-concept experiment: (i) it allows logging every voltage and current in the setup, allowing advanced analysis of the results. (ii) it is configured with a compliance to avoid applying excessive V_{mtj} and therefore protecting the SMTJ from experimental errors in long experiment sessions.

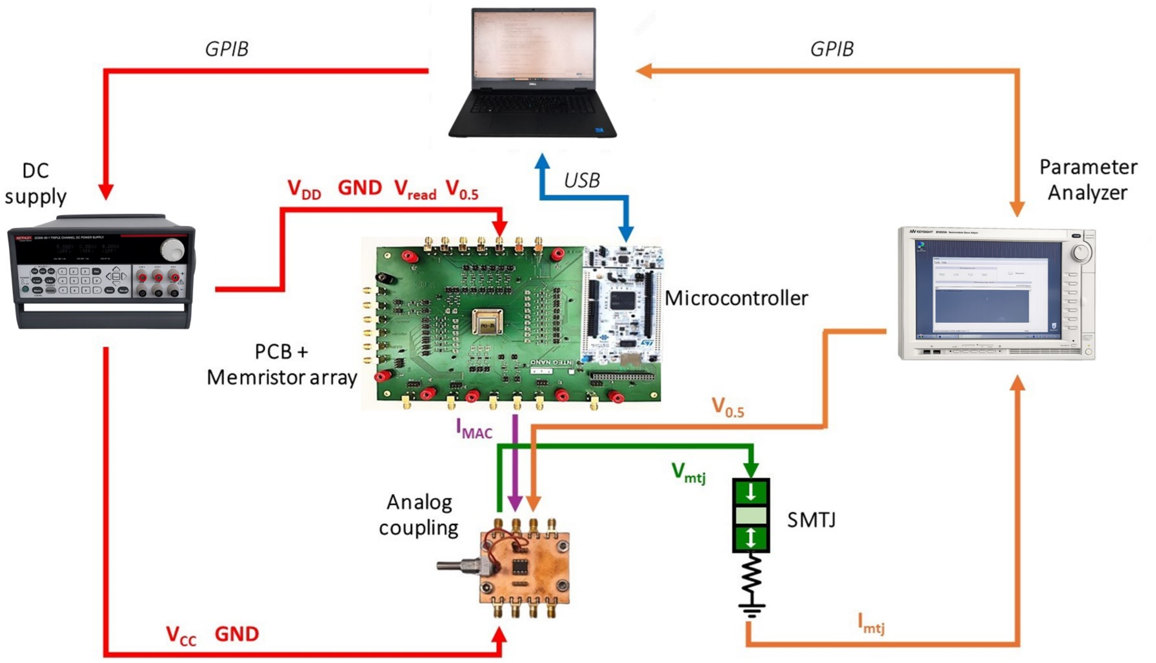

Supplementary Figure 5. Experimental configuration enabling analog coupling between the memristor array and the SMTJ. Measurement setup used for the experiment in Fig. 2. Computer-controlled instrumentation is used for pattern loading and data acquisition, while the coupling between the array output current and SMTJ bias is implemented entirely in analog hardware via an operational amplifier. The DC supply (Keithley 2230G) provides the array bias voltages (V_{DD} and V_{read}) and the reference level ($V_{0.5}$), while the parameter analyzer (Keysight B1530A) independently delivers an identical reference voltage ($V_{0.5}$) to the operational amplifier, and measures the magnetic tunnel junction (SMTJ) current (I_{mtj}). An MCP6002 operational amplifier, powered by an independent supply voltage (V_{CC}), converts the array output current (I_{MAC}) into the voltage (V_{mtj}) driving the SMTJ. An STM32 microcontroller loads the test patterns onto the array, and instrument control and data acquisition are automated through a Python script via GPIB and USB interfaces.

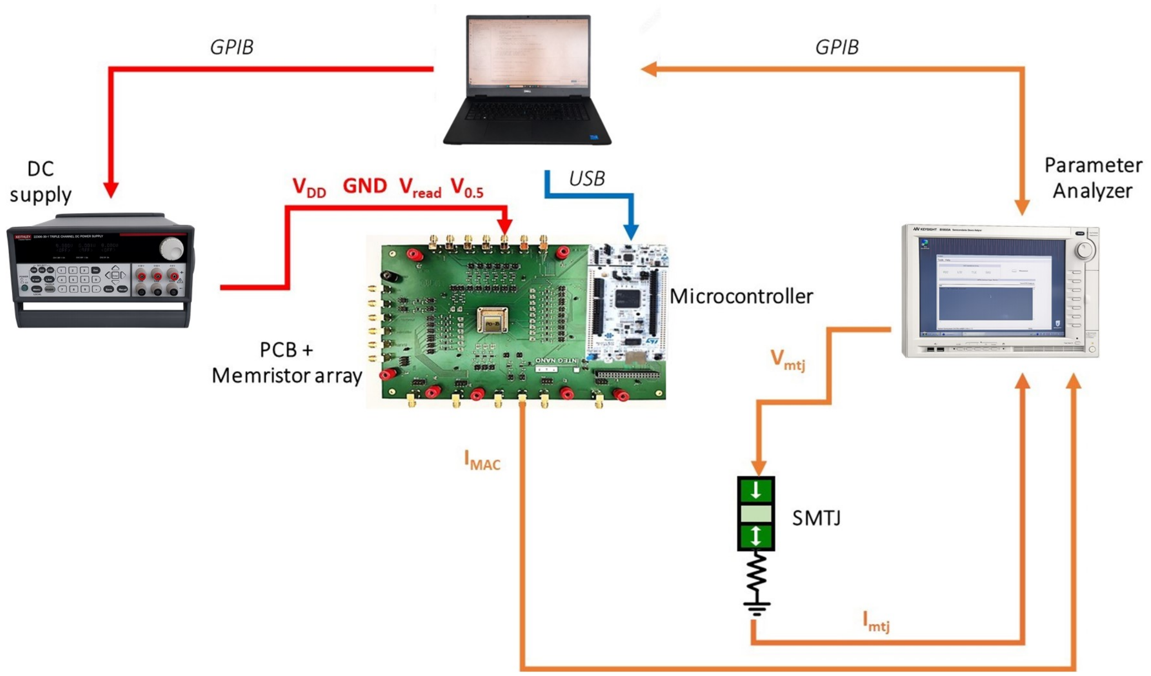

Supplementary Figure 6. Experimental configuration for computer-mediated coupling between the memristor array and the SMTJ. Computer-in-the-loop measurement setup used for the experiments in Figs. 4 and 5. The DC supply (Keithley 2230G) provides the array bias voltages (V_{DD} and V_{read}) and the reference level ($V_{0.5}$), while the parameter analyzer (Keysight B1530A) drives the magnetic tunnel junction (SMTJ) with (V_{mtj}) and measures I_{mtj}. The printed circuit board (PCB) hosts the memristor array and an STM32 microcontroller that loads the test patterns. The analog output current (I_{MAC}) from the array is sent to the B1530A, which forwards it to the control computer; the Python script then determines the corresponding V_{mtj} to be applied by the B1530A. Instrument communication and data acquisition are managed through GPIB and USB interfaces.

Supplementary Note 3: Benchmark tasks solved experimentally

This Note supplements Figs. 4 and 5 of the main article by presenting, in detail, the programmed coupling matrices used in the experimental resolution of the selected 24-node weighted MAX-CUT (MxC) and 10-vertex graph-coloring (GC) problem instances.

For the selected weighted MAX-CUT problem, all couplings are nominally negative. In this work, negative weights are implemented by reversing the polarity of the applied V_{read}. A differential encoding scheme that would enable the representation of both positive and negative weights is presented in Suppl. Note 6.

The experiment employs three discrete coupling levels: a unit weight corresponds to a conductance of 33 µS, while the higher levels are 66 µS and 99 µS. There is no bias column in the MAX-CUT formulation. The adjacency matrix is implemented by programming a 24×24 memristor array with the targeted conductance values (in µS) shown below. The hardware-agnostic form of the problem can be recovered by dividing this matrix by -33.

$$
MxC_{\mathrm{nominal}} = \begin{bmatrix}
0 & 0 & 0 & 99 & 0 & 0 & 0 & 0 & 0 & 0 & 0 & 0 & 0 & 0 & 0 & 0 & 99 & 0 & 0 & 0 & 0 & 0 & 0 & 0 \\
0 & 0 & 0 & 0 & 0 & 0 & 0 & 99 & 66 & 0 & 0 & 0 & 66 & 0 & 0 & 99 & 0 & 0 & 0 & 0 & 0 & 0 & 0 & 66 \\
0 & 0 & 0 & 0 & 0 & 99 & 0 & 0 & 0 & 0 & 0 & 0 & 0 & 0 & 0 & 66 & 99 & 33 & 0 & 0 & 0 & 0 & 0 & 0 \\
99 & 0 & 0 & 0 & 0 & 66 & 0 & 0 & 0 & 0 & 33 & 0 & 0 & 0 & 0 & 0 & 0 & 0 & 0 & 0 & 66 & 66 & 0 & 0 \\
0 & 0 & 0 & 0 & 0 & 0 & 0 & 0 & 66 & 0 & 0 & 0 & 99 & 0 & 0 & 0 & 0 & 33 & 0 & 0 & 0 & 0 & 0 & 0 \\
0 & 0 & 99 & 66 & 0 & 0 & 0 & 0 & 0 & 0 & 0 & 0 & 99 & 66 & 0 & 0 & 0 & 66 & 0 & 0 & 0 & 0 & 33 & 0 \\
0 & 0 & 0 & 0 & 0 & 0 & 0 & 0 & 0 & 0 & 0 & 0 & 0 & 0 & 0 & 0 & 0 & 0 & 0 & 0 & 0 & 0 & 66 & 0 \\
0 & 99 & 0 & 0 & 0 & 0 & 0 & 0 & 33 & 0 & 0 & 0 & 0 & 0 & 0 & 99 & 0 & 0 & 0 & 99 & 0 & 0 & 99 & 0 \\
0 & 66 & 0 & 0 & 66 & 0 & 0 & 33 & 0 & 0 & 0 & 0 & 0 & 0 & 99 & 0 & 0 & 0 & 0 & 0 & 0 & 0 & 0 & 0 \\
0 & 0 & 0 & 0 & 0 & 0 & 0 & 0 & 0 & 0 & 0 & 0 & 0 & 0 & 0 & 0 & 66 & 0 & 0 & 99 & 0 & 0 & 0 & 0 \\
0 & 0 & 0 & 33 & 0 & 0 & 0 & 0 & 0 & 0 & 0 & 99 & 0 & 0 & 0 & 0 & 0 & 99 & 0 & 0 & 0 & 0 & 0 & 0 \\
0 & 0 & 0 & 0 & 0 & 0 & 0 & 0 & 0 & 0 & 99 & 0 & 0 & 0 & 0 & 0 & 0 & 0 & 0 & 0 & 0 & 99 & 0 & 0 \\
0 & 66 & 0 & 0 & 99 & 99 & 0 & 0 & 0 & 0 & 0 & 0 & 0 & 0 & 0 & 99 & 0 & 0 & 0 & 0 & 0 & 0 & 0 & 33 \\
0 & 0 & 0 & 0 & 0 & 66 & 0 & 0 & 0 & 0 & 0 & 0 & 0 & 0 & 0 & 0 & 0 & 0 & 0 & 0 & 0 & 0 & 0 & 0 \\
0 & 0 & 0 & 0 & 0 & 0 & 0 & 0 & 99 & 0 & 0 & 0 & 0 & 0 & 0 & 0 & 0 & 66 & 0 & 0 & 0 & 0 & 0 & 0 \\
0 & 99 & 66 & 0 & 0 & 0 & 0 & 99 & 0 & 0 & 0 & 0 & 99 & 0 & 0 & 0 & 0 & 0 & 0 & 0 & 0 & 0 & 99 & 0 \\
99 & 0 & 99 & 0 & 0 & 0 & 0 & 0 & 0 & 66 & 0 & 0 & 0 & 0 & 0 & 0 & 0 & 66 & 0 & 0 & 0 & 99 & 0 & 0 \\
0 & 0 & 33 & 0 & 33 & 66 & 0 & 0 & 0 & 0 & 99 & 0 & 0 & 0 & 66 & 0 & 66 & 0 & 66 & 0 & 0 & 0 & 0 & 0 \\
0 & 0 & 0 & 0 & 0 & 0 & 0 & 0 & 0 & 0 & 0 & 0 & 0 & 0 & 0 & 0 & 0 & 66 & 0 & 99 & 0 & 0 & 66 & 0 \\
0 & 0 & 0 & 0 & 0 & 0 & 0 & 99 & 0 & 99 & 0 & 0 & 0 & 0 & 0 & 0 & 0 & 0 & 99 & 0 & 0 & 0 & 0 & 0 \\
0 & 0 & 0 & 66 & 0 & 0 & 0 & 0 & 0 & 0 & 0 & 0 & 0 & 0 & 0 & 0 & 0 & 0 & 0 & 0 & 0 & 0 & 0 & 0 \\
0 & 0 & 0 & 66 & 0 & 0 & 0 & 0 & 0 & 0 & 0 & 99 & 0 & 0 & 0 & 0 & 99 & 0 & 0 & 0 & 0 & 0 & 0 & 0 \\
0 & 0 & 0 & 0 & 0 & 33 & 66 & 99 & 0 & 0 & 0 & 0 & 0 & 0 & 0 & 99 & 0 & 0 & 66 & 0 & 0 & 0 & 0 & 0 \\
0 & 66 & 0 & 0 & 0 & 0 & 0 & 0 & 0 & 0 & 0 & 0 & 33 & 0 & 0 & 0 & 0 & 0 & 0 & 0 & 0 & 0 & 0 & 0
\end{bmatrix}
$$

After programming, the conductances obtained through the write–verify process deviate slightly from their nominal targets due to device variability. The resulting matrix below reports the measured conductance values of the 24×24 memristor array before running the experiment (values in µS, rounded to the first decimal place.)

$$
MxC_{\mathrm{exp}} = \begin{bmatrix}
0.1 & 0.4 & 0.5 & 100 & 0.4 & 0.4 & 0.3 & 0.5 & 0.5 & 0.2 & 0.4 & 1 & 3 & 0.4 & 0.3 & 0.4 & 100 & 0.4 & 0.2 & 0.1 & 0.1 & 0.4 & 0.5 & 0.4 \\
0.4 & 0.3 & 0.3 & 0.4 & 0.5 & 0.5 & 0.5 & 100 & 70 & 0.3 & 5 & 0.4 & 70 & 0.5 & 0.5 & 100 & 0.5 & 0.3 & 0.4 & 0.1 & 0.4 & 0.2 & 0.2 & 70 \\
0.4 & 0.4 & 0.3 & 0.5 & 0.4 & 99 & 0.1 & 2 & 0.4 & 0.6 & 0.4 & 0.4 & 0.4 & 0.3 & 0.4 & 70 & 100 & 40 & 0.5 & 0.4 & 0.2 & 0.5 & 0.1 & 0.4 \\
97 & 0.4 & 0.4 & 0.3 & 0.4 & 70 & 0.4 & 0.4 & 0.4 & 0.4 & 30 & 0.2 & 0.4 & 0.2 & 0.3 & 0.4 & 0.4 & 0.1 & 0.5 & 0.4 & 60 & 70 & 0.4 & 0.5 \\
0.5 & 0.4 & 0.4 & 0.4 & 0.4 & 0.3 & 0.4 & 0.1 & 70 & 0.4 & 0.4 & 0.4 & 100 & 0.4 & 0.3 & 0.4 & 0.4 & 30 & 0.3 & 7 & 0.4 & 0.3 & 0.4 & 0.5 \\
0.4 & 0.4 & 96 & 65 & 0.3 & 0.4 & 0.4 & 0.5 & 0.2 & 0.4 & 0.4 & 0.2 & 100 & 64 & 0.4 & 0.4 & 0.3 & 65 & 0.5 & 2 & 0.4 & 0.4 & 36 & 0.2 \\
0.4 & 0.4 & 0.4 & 0.4 & 0.4 & 0.4 & 0.3 & 0.4 & 0.5 & 0.5 & 0.4 & 0.4 & 0.4 & 0.4 & 0.4 & 0.4 & 10 & 0.4 & 0.4 & 0.4 & 0.3 & 0.4 & 62 & 0.4 \\
0.4 & 100 & 0.4 & 0.3 & 0.4 & 0.4 & 0.3 & 0.1 & 34 & 0.5 & 0.3 & 0.2 & 9 & 0.4 & 0.4 & 100 & 0.4 & 0.4 & 0.4 & 100 & 0.4 & 0.3 & 100 & 0.4 \\
0.4 & 70 & 0.2 & 0.2 & 65 & 0.4 & 0.3 & 34 & 0.3 & 8 & 0.4 & 0.5 & 0.2 & 0.5 & 100 & 0.4 & 0.9 & 0.4 & 3 & 0.1 & 0.4 & 7 & 0.4 & 0.4 \\
0.3 & 0.5 & 0.4 & 0.2 & 0.4 & 0.4 & 0.4 & 1 & 0.4 & 0.4 & 0.4 & 0.4 & 0.4 & 0.5 & 0.4 & 0.5 & 70 & 0.4 & 0.2 & 97 & 2 & 0.4 & 0.4 & 0.1 \\
0.5 & 0.4 & 0.4 & 34 & 0.1 & 0.4 & 0.3 & 0.4 & 0.4 & 0.5 & 0.4 & 100 & 0.4 & 0.4 & 0.4 & 0.5 & 0.4 & 100 & 0.4 & 0.4 & 0.4 & 0.1 & 0.4 & 0.4 \\
0.4 & 0.4 & 0.4 & 0.4 & 0.5 & 0.4 & 0.4 & 0.3 & 0.4 & 0.4 & 98 & 0.5 & 0.5 & 0.1 & 0.4 & 0.2 & 0.4 & 0.3 & 0.5 & 7 & 0.4 & 97 & 0.1 & 0.4 \\
0.4 & 64 & 0.5 & 0.4 & 100 & 99 & 8 & 1 & 0.3 & 0.4 & 0.4 & 0.4 & 0.3 & 0.3 & 0.4 & 100 & 4 & 0.4 & 0.3 & 0.1 & 0.4 & 0.4 & 10 & 32 \\
0.5 & 0.4 & 0.4 & 0.3 & 1 & 63 & 0.4 & 0.4 & 0.4 & 0.2 & 0.4 & 0.4 & 0.4 & 0.4 & 0.2 & 6 & 0.4 & 8 & 0.3 & 2 & 0.4 & 0.4 & 0.4 & 0.3 \\
0.6 & 0.1 & 0.4 & 0.5 & 0.1 & 0.1 & 0.4 & 0.5 & 99 & 0.3 & 0.5 & 0.4 & 0.4 & 0.5 & 0.4 & 0.4 & 0.4 & 65 & 0.4 & 0.4 & 0.2 & 0.3 & 0.4 & 0.4 \\
0.1 & 100 & 62 & 0.5 & 0.1 & 0.5 & 0.3 & 99 & 3 & 0.4 & 0.4 & 2 & 100 & 0.4 & 0.4 & 0.5 & 3 & 0.4 & 0.4 & 0.5 & 0.3 & 0.3 & 100 & 0.5 \\
100 & 0.5 & 97 & 0.3 & 4 & 0.4 & 0.4 & 0.2 & 0.4 & 63 & 0.4 & 0.3 & 0.4 & 0.4 & 0.1 & 0.2 & 0.4 & 67 & 0.4 & 0.3 & 0.3 & 100 & 0.3 & 0.4 \\
0.3 & 0.3 & 37 & 0.3 & 38 & 58 & 0.6 & 0.3 & 0.2 & 0.5 & 98 & 0.5 & 0.4 & 0.4 & 68 & 0.3 & 67 & 0.4 & 68 & 0.4 & 0.4 & 0.4 & 0.4 & 1 \\
0.4 & 0.4 & 0.4 & 0.4 & 0.5 & 0.4 & 8 & 0.5 & 0.4 & 0.5 & 0.5 & 0.4 & 0.4 & 0.2 & 0.5 & 0.4 & 0.3 & 65 & 0.5 & 100 & 0.4 & 0.3 & 69 & 0.4 \\
0.5 & 0.4 & 0.4 & 0.3 & 0.4 & 0.4 & 0.5 & 100 & 0.4 & 98 & 0.4 & 3 & 0.4 & 0.4 & 0.3 & 0.5 & 0.5 & 0.4 & 97 & 0.4 & 0.5 & 0.4 & 0.3 & 8 \\
0.4 & 0.2 & 0.5 & 64 & 0.4 & 0.5 & 0.4 & 0.4 & 0.5 & 0.4 & 0.2 & 0.4 & 0.4 & 0.5 & 0.1 & 0.1 & 0.2 & 0.1 & 0.5 & 0.5 & 0.1 & 0.4 & 0.3 & 0.3 \\
0.4 & 0.4 & 0.4 & 63 & 0.3 & 0.3 & 0.3 & 0.4 & 0.5 & 0.4 & 0.9 & 100 & 4 & 0.3 & 0.4 & 0.3 & 100 & 0.4 & 0.2 & 0.3 & 0.5 & 0.5 & 0.3 & 0.3 \\
0.4 & 0.4 & 0.3 & 0.4 & 0.4 & 37 & 67 & 98 & 0.3 & 0.5 & 0.2 & 0.4 & 0.4 & 0.4 & 0.4 & 97 & 0.4 & 0.4 & 65 & 0.4 & 0.4 & 0.5 & 0.4 & 0.4 \\
0.4 & 63 & 0.4 & 0.4 & 0.4 & 0.1 & 0.4 & 0.4 & 0.4 & 0.4 & 0.4 & 0.3 & 36 & 0.4 & 0.5 & 0.4 & 0.5 & 0.3 & 0.4 & 0.4 & 0.4 & 0.4 & 0.4 & 0.8
\end{bmatrix}
$$

For the graph-coloring (GC) instance, we consider 10 vertices and 3 colors with a one-hot encoding, yielding 30 binary variables. The couplings are arranged as a 30×30 matrix J with a bias column vector $\mathbf{h}$ physically appended, forming the augmented block $[\, J \mid \mathbf{h} \,] \in \mathbb{R}^{30\times 31}$. The signs are homogeneous within J and within $\mathbf{h}$ (matrix entries negative, bias entries positive), and are differentiated via the sign of the applied V_{read}. Only two coupling levels are required: the unit weight corresponds to 70 µS, and the double weight to 140 µS. The associated matrix of targeted conductance values is shown below; the hardware-agnostic representation of the problem is obtained by dividing J by -70 and h by 70.

$$
GC_{\text{nominal}} = \left[\begin{array}{cccccccccccccccccccccccccccccc|c}
0 & 140 & 140 & 140 & 0 & 0 & 0 & 0 & 0 & 0 & 0 & 0 & 140 & 0 & 0 & 0 & 0 & 0 & 0 & 0 & 0 & 140 & 0 & 0 & 140 & 0 & 0 & 140 & 0 & 0 & 70 \\
140 & 0 & 140 & 0 & 140 & 0 & 0 & 0 & 0 & 0 & 0 & 0 & 0 & 140 & 0 & 0 & 0 & 0 & 0 & 0 & 0 & 0 & 140 & 0 & 0 & 140 & 0 & 0 & 140 & 0 & 70 \\
140 & 140 & 0 & 0 & 0 & 140 & 0 & 0 & 0 & 0 & 0 & 0 & 0 & 0 & 140 & 0 & 0 & 0 & 0 & 0 & 0 & 0 & 0 & 140 & 0 & 0 & 140 & 0 & 0 & 140 & 70 \\
140 & 0 & 0 & 0 & 140 & 140 & 140 & 0 & 0 & 0 & 0 & 0 & 140 & 0 & 0 & 0 & 0 & 0 & 0 & 0 & 0 & 0 & 0 & 0 & 0 & 0 & 0 & 0 & 0 & 0 & 70 \\
0 & 140 & 0 & 140 & 0 & 140 & 0 & 140 & 0 & 0 & 0 & 0 & 0 & 140 & 0 & 0 & 0 & 0 & 0 & 0 & 0 & 0 & 0 & 0 & 0 & 0 & 0 & 0 & 0 & 0 & 70 \\
0 & 0 & 140 & 140 & 140 & 0 & 0 & 0 & 140 & 0 & 0 & 0 & 0 & 0 & 140 & 0 & 0 & 0 & 0 & 0 & 0 & 0 & 0 & 0 & 0 & 0 & 0 & 0 & 0 & 0 & 70 \\
0 & 0 & 0 & 140 & 0 & 0 & 0 & 140 & 140 & 0 & 0 & 0 & 140 & 0 & 0 & 140 & 0 & 0 & 0 & 0 & 0 & 140 & 0 & 0 & 140 & 0 & 0 & 140 & 0 & 0 & 70 \\
0 & 0 & 0 & 0 & 140 & 0 & 140 & 0 & 140 & 0 & 0 & 0 & 0 & 140 & 0 & 0 & 140 & 0 & 0 & 0 & 0 & 0 & 140 & 0 & 0 & 140 & 0 & 0 & 140 & 0 & 70 \\
0 & 0 & 0 & 0 & 0 & 140 & 140 & 140 & 0 & 0 & 0 & 0 & 0 & 0 & 140 & 0 & 0 & 140 & 0 & 0 & 0 & 0 & 0 & 140 & 0 & 0 & 140 & 0 & 0 & 140 & 70 \\
0 & 0 & 0 & 0 & 0 & 0 & 0 & 0 & 0 & 0 & 140 & 140 & 0 & 0 & 0 & 0 & 0 & 0 & 140 & 0 & 0 & 0 & 0 & 0 & 140 & 0 & 0 & 0 & 0 & 0 & 70 \\
0 & 0 & 0 & 0 & 0 & 0 & 0 & 0 & 0 & 140 & 0 & 140 & 0 & 0 & 0 & 0 & 0 & 0 & 0 & 140 & 0 & 0 & 0 & 0 & 0 & 140 & 0 & 0 & 0 & 0 & 70 \\
0 & 0 & 0 & 0 & 0 & 0 & 0 & 0 & 0 & 140 & 140 & 0 & 0 & 0 & 0 & 0 & 0 & 0 & 0 & 0 & 140 & 0 & 0 & 0 & 0 & 0 & 140 & 0 & 0 & 0 & 70 \\
140 & 0 & 0 & 140 & 0 & 0 & 140 & 0 & 0 & 0 & 0 & 0 & 0 & 140 & 140 & 0 & 0 & 0 & 0 & 0 & 0 & 0 & 0 & 0 & 0 & 0 & 0 & 0 & 0 & 0 & 70 \\
0 & 140 & 0 & 0 & 140 & 0 & 0 & 140 & 0 & 0 & 0 & 0 & 140 & 0 & 140 & 0 & 0 & 0 & 0 & 0 & 0 & 0 & 0 & 0 & 0 & 0 & 0 & 0 & 0 & 0 & 70 \\
0 & 0 & 140 & 0 & 0 & 140 & 0 & 0 & 140 & 0 & 0 & 0 & 140 & 140 & 0 & 0 & 0 & 0 & 0 & 0 & 0 & 0 & 0 & 0 & 0 & 0 & 0 & 0 & 0 & 0 & 70 \\
0 & 0 & 0 & 0 & 0 & 0 & 140 & 0 & 0 & 0 & 0 & 0 & 0 & 0 & 0 & 0 & 140 & 140 & 0 & 0 & 0 & 0 & 0 & 0 & 140 & 0 & 0 & 0 & 0 & 0 & 70 \\
0 & 0 & 0 & 0 & 0 & 0 & 0 & 140 & 0 & 0 & 0 & 0 & 0 & 0 & 0 & 140 & 0 & 140 & 0 & 0 & 0 & 0 & 0 & 0 & 0 & 140 & 0 & 0 & 0 & 0 & 70 \\
0 & 0 & 0 & 0 & 0 & 0 & 0 & 0 & 140 & 0 & 0 & 0 & 0 & 0 & 0 & 140 & 140 & 0 & 0 & 0 & 0 & 0 & 0 & 0 & 0 & 0 & 140 & 0 & 0 & 0 & 70 \\
0 & 0 & 0 & 0 & 0 & 0 & 0 & 0 & 0 & 140 & 0 & 0 & 0 & 0 & 0 & 0 & 0 & 0 & 0 & 140 & 140 & 0 & 0 & 0 & 0 & 0 & 0 & 140 & 0 & 0 & 70 \\
0 & 0 & 0 & 0 & 0 & 0 & 0 & 0 & 0 & 0 & 140 & 0 & 0 & 0 & 0 & 0 & 0 & 0 & 140 & 0 & 140 & 0 & 0 & 0 & 0 & 0 & 0 & 0 & 140 & 0 & 70 \\
0 & 0 & 0 & 0 & 0 & 0 & 0 & 0 & 0 & 0 & 0 & 140 & 0 & 0 & 0 & 0 & 0 & 0 & 140 & 140 & 0 & 0 & 0 & 0 & 0 & 0 & 0 & 0 & 0 & 140 & 70 \\
140 & 0 & 0 & 0 & 0 & 0 & 140 & 0 & 0 & 0 & 0 & 0 & 0 & 0 & 0 & 0 & 0 & 0 & 0 & 0 & 0 & 0 & 140 & 140 & 0 & 0 & 0 & 140 & 0 & 0 & 70 \\
0 & 140 & 0 & 0 & 0 & 0 & 0 & 140 & 0 & 0 & 0 & 0 & 0 & 0 & 0 & 0 & 0 & 0 & 0 & 0 & 0 & 140 & 0 & 140 & 0 & 0 & 0 & 0 & 140 & 0 & 70 \\
0 & 0 & 140 & 0 & 0 & 0 & 0 & 0 & 140 & 0 & 0 & 0 & 0 & 0 & 0 & 0 & 0 & 0 & 0 & 0 & 0 & 140 & 140 & 0 & 0 & 0 & 0 & 0 & 0 & 140 & 70 \\
140 & 0 & 0 & 0 & 0 & 0 & 140 & 0 & 0 & 140 & 0 & 0 & 0 & 0 & 0 & 140 & 0 & 0 & 0 & 0 & 0 & 0 & 0 & 0 & 0 & 140 & 140 & 0 & 0 & 0 & 70 \\
0 & 140 & 0 & 0 & 0 & 0 & 0 & 140 & 0 & 0 & 140 & 0 & 0 & 0 & 0 & 0 & 140 & 0 & 0 & 0 & 0 & 0 & 0 & 0 & 140 & 0 & 140 & 0 & 0 & 0 & 70 \\
0 & 0 & 140 & 0 & 0 & 0 & 0 & 0 & 140 & 0 & 0 & 140 & 0 & 0 & 0 & 0 & 0 & 140 & 0 & 0 & 0 & 0 & 0 & 0 & 140 & 140 & 0 & 0 & 0 & 0 & 70 \\
140 & 0 & 0 & 0 & 0 & 0 & 140 & 0 & 0 & 0 & 0 & 0 & 0 & 0 & 0 & 0 & 0 & 0 & 140 & 0 & 0 & 140 & 0 & 0 & 0 & 0 & 0 & 0 & 140 & 140 & 70 \\
0 & 140 & 0 & 0 & 0 & 0 & 0 & 140 & 0 & 0 & 0 & 0 & 0 & 0 & 0 & 0 & 0 & 0 & 0 & 140 & 0 & 0 & 140 & 0 & 0 & 0 & 0 & 140 & 0 & 140 & 70 \\
0 & 0 & 140 & 0 & 0 & 0 & 0 & 0 & 140 & 0 & 0 & 0 & 0 & 0 & 0 & 0 & 0 & 0 & 0 & 0 & 140 & 0 & 0 & 140 & 0 & 0 & 0 & 140 & 140 & 0 & 70
\end{array}\right]
$$

As in the MAX-CUT case, the actual conductances obtained after programming differ slightly from the targeted values due to device variability. The measured conductance matrix of the 30×31 array is shown below.

$GC_{\text{exp}} =$

$$
\left[\begin{array}{cccccccccccccccccccccccccccccc|c}
0.8 & 142.6 & 143.4 & 144.5 & 0.7 & 9.8 & 0.7 & 0.6 & 0.8 & 0.7 & 0.6 & 0.7 & 142.0 & 0.3 & 0.7 & 0.7 & 0.6 & 0.6 & 0.6 & 0.5 & 0.6 & 145.0 & 0.2 & 0.8 & 145.7 & 0.6 & 0.7 & 139.6 & 0.1 & 0.7 & 69.6 \\
141.8 & 0.7 & 144.2 & 0.9 & 141.5 & 0.7 & 0.5 & 0.8 & 0.6 & 0.7 & 0.7 & 0.6 & 0.7 & 145.9 & 0.6 & 0.6 & 0.6 & 0.6 & 0.7 & 0.8 & 0.6 & 0.6 & 143.9 & 0.7 & 1.1 & 146.3 & 0.2 & 0.6 & 141.4 & 0.3 & 75.8 \\
145.3 & 142.0 & 0.6 & 0.6 & 0.7 & 141.9 & 0.6 & 0.7 & 0.6 & 0.2 & 0.5 & 0.6 & 0.6 & 0.6 & 139.9 & 0.7 & 0.7 & 0.8 & 0.7 & 0.7 & 0.7 & 0.7 & 0.6 & 145.6 & 0.7 & 0.7 & 148.1 & 0.6 & 0.6 & 142.7 & 77.9 \\
138.6 & 0.7 & 0.7 & 2.4 & 142.6 & 136.1 & 147.1 & 0.6 & 0.6 & 0.6 & 0.5 & 0.5 & 146.8 & 1.1 & 0.7 & 0.7 & 0.7 & 0.6 & 0.7 & 0.7 & 0.1 & 5.5 & 0.7 & 0.7 & 0.7 & 0.6 & 0.7 & 0.7 & 0.6 & 0.7 & 75.6 \\
1.0 & 142.4 & 0.6 & 143.1 & 0.6 & 149.4 & 0.6 & 133.6 & 0.7 & 0.7 & 0.4 & 0.7 & 0.8 & 144.3 & 0.7 & 0.7 & 0.7 & 5.5 & 0.8 & 0.7 & 8.1 & 0.7 & 0.8 & 0.7 & 0.8 & 0.7 & 0.7 & 0.7 & 0.7 & 0.4 & 72.1 \\
0.6 & 0.4 & 140.8 & 140.2 & 146.0 & 0.6 & 0.6 & 0.7 & 137.7 & 0.6 & 0.7 & 0.7 & 0.6 & 0.7 & 144.9 & 0.6 & 0.6 & 0.7 & 0.6 & 0.7 & 0.7 & 0.7 & 0.6 & 0.5 & 0.2 & 0.6 & 0.6 & 0.6 & 0.2 & 0.5 & 70.3 \\
0.6 & 0.2 & 0.6 & 141.5 & 0.1 & 0.6 & 0.7 & 142.3 & 143.1 & 0.7 & 0.7 & 0.6 & 142.3 & 0.6 & 0.6 & 140.1 & 0.5 & 0.6 & 0.6 & 0.7 & 0.4 & 139.0 & 0.6 & 0.7 & 139.9 & 0.4 & 0.6 & 139.7 & 0.7 & 0.7 & 67.0 \\
0.6 & 0.6 & 0.6 & 4.8 & 140.1 & 0.6 & 143.6 & 0.7 & 143.6 & 0.7 & 0.6 & 0.7 & 0.8 & 144.0 & 0.4 & 0.7 & 143.6 & 0.6 & 0.6 & 0.6 & 0.6 & 0.5 & 140.1 & 0.5 & 0.6 & 139.0 & 0.7 & 0.6 & 142.2 & 0.7 & 73.3 \\
0.7 & 0.5 & 0.6 & 0.6 & 0.7 & 143.5 & 138.8 & 140.0 & 0.6 & 0.7 & 0.5 & 0.7 & 0.7 & 3.9 & 141.5 & 0.4 & 0.6 & 136.3 & 0.5 & 0.5 & 0.4 & 0.8 & 0.8 & 136.9 & 0.7 & 0.6 & 138.5 & 0.7 & 0.6 & 144.0 & 72.5 \\
0.5 & 0.6 & 0.7 & 0.6 & 0.7 & 0.6 & 0.2 & 0.6 & 0.7 & 0.7 & 145.1 & 138.4 & 0.6 & 0.6 & 0.5 & 0.6 & 0.6 & 0.6 & 138.3 & 0.6 & 0.6 & 0.7 & 0.6 & 0.7 & 137.6 & 0.7 & 0.6 & 0.7 & 0.6 & 0.7 & 73.4 \\
0.7 & 0.7 & 0.6 & 7.3 & 0.7 & 0.6 & 0.6 & 0.6 & 0.6 & 140.1 & 0.6 & 144.2 & 0.5 & 0.5 & 0.6 & 0.6 & 0.7 & 2.3 & 0.6 & 142.1 & 0.6 & 0.7 & 0.7 & 0.7 & 0.6 & 138.9 & 8.0 & 0.7 & 0.7 & 0.6 & 77.2 \\
0.4 & 0.7 & 0.6 & 0.7 & 0.1 & 0.5 & 0.6 & 0.6 & 0.6 & 141.5 & 145.3 & 0.7 & 0.6 & 0.7 & 0.6 & 0.5 & 0.8 & 0.6 & 0.7 & 0.5 & 140.6 & 0.6 & 0.6 & 0.1 & 0.7 & 0.7 & 141.2 & 0.6 & 0.7 & 0.6 & 68.1 \\
141.8 & 0.6 & 0.6 & 135.4 & 0.1 & 0.4 & 138.6 & 1.0 & 0.7 & 0.6 & 0.7 & 0.8 & 0.8 & 144.5 & 141.0 & 0.6 & 0.7 & 0.7 & 0.7 & 0.7 & 0.5 & 0.2 & 0.5 & 0.4 & 0.6 & 0.5 & 0.7 & 0.3 & 0.6 & 0.6 & 72.7 \\
0.6 & 140.2 & 0.7 & 0.7 & 140.3 & 0.7 & 0.7 & 142.7 & 0.6 & 1.4 & 0.6 & 0.6 & 142.1 & 0.6 & 141.4 & 0.7 & 0.7 & 0.6 & 0.6 & 0.6 & 0.5 & 0.6 & 0.6 & 0.6 & 0.5 & 0.7 & 0.6 & 0.1 & 0.3 & 0.7 & 69.9 \\
0.7 & 0.5 & 140.5 & 0.7 & 0.6 & 141.0 & 0.6 & 0.2 & 140.3 & 0.8 & 0.1 & 0.2 & 138.2 & 136.6 & 0.6 & 0.7 & 0.4 & 0.7 & 6.3 & 0.1 & 0.7 & 0.6 & 0.6 & 0.7 & 0.7 & 0.7 & 0.7 & 0.7 & 0.7 & 0.6 & 75.7 \\
0.7 & 0.6 & 0.3 & 0.6 & 0.6 & 0.7 & 141.1 & 0.6 & 0.6 & 0.6 & 0.6 & 1.9 & 0.5 & 0.6 & 0.7 & 0.7 & 135.2 & 138.2 & 0.7 & 0.8 & 0.6 & 0.7 & 7.3 & 0.7 & 138.9 & 0.5 & 0.6 & 0.7 & 0.6 & 0.1 & 73.2 \\
0.6 & 0.7 & 0.4 & 0.1 & 0.4 & 0.6 & 0.6 & 136.5 & 0.6 & 0.7 & 0.7 & 0.7 & 0.7 & 0.7 & 0.8 & 142.5 & 0.7 & 141.7 & 0.2 & 0.4 & 0.6 & 0.7 & 0.7 & 0.3 & 0.7 & 144.7 & 0.7 & 0.6 & 1.0 & 0.6 & 68.3 \\
0.6 & 0.6 & 0.6 & 0.7 & 0.6 & 0.5 & 0.7 & 0.7 & 138.9 & 0.1 & 0.7 & 0.7 & 0.2 & 3.2 & 0.5 & 139.2 & 139.0 & 0.5 & 0.7 & 0.7 & 0.7 & 0.9 & 0.3 & 0.6 & 0.6 & 0.7 & 145.3 & 0.6 & 0.6 & 0.3 & 76.1 \\
0.3 & 0.6 & 0.7 & 0.7 & 0.6 & 0.4 & 0.6 & 0.6 & 0.7 & 137.9 & 0.7 & 0.8 & 0.6 & 0.7 & 0.5 & 0.7 & 0.3 & 0.4 & 0.7 & 137.6 & 139.3 & 0.6 & 0.4 & 0.6 & 0.6 & 0.6 & 0.5 & 139.0 & 0.6 & 0.6 & 72.0 \\
0.7 & 0.7 & 0.6 & 5.7 & 0.7 & 0.6 & 0.6 & 0.7 & 0.6 & 0.6 & 141.2 & 0.7 & 0.7 & 0.7 & 0.6 & 0.7 & 0.7 & 0.6 & 134.7 & 0.7 & 141.7 & 0.5 & 0.6 & 0.4 & 0.5 & 0.5 & 0.6 & 0.7 & 140.6 & 0.6 & 76.9 \\
0.7 & 0.6 & 0.7 & 0.5 & 0.7 & 0.6 & 0.7 & 0.7 & 0.6 & 0.6 & 0.7 & 137.1 & 0.6 & 0.6 & 0.6 & 0.6 & 0.7 & 0.6 & 143.6 & 144.3 & 0.8 & 0.7 & 0.6 & 0.7 & 0.7 & 7.4 & 0.6 & 0.6 & 1.5 & 141.7 & 72.6 \\
141.5 & 0.5 & 0.6 & 0.5 & 0.6 & 0.7 & 136.6 & 0.7 & 0.5 & 0.5 & 0.6 & 0.6 & 0.3 & 0.7 & 0.7 & 0.7 & 0.7 & 0.4 & 0.7 & 0.7 & 0.3 & 0.7 & 136.2 & 141.4 & 0.7 & 0.7 & 0.7 & 147.4 & 0.8 & 0.7 & 76.1 \\
0.8 & 142.9 & 0.7 & 0.3 & 0.7 & 0.6 & 0.6 & 136.2 & 0.6 & 0.8 & 0.8 & 0.4 & 0.7 & 0.5 & 0.6 & 0.7 & 0.8 & 0.6 & 0.7 & 0.7 & 0.7 & 144.6 & 0.7 & 139.9 & 0.8 & 0.8 & 0.7 & 0.6 & 140.2 & 0.6 & 71.0 \\
0.3 & 0.2 & 145.2 & 0.6 & 0.4 & 0.7 & 0.6 & 0.7 & 138.6 & 0.6 & 0.7 & 0.7 & 0.7 & 0.7 & 0.3 & 0.7 & 0.6 & 0.7 & 0.7 & 0.5 & 0.7 & 143.6 & 139.0 & 0.7 & 0.7 & 0.7 & 0.6 & 0.5 & 0.7 & 140.1 & 68.8 \\
139.9 & 0.5 & 0.6 & 0.6 & 0.7 & 0.7 & 141.2 & 0.6 & 0.7 & 136.3 & 0.6 & 0.2 & 0.6 & 0.6 & 0.7 & 146.3 & 0.7 & 0.6 & 0.7 & 0.4 & 0.7 & 0.9 & 0.6 & 0.6 & 0.6 & 137.7 & 139.9 & 0.7 & 0.6 & 6.9 & 72.3 \\
0.7 & 136.2 & 0.5 & 0.7 & 0.8 & 5.1 & 0.7 & 137.6 & 0.7 & 0.7 & 152.0 & 0.7 & 0.7 & 0.6 & 0.8 & 0.7 & 147.5 & 5.1 & 0.6 & 0.6 & 0.6 & 0.1 & 0.7 & 0.7 & 147.2 & 0.7 & 140.4 & 0.7 & 0.6 & 0.7 & 72.8 \\
0.7 & 0.6 & 136.0 & 0.7 & 0.7 & 0.4 & 0.7 & 0.7 & 143.0 & 0.7 & 4.6 & 137.4 & 0.6 & 0.7 & 0.6 & 6.6 & 0.2 & 141.7 & 0.6 & 0.6 & 0.6 & 0.6 & 0.7 & 0.7 & 141.5 & 136.8 & 0.7 & 0.2 & 0.7 & 0.7 & 73.4 \\
138.6 & 0.7 & 0.7 & 0.7 & 0.6 & 0.8 & 138.3 & 0.7 & 0.6 & 0.6 & 0.7 & 0.5 & 0.6 & 0.6 & 0.2 & 0.6 & 0.7 & 0.7 & 137.5 & 0.6 & 0.8 & 135.4 & 0.4 & 0.3 & 0.6 & 0.7 & 0.6 & 0.6 & 146.1 & 146.0 & 75.1 \\
0.6 & 146.0 & 0.6 & 1.1 & 0.6 & 0.5 & 0.7 & 140.8 & 0.4 & 0.5 & 0.4 & 0.6 & 0.6 & 1.7 & 0.4 & 0.6 & 0.7 & 0.6 & 0.1 & 137.2 & 0.7 & 0.8 & 142.2 & 0.7 & 0.6 & 0.7 & 0.6 & 147.4 & 0.7 & 145.1 & 69.5 \\
0.7 & 0.7 & 142.1 & 0.7 & 0.6 & 0.6 & 0.6 & 0.6 & 135.0 & 0.4 & 0.7 & 0.7 & 0.7 & 0.6 & 0.8 & 0.7 & 0.7 & 0.7 & 0.7 & 0.8 & 144.9 & 0.7 & 0.8 & 143.7 & 0.5 & 0.6 & 8.5 & 143.3 & 147.0 & 0.6 & 67.0
\end{array}\right]
$$

Supplementary Note 4: Signal-to-noise ratio of memristor in-memory computation

This Note examines how the read voltage V_{read} influences the multiply-and-accumulate (MAC) operation signal-to-noise ratio (SNR) in our memristor array. Suppl. Fig. 7a shows the measured MAC currents plotted against their expected values for different V_{read}. The curves fall on the same line, indicating that the mean MAC response does not change appreciably with the read voltage in the range tested.

To evaluate noise, we grouped measurements corresponding to the same input-conductance combinations and extracted, for each group, the ratio μ/σ of the mean MAC current to its standard deviation. We use this quantity as an effective SNR for each configuration. As shown in Suppl. Fig. 7b, μ/σ varies systematically with the ideal MAC value and depends on V_{read}.

Suppl. Fig. 7c reports the average μ/σ for each V_{read}, obtained by averaging across all input-conductance combinations. This provides a compact view of how the effective SNR evolves with the read voltage, i.e., it improves with increasing V_{read}.

This trend is consistent with the way V_{read} is used to set the pseudo-temperature in our simulated annealing scheme. At high pseudo-temperature (low V_{read}), the lower SNR introduces greater stochasticity in the MAC outputs, which is compatible with the exploratory phase of the annealing process. As the pseudo-temperature is reduced (higher V_{read}), the SNR increases and the influence of noise becomes comparatively smaller, matching the need for more deterministic updates in the later stages. In this sense, the analog in-memory computation noise characteristics follow the intended temperature schedule rather than conflicting with it.

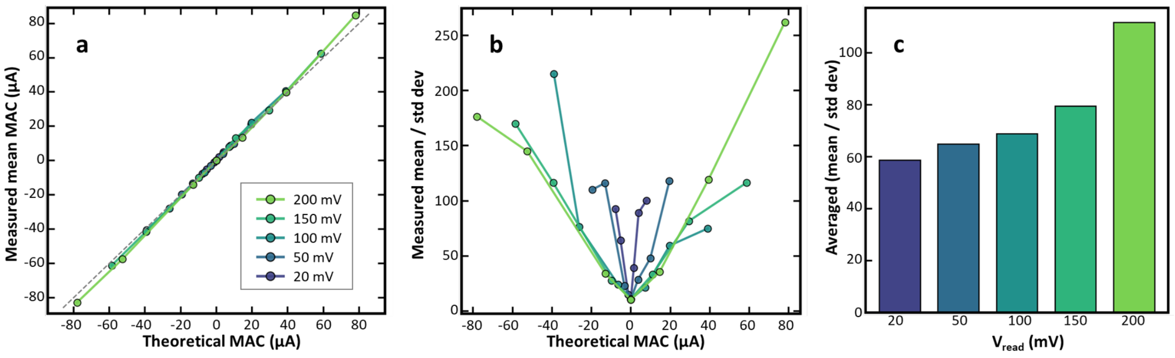

Supplementary Figure 7. Read-voltage dependence of MAC accuracy and noise. **a** Measured multiply-accumulate (MAC) currents compared with their ideal values for several read voltages V_{read}. **b** Effective SNR (μ/σ) extracted for each input-conductance combination and plotted against the corresponding ideal MAC value, illustrating how signal quality varies both with MAC magnitude and with V_{read}. **c** Average μ/σ for each V_{read}, providing a summary of the overall SNR behaviour as the read voltage is varied.

Supplementary Note 5: Stability of stochastic magnetic tunnel junction

This Note reports additional characterization of the stochastic magnetic tunnel junction (SMTJ) used in our experiments. To evaluate the temporal stability of the stochastic magnetic tunnel junction (SMTJ), we repeated the measurement of its antiparallel (AP) state probability after a waiting period of five days. During each sweep, the device was biased stepwise across the voltage range where transitions between magnetic states occur. At every bias point, the resistance was sampled at least 2 000 times while the voltage was held constant, and the fraction of high-resistance (AP) readings was taken as the probability of the device being in the AP state. The resulting probability curves were fitted with sigmoidal functions to extract the equiprobable bias point.

Two measurements were performed on the same device: one used as a reference ($t = 0$) and another after five days under identical conditions. Suppl. Fig. 8 shows the corresponding curves, with red squares indicating the reference measurement and blue circles the measurement after five days, together with dashed lines showing the respective sigmoidal fits.

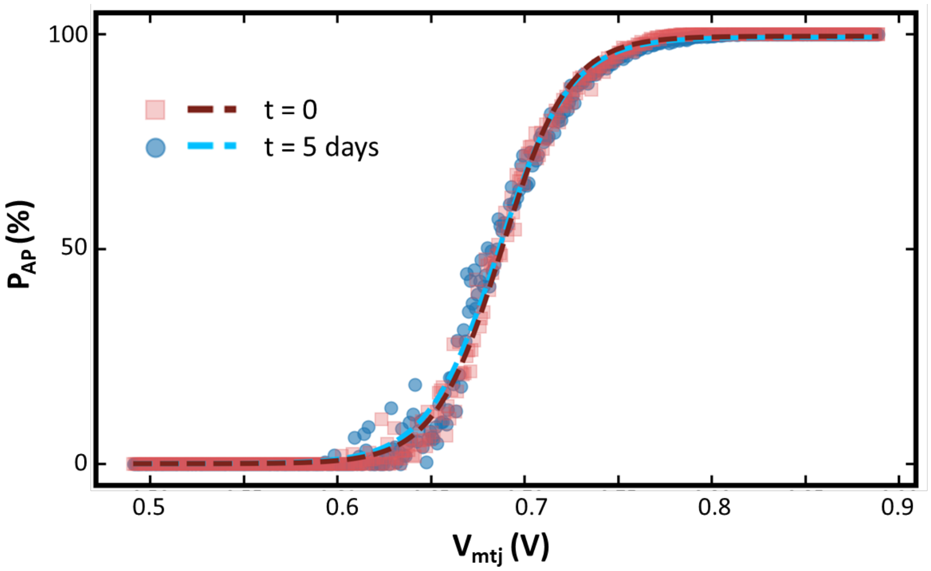

Supplementary Figure 8. SMTJ drift experiment. Switching probability of the SMTJ as a function of applied voltage (V_{mtj}). Red squares correspond to measurements at $t = 0$, while blue circles correspond to measurements after 5 days. Both datasets are fitted with sigmoidal functions (dark-red and cyan dashed lines), showing a negligible shift (< 2 mV) in the interpolated equiprobable bias point over time.

Supplementary Note 6: Design and device assumptions for projections

This Supplementary Note outlines architectural extensions of our hybrid memristor–SMTJ Ising machine that (i) generalize the encoding of signed couplings, (ii) integrate a flexible hardware annealing schedule, (iii) enable parallel spin updates, and (iv) increase the sampling throughput by exploiting fast stochastic MTJs. These considerations underpin the projected system-level performance addressed in the Discussion of the main text.

Sign control of weights and spins

In the present prototype, each coupling is stored in a single memristor, and its sign is imposed at the column level during the MAC operation by selecting the polarity of V_{read}. A natural extension for future chips is to use a differential pair (g^+, g^-) per coupling, so that the effective weight is given by their difference

$$J_{ij} \propto g_{ij}^+ - g_{ij}^-$$

This scheme provides full per-weight sign control and improves robustness to slow drifts that are common to both devices (e.g., temperature-dependent shifts), as these are largely rejected in the differential operation.

The spin variables must be supplied dynamically, since they are updated at every sampling step. Updated spin states can be latched into a small register bank that drives the column-voltage or routing selection. Several encoding options are available:

- *Ising encoding $s_j \in \{\pm 1\}$ with signed column voltages.* Each column driver directly applies $V_j = s_j V_{\text{read}}$, selecting between $+V_{\text{read}}$ and $-V_{\text{read}}$. The weight sign is then set by (g^+, g^-), and no additional per-cell routing is required.

- *Ising encoding with local routing.* All column voltages remain positive, and a small local switch routes either g^+ or g^- to the row summation line depending on s_j. This keeps the column drivers single-ended but introduces per-cell routing overhead.

- *QUBO encoding $x_j \in \{0, 1\}$.* Spins are encoded as x_j, and the problem is mapped via $s_j = 2x_j - 1$. Columns are either driven at V_{read} (for $x_j = 1$) or disconnected/held at reference (for $x_j = 0$), while the sign of the coupling is entirely carried by the differential pair (g^+, g^-).

All three options are compatible with the MAC operation; the choice depends primarily on circuit complexity, routing constraints, and whether the algorithm is formulated directly in Ising form or in QUBO form.

Annealing schedule and update control

In our architecture, the effective inverse pseudo-temperature β is implemented as a global read amplitude V_{read} applied to the columns. A convenient hardware realization is to represent β as a k-bit digital word and convert it to V_{read} using an on-chip R–2R ladder DAC. Each bit controls a switch associated with a binary-weighted branch of the ladder; with k stages, the DAC generates 2^k levels between 0 and

a maximum value $V_{\text{read}}^{\text{max}}$, with step size

$$\Delta V_{\text{read}} = \frac{V_{\text{read}}^{\text{max}}}{2^k}$$

A digitally defined schedule on the β word directly produces the desired $V_{\text{read}}(t)$, which can be updated at every sampling sweep or at a slower rate. In this scheme, the DAC provides a single global analog control, while the instantaneous spin configuration is imposed locally at the columns by digital selection of the column voltages (Ising or QUBO encoding as described above). This separation of concerns keeps the architecture scalable: the annealing logic does not grow with problem size, while the array and column drivers scale with the number of spins.

Suppl. Fig. 9 shows a projected circuit architecture implementing these ideas: signed couplings are stored in differential memristor pairs, and each spin input s_j generates a voltage $V_j = s_j V_{\text{read}}$ applied in differential form as $(+V_j, -V_j)$. Each row includes its own readout and stochastic neuron, enabling parallel updates.

SMTJ switching rate and sampling throughput

The maximum sampling rate of the architecture is ultimately limited by the characteristic switching times of the stochastic MTJ neurons. Each update pulse must be long enough for the MTJ to explore its thermally activated dynamics under the applied V_{mtj} and produce a statistically meaningful sample.

On the CMOS-integrated prototype used in this work, the perpendicular-anisotropy MTJs exhibit a broad distribution of dwell times: some devices show sub-100-ns switching, while others remain in a given state for more than 1 µs. To ensure robust operation across this diversity and avoid biasing the results towards only the fastest devices, we used a conservative read-pulse duration of 50 µs in the experiments, which is much longer than the intrinsic times of the faster junctions.

These values are specific to the present integration run and do not reflect a fundamental limit of stochastic PMA MTJs. Independent measurements on a different sample (Suppl. Fig. 10) demonstrate PMA devices with mean dwell times as low as 3.6 ns when biased at the equiprobable voltage. Such switching statistics support update rates in the hundreds of megahertz range and show that sub-10-ns stochastic operation is achievable in practice. In a parallel architecture of the type sketched in Suppl. Fig. 9, this would directly translate into multi-hundred-megahertz global update rates, provided that array access and peripheral circuitry are co-designed to match the MTJ dynamics.

Projected energy and comparison to existing Ising machines

The absolute energy consumption of the present benchtop experiment is dominated by laboratory instruments and long PCB traces, and is therefore not meaningful for comparison with integrated Ising solvers. Instead, we provide here order-of-magnitude estimates for a realistic integrated implementation of our architecture and discuss how it compares qualitatively to existing approaches.

In an integrated implementation, the dominant energy contributions are:

1. the energy dissipated in the p-bit circuits (including the SMTJ),

2. the energy dissipated in the memristor array during the MAC operation, and

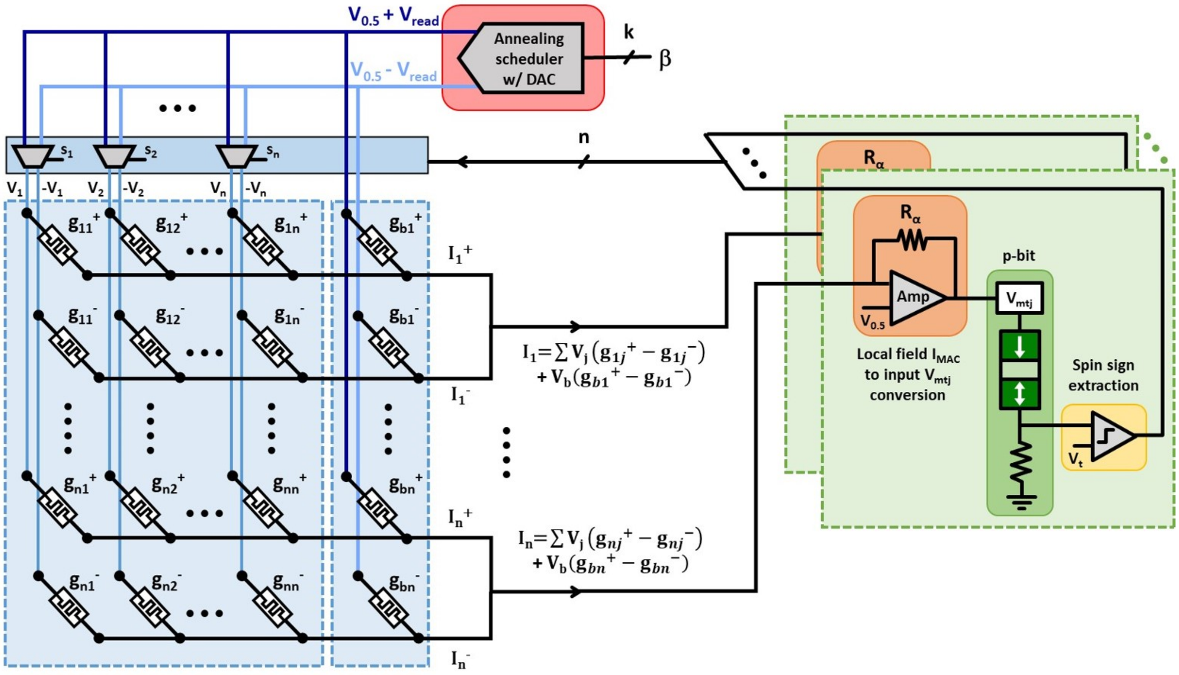

Supplementary Figure 9. Projected circuit architecture enabling parallel updates. The crossbar array (blue) stores each coupling J_{ij} in a differential memristor pair (g_{ij}^{+}, g_{ij}^{-}). For column j, the spin value s_j controls a differential driver that routes the read voltage V_{read} with the appropriate polarity, applying $(+V_j, -V_j)$ with $V_j = s_j V_{\text{read}}$ to the two columns. (In practice, these voltages are implemented as small offsets around the common reference level $V_{0.5}$, consistent with the optimization-mode operation described in the Methods.) The bias term is handled by the same differential mechanism. Each pair of rows collects the resulting currents I_i^{+} and I_i^{-}, whose difference implements the local field. This current is converted into the SMTJ control voltage V_{mtj} by a local transimpedance stage (orange), and the SMTJ output is digitised by the comparator block (yellow) to produce the updated spin s_i. Each row includes its own amplifier, p-bit (green), and comparator, enabling fully parallel updates. When the problem is expressed in its QUBO form, only a single positive V_{read} is required, and each column driver selects between V_{read} (for $x_j = 1$) and the reference level $V_{0.5}$ (for $x_j = 0$). The annealing scheduler modulates V_{read} according to the chosen temperature schedule.

3. the energy of the simple analog front-end (op-amp-based circuit) that converts the MAC current into the voltage applied to the SMTJ (Fig. 2a, main text).

For (1), prior work from the Camsari group has shown that, with nanosecond-class SMTJs, p-bit sampling energy can be pushed down to the femtojoule-per-bit range[10]. For (2), the absence of an ADC at the array output is a key advantage of our architecture: in standard memristor-based accelerators, the MAC energy is often dominated by the ADC, whereas here the MAC current directly biases the SMTJ. Using representative parameters (10 ns read time, device resistance on the order of 50 kΩ and sub-0.2 V read voltages), the energy per device read is on the order of a few femtojoules, i.e., $\sim$ fJ $\times N$ per p-bit update for an $N \times N$ problem. For (3), simple sizing of an op-amp in a commercial 22 nm CMOS process suggests that, for dense arrays, the front-end energy can be made comparable to the energy dissipated in the memristor array itself. These conservative estimates are consistent with

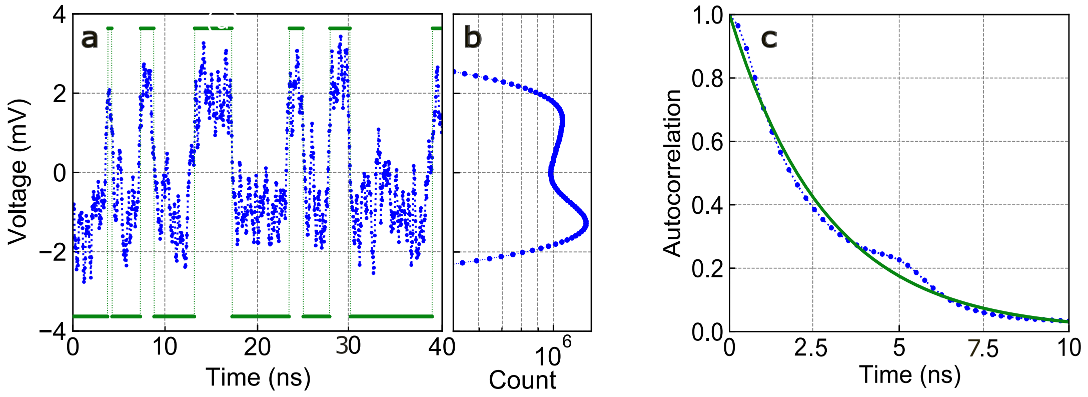

Supplementary Figure 10. Fast stochastic dynamics of a 50-nm PMA MTJ. a Forty-nanosecond segment of a voltage trace recorded under an in-plane field of 60 mT and a mean read current of 15 μA, showing five spontaneous transitions (blue) together with a digitised replica (green). **b** Histogram of the voltage levels accumulated over a 10-ms acquisition containing more than 10^6 transitions. **c** Autocorrelation of the trace in **a**, with an exponential fit (green) yielding a characteristic dwell time of 3.6 ns.

sub-picojoule energies per spin update and total energies in the tens of nanojoules for optimization runs involving 10^4–10^5 updates on practically relevant problem sizes.

Qualitatively, this leads to the following comparison with state-of-the-art Ising solvers:

- Pure CMOS approaches incur high energy and area costs in both MAC operations and random-number generation (RNG)[10].
- Hybrid CMOS+SMTJ approaches reduce the RNG cost, but still rely on energy-intensive CMOS MACs and substantial data movement between memory and compute[11–14].
- Memristor- or FeFET-based Ising machines reduce the MAC energy by computing in memory, but typically require both high-resolution ADCs at the array output and additional RNG circuitry[15–19].
- Our hybrid memristor+SMTJ architecture simultaneously removes the ADC overhead and embeds the stochasticity directly in the non-volatile p-bits at the periphery of the array, thereby reducing both MAC and RNG costs in principle.

Because problem sizes, encodings, and reporting methodologies vary widely across the current literature on Ising machines, a direct and fair energy-per-solution comparison is not yet possible. As discussed, for instance, at the 2025 International Workshop on Ising Machines in Chicago, the community is moving toward more standardized benchmarks, but this effort is still ongoing. For this reason, we restrict ourselves here to conservative, architecture-level estimates and qualitative comparisons, rather than quoting precise numerical speed-up factors relative to specific prior implementations.

Scaling of the memristor array

In contrast to most analog in-memory computing (aIMC) accelerators for neural networks, our memristor MAC stage does not require an ADC at the array output: the summed current directly biases the

SMTJ. As a result, the energy and latency per spin update are not dominated by high-resolution data conversion, but instead by the characteristics of the crossbar itself (array size, line resistance and capacitance) and by the chosen conductance range of the devices.

For a given technology node, the MAC latency is set by the RC time constants of the word and bit lines and by the bandwidth of the local transimpedance stage that converts MAC current into the SMTJ voltage. The corresponding dynamic energy per update scales approximately with

$$E_{\mathrm{MAC}} \sim C_{\mathrm{line}}\, V_{\mathrm{read}}^2$$

per active line, with C_{line} the effective line capacitance and V_{read} the pseudo-temperature–controlled read amplitude. Increasing the conductance window of the memristors boosts the MAC signal and improves SNR (as discussed in Supplementary Note 4), but also raises static and dynamic power and exacerbates IR drop and non-uniformity along the lines. These trade-offs are well known in aIMC crossbars and typically lead to a design point where arrays are kept to moderate sizes and then tiled[20–22].

In a realistic implementation, large Ising graphs would therefore be mapped onto a set of tiles (for example 64×64 to 256×256 2T1R or 1T1R arrays), each operating as a local MAC engine. Per-tile calibration and hierarchical control, already adopted in state-of-the-art analog neural accelerators[22], can be reused here to compensate device-to-device variation, IR drop, and mild nonlinearity while retaining the benefits of analog parallelism. Because our architecture uses a single global knob, V_{read}, to implement annealing, these measures remain fully compatible: the annealing schedule simply scales the read amplitudes for all tiles simultaneously, without altering the internal MAC structure.

Spatial scaling beyond the footprint of a single die can be achieved with 2.5D and 3D integration. In a 2.5D configuration, multiple memristor tiles and SMTJ blocks are placed side by side on a silicon interposer, which provides dense passive routing and shortens interconnects from PCB scale to sub-millimetre distances[23,24]. Hybrid 3D bonding pushes this further by joining prefabricated wafers face to face, achieving micrometre-scale vertical connections between crossbar tiers and peripheral logic without incurring the full thermal budget of sequential BEOL processing[25,26]. Ultimately, monolithic 3D BEOL stacking, in which multiple memristor layers and logic tiers are fabricated in a single backend flow, can deliver sub-100-nm vertical vias and very high integration density[25–27]. Technology roadmaps for aIMC and neuromorphic computing identify such monolithic 3D stacks as a key enabler for scaling to very large arrays with high bandwidth and low latency[28,29].

In this context, our hybrid memristor–SMTJ Ising machine benefits twice from scaling: (i) the absence of ADCs at the array outputs reduces the energy and area overhead per MAC tile compared with conventional aIMC, and (ii) placing MAC tiles and SMTJ neurons in close proximity through 2.5D or 3D integration shortens the path between memory and p-bits to the micrometre or sub-micrometre range. Combined with the nanosecond-scale stochastic dynamics demonstrated for advanced SMTJs (Supplementary Fig. 10), this offers a realistic route to large-scale, parallel Ising solvers in which both memory access and sampling operate in the nanosecond regime.

Supplementary Note 7: Pulse-based programming for mapping matrices

This Note details the pulse-based programming protocols used to map the Max-Cut and graph-coloring matrices onto the memristor array. Unless otherwise stated, all programming pulses have a rise time of 10 ns. Our devices are based on a classic TiN/HfO_x/Ti/TiN stack and are integrated in arrays with NMOS access transistors (see Methods, main paper). Suppl. Fig. 11 shows the pulsed current–voltage characteristic of three memristors.

We employ a stepwise set-based programming protocol to drive the device into the desired conductance range for the Ising machine experiments.

Forming

In a first forming step, three to four identical 20 µs pulses are applied with the word line (WL) at +1.0 V, the bit line (BL) at +2.0 V, and the source line (VSL) at 0 V, which raises the conductance to approximately 30-40 µS.

Set-based programming of memristor states

Next, WL is gradually increased from 1.0 V to between 1.1 and 1.2 V (with BL held at 2.0–2.1 V and VSL at 0 V), using the same 20 µs pulse width, until the conductance reaches 50–70 µS (Suppl. Fig. 12a). Finally, WL is raised to 1.4 V (BL and VSL unchanged) under 20 µs pulses to push the device conductance to about 100 µS. While this purely set-based approach allows us to span the full 30–100 µS range, we observe significant temporal drift at the highest conductance values.

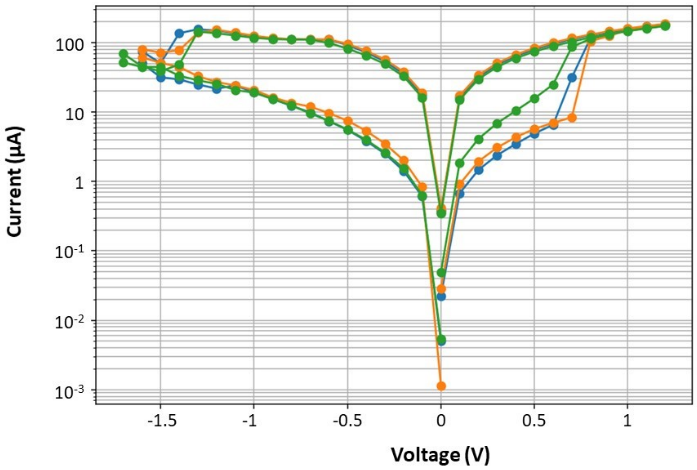

Supplementary Figure 11. Pulsed I-V curve of three memristors in a 1T1R structure. Positive side (set process): WL voltage fixed at 2.5 V, SL at 0 V, BL swept back and forth from 0 V to 1.3 V in 0.1 V steps. Negative side (reset process): WL voltage fixed at 4.2 V, BL at 0 V, SL swept back and forth from 0 V to 1.7 V in 0.1 V steps. All pulses are 20 µs-long. The three memristors were chosen randomly in one of our memristor arrays.

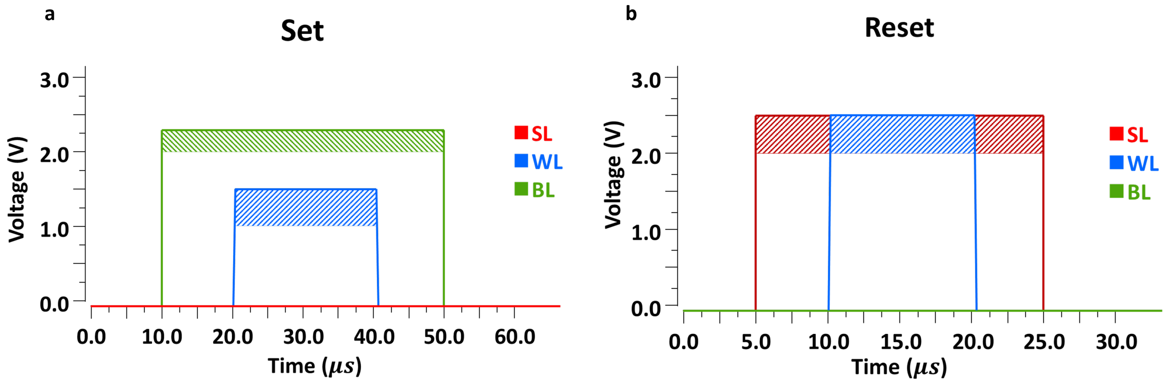

Supplementary Figure 12. Programming techniques to map the matrices. a Set-based programming technique. **b** Reset-based programming technique.

Drift-resilient reset-based programming of memristor states

To mitigate this drift and obtain precise, stable conductance levels, we developed a progressive reset-based tuning procedure. First, we intentionally overshoot each target conductance by about 20–30 µS using the set protocol (for example, a target of 33 µS is initially brought to 50–60 µS, 66 µS to 90–100µS, and 99 µS to 120–130 µS). We then apply repeated 20 µs reset pulses with WL and VSL stepped between 2.0 and 2.5 V while BL is grounded (Suppl. Fig. 12b). Each pulse reduces the conductance by roughly 5–10 µS (e.g., from 130 µS down to 125 µS, 120 µS, 112 µS, etc.) until the device is within ±5 µS of the target value. Finally, we perform a stability readout every 10 s for 2–3 min to verify that the conductance remains within this tolerance window. This two-step "set then progressive reset" scheme provides both accurate conductance tuning and enhanced temporal stability across the dynamic range required for the analog MAX-CUT implementations.

For the graph-coloring experiments, one of the conductance levels must be programmed very precisely to 140 µS. In this case, we further refine the programming sequence. First, we follow the set-based protocol described above to drive each device from its pristine state up to approximately 110 µS. We then apply a stronger 20 µs reset pulse with BL grounded and WL and VSL both set to 2.5 V, which typically places the conductance in the 160–170 µS range. Subsequent 10 µs reset pulses gradually reduce the conductance (e.g., to 165 µS, 162 µS, 156 µS, and so on) until it approaches the desired 140 µS level. To avoid overshooting below 135 µS, we dynamically decrease WL and VSL from 2.5 V down toward 2.3 V during this final tuning phase, thereby ensuring fine control over each $\sim$ 5–10 µS decrement.

Supplementary Note 8: Large-scale Graph-Coloring benchmark on the Les Misérables network

This Supplementary Note presents additional results on a large-scale graph-coloring benchmark. The graph-coloring experiment discussed in the main text corresponds to a 3-coloring instance on a 10-vertex graph with moderate connectivity. While this problem is already nontrivial and cannot be solved by simple greedy heuristics, it was primarily selected to demonstrate the implementation of mixed-sign couplings and bias terms within the same hardware framework.

To explore the performance of the system on substantially larger instances, we consider the *Les Misérables* character network[30]. In this graph, each vertex represents a character from Victor Hugo's *Les Misérables*, and edges connect pairs of characters who co-occur in the same chapter. The resulting coloring problem enforces that no two characters appearing together share the same color. The corresponding instance is formulated as a 10-coloring problem on a 77-vertex graph with higher average connectivity ($C = 6.59$); the one-hot encoding over 10 colors expands the problem to 770 nodes while significantly sparsifying the effective connectivity.

Suppl. Fig. 13a shows the corresponding adjacency matrix. At this scale, the sparse interaction structure gives rise to a large number of nominally zero couplings. Small residual conductance offsets associated with these couplings can accumulate when summed over many connections and become non-negligible compared to the programmed interaction strengths. This effect represents a scale-dependent nonideality that is negligible in smaller instances but becomes increasingly relevant as problem size grows. Suppl. Fig. 13b shows the evolution of the cost function during a representative run under nominal conditions, illustrating convergence to a low-energy configuration under a simulated annealing schedule consistent with the hardware implementation. A corresponding valid coloring solution is shown in Fig. 13c.

To model device-level variability, nominal zero-conductance states are represented by half-normal distributions, as in Supplementary Note 1, and all conductance levels are subject to Gaussian fluctuations with adjustable standard deviation. When only the single conductance matrix shown in Fig. 13a is used, these residual offsets accumulate over the many nominally zero entries in each row, leading to a rapid degradation of solver performance even at low variability levels. To counteract this effect, a straightforward differential weight encoding scheme consists of subtracting a reference matrix containing only nominally zero entries. In this way, residual offsets are largely canceled through subtraction of closely matched conductance values. (The use of differential cells to represent weights is a standard technique in memristor-based neural networks[31,32].)

Suppl. Fig. 13d summarizes the success probability as a function of conductance variability for both single-matrix and differential encoding schemes. While the single-matrix representation exhibits a rapid loss of accuracy as variability increases, the differential encoding preserves high success rates (>90%) up to experimentally achievable variability levels (standard deviation ≈2 µS). These results demonstrate that residual errors associated with nominally zero couplings, which become increasingly important in large and sparse graphs, can be effectively mitigated through the proposed architectural mapping.

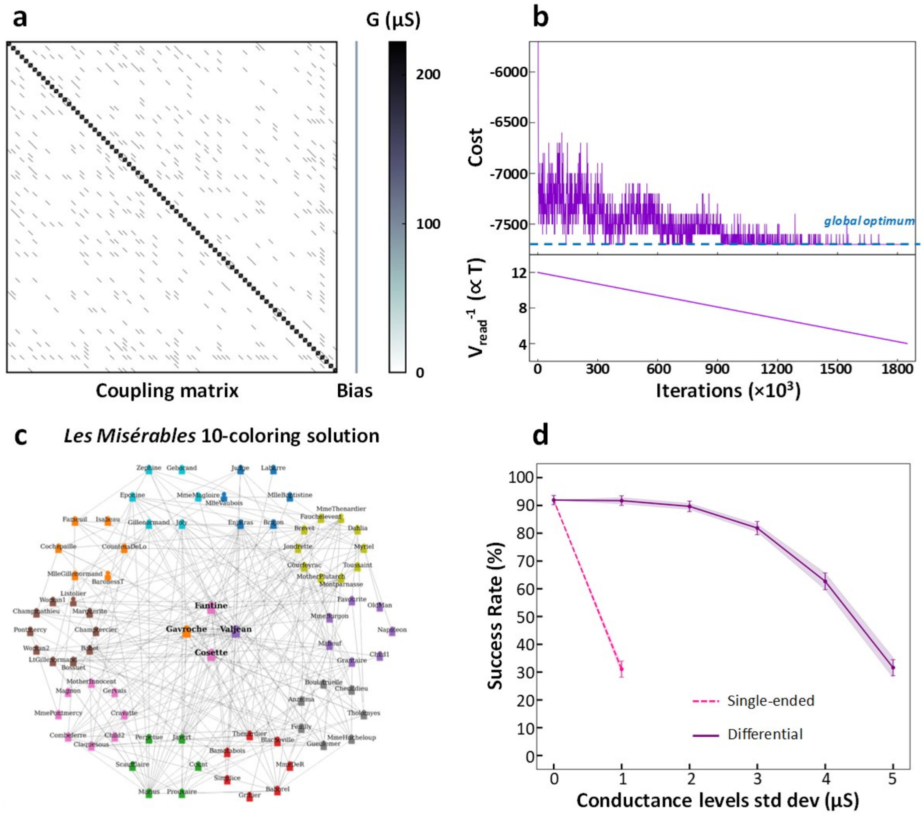

Supplementary Figure 13. Large-scale graph-coloring benchmark on the *Les Misérables* network. a One-hot encoding of a 77-vertex, 10-color graph-coloring instance mapped onto a sparse 770×770 conductance matrix. **b** Evolution of the Ising cost function during a representative run using a linear annealing schedule implemented by ramping the read voltage, obtained using nominal (noise-free) conductance values. The dashed line indicates the known global optimum. **c** Final coloring configuration, showing ten clusters of characters from Victor Hugo's *Les Misérables*, within which no two connected vertices share the same color. **d** Success probability as a function of conductance variability (standard deviation) for single-matrix and differential encoding schemes. While the single-ended representation rapidly degrades with increasing variability, the differential encoding preserves high success rates for experimentally achievable variability levels.

Supplementary References

1. Wiegele, A. Biqmac library: A collection of max-cut and quadratic 0-1 programming instances of medium size (2009).
2. Pallo, M., D'Agostino, S., Piccoli, M., Bonnet, D., Castellani, N., Piccolboni, G., Iftakher, M., Nodin, J.-F., Andrieu, F., Querlioz, D. *et al.* On chip customized learning on resistive memory technology for secure edge ai. In *2025 Symposium on VLSI Technology and Circuits (VLSI Technology and Circuits)*, 1–3 (IEEE, 2025).
3. Rao, M., Tang, H., Wu, J., Song, W., Zhang, M., Yin, W., Zhuo, Y., Kiani, F., Chen, B., Jiang, X. *et al.* Thousands of conductance levels in memristors integrated on cmos. *Nature* **615**, 823–829 (2023).
4. Park, J., Kumar, A., Zhou, Y., Oh, S., Kim, J.-H., Shi, Y., Jain, S., Hota, G., Qiu, E., Nagle, A. L. *et al.* Multi-level, forming and filament free, bulk switching trilayer rram for neuromorphic computing at the edge. *Nat. Commun.* **15**, 3492 (2024).
5. Stecconi, T., Guido, R., Berchialla, L., La Porta, A., Weiss, J., Popoff, Y., Halter, M., Sousa, M., Horst, F., Dávila, D. *et al.* Filamentary taox/hfo2 reram devices for neural networks training with analog in-memory computing. *Adv. electronic materials* **8**, 2200448 (2022).
6. Le Gallo, M., Khaddam-Aljameh, R., Stanisavljevic, M., Vasilopoulos, A., Kersting, B., Dazzi, M., Karunaratne, G., Brändli, M., Singh, A., Mueller, S. M. *et al.* A 64-core mixed-signal in-memory compute chip based on phase-change memory for deep neural network inference. *Nat. Electron.* **6**, 680–693 (2023).
7. Ambrogio, S., Narayanan, P., Okazaki, A., Fasoli, A., Mackin, C., Hosokawa, K., Nomura, A., Yasuda, T., Chen, A., Friz, A. *et al.* An analog-ai chip for energy-efficient speech recognition and transcription. *Nature* **620**, 768–775 (2023).
8. Brown Jr, W. F. Thermal fluctuations of a single-domain particle. *Phys. review* **130**, 1677 (1963).
9. Rippard, W., Heindl, R., Pufall, M., Russek, S. & Kos, A. Thermal relaxation rates of magnetic nanoparticles in the presence of magnetic fields and spin-transfer effects. *Phys. Rev. B* **84**, 064439 (2011).
10. Singh, N. S., Kobayashi, K., Cao, Q., Selcuk, K., Hu, T., Niazi, S., Aadit, N. A., Kanai, S., Ohno, H., Fukami, S. *et al.* Cmos plus stochastic nanomagnets enabling heterogeneous computers for probabilistic inference and learning. *Nat. Commun.* **15**, 2685 (2024).
11. Borders, W. A., Pervaiz, A. Z., Fukami, S., Camsari, K. Y., Ohno, H. & Datta, S. Integer factorization using stochastic magnetic tunnel junctions. *Nature* **573**, 390–393 (2019).
12. Kaiser, J., Borders, W. A., Camsari, K. Y., Fukami, S., Ohno, H. & Datta, S. Hardware-aware in situ learning based on stochastic magnetic tunnel junctions. *Phys. Rev. Appl.* **17**, 014016 (2022).
13. Yin, J. *et al.* Scalable ising computer based on ultra-fast field-free spin orbit torque stochastic device with extreme 1-bit quantization. In *2022 International Electron Devices Meeting (IEDM)*, 36.1.1–36.1.4 (IEEE, 2022).

14. Si, J., Yang, S., Cen, Y., Chen, J., Huang, Y., Yao, Z., Kim, D.-J., Cai, K., Yoo, J., Fong, X. *et al.* Energy-efficient superparamagnetic ising machine and its application to traveling salesman problems. *Nat. Commun.* **15**, 3457 (2024).

15. Cai, F., Kumar, S., Van Vaerenbergh, T., Sheng, X., Liu, R., Li, C., Liu, Z., Foltin, M., Yu, S., Xia, Q., Yang, J. J., Beausoleil, R., Lu, W. D. & Strachan, J. P. Power-efficient combinatorial optimization using intrinsic noise in memristor hopfield neural networks. *Nat. Electron.* **3**, 409–418 (2020).

16. Jiang, M., Shan, K., He, C. & Li, C. Efficient combinatorial optimization by quantum-inspired parallel annealing in analogue memristor crossbar. *Nat. communications* **14**, 5927 (2023).

17. Kim, K., Youn, S., Park, J. & Kim, H. Ising solver using weight profile of memristor crossbar array for combinatorial optimization. In *2024 IEEE International Electron Devices Meeting (IEDM)*, 1–4 (IEEE, 2024).

18. Shan, K., Jiang, M., Du, Z., Xiao, Y., Tong, Y., Li, Z., Yang, S.-H., He, C., Mao, R., Lin, P. *et al.* One-step combinatorial optimization solver with fully integrated analog memristors and annealing module. In *2024 IEEE International Electron Devices Meeting (IEDM)*, 1–4 (IEEE, 2024).

19. Yin, X., Qian, Y., Vardar, A., Günther, M., Müller, F., Laleni, N., Zhao, Z., Jiang, Z., Shi, Z., Shi, Y. *et al.* Ferroelectric compute-in-memory annealer for combinatorial optimization problems. *Nat. Commun.* **15**, 2419 (2024).

20. Hu, M., Strachan, J. P. & Li, C. Memristor-based analog computation and neural network acceleration. *Nat. Electron.* **1**, 324–332 (2018).

21. Boybat, I., Le Gallo, M., Nandakumar, S. R., Moraitis, T., Piveteau, C., Tuma, T., Rajendran, B., Leblebici, Y., Sebastian, A. & Eleftheriou, E. Neuromorphic computing with multi-memristive synapses. *Nat. Commun.* **9**, 2514 (2018).

22. Ambrogio, S., Narayanan, P., Tsai, H., Shelby, R. M., Boybat, I., di Nolfo, C., Sidler, S., Giordano, M., Bodini, M., Farinha, N. C. P., Killeen, B., Cheng, C.-F., Jaoudi, Y. & Burr, G. W. Equivalent-accuracy accelerated neural-network training using analogue memory. *Nature* **558**, 60–67 (2018).

23. Park, J., Lee, B., Lee, H., Lim, D., Kang, J., Cho, C., Na, M. & Jin, I. Wafer to wafer hybrid bonding for dram applications. In *2022 IEEE 72nd Electronic Components and Technology Conference (ECTC)*, 126–129 (2022).

24. Gorchichko, M., Sharma, S., Ng, B., Sherwood, T., Jeon, Y., Mcintyre, D., Li, K., Singh, S., Iler, E., Knapp, D., Prakash, A., Nguen, V., Sreenivasan, R., Krishnan, S. & Chudzik, M. Novel low thermal budget bonding using single wafer thermal processing system, resulting in excellent wafer-to-wafer hybrid bonding at sub-0.5um pitch. In *2024 IEEE 74th Electronic Components and Technology Conference (ECTC)*, 404–407 (2024).

25. Chakrabarti, B., Lastras-Montaño, M. A., Adam, G., Prezioso, M., Hoskins, B., Payvand, M., Madhavan, A., Ghofrani, A., Theogarajan, L., Cheng, K.-T. & Strukov, D. B. A multiply-add engine with monolithically integrated 3d memristor crossbar/cmos hybrid circuit. *Sci. Reports* **7**, 42429 (2017).

26. Li, Y. *et al.* Monolithic three-dimensional integration of rram-based hybrid memory architecture for one-shot learning. *Nat. Commun.* **14**, 7140 (2023).

27. Liu, S., Radway, R. M., Wang, X., Moro, F., Nodin, J.-F., Jana, K., Du, S., Upton, L. R., Chen, W.-C., Chen, J. *et al.* Edge continual training and inference with rram-gain cell memory integrated on si cmos. In *2024 IEEE International Electron Devices Meeting (IEDM)*, 1–4 (IEEE, 2024).

28. Liu, S., Radway, R. M., Wang, X., Kwon, J., Trippel, C., Levis, P., Mitra, S. & Wong, H.-S. P. Future of memory: Massive, diverse, tightly integrated with compute - from device to software. In *2024 IEEE International Electron Devices Meeting (IEDM)*, 1–4 (IEEE, 2024).

29. Vianello, E. & Payvand, M. Scaling neuromorphic systems with 3d technologies. *Nat. Electron.* **7**, 419–421 (2024).

30. Knuth, D. E. *The Stanford GraphBase: A Platform for Combinatorial Computing* (Addison-Wesley, Reading, MA, 1993).

31. Prezioso, M., Merrikh-Bayat, F., Hoskins, B. D., Adam, G. C., Likharev, K. K. & Strukov, D. B. Training and operation of an integrated neuromorphic network based on metal-oxide memristors. *Nature* **521**, 61–64 (2015).

32. Wan, W., Kubendran, R., Schaefer, C., Eryilmaz, S. B., Zhang, W., Wu, D., Deiss, S., Raina, P., Qian, H., Gao, B. *et al.* A compute-in-memory chip based on resistive random-access memory. *Nature* **608**, 504–512 (2022).
