# Supplementary material for: Intrinsic annealing in a hybrid memristor-magnetic tunnel junction Ising machine
Source: Nat Commun. 2026 Apr 16;17:5246. doi: 10.1038/s41467-026-71844-8 (PMC13260843; doi:10.1038/s41467-026-71844-8)
Supplement: Supplementary file 2 — Transparent Peer Review file [file 41467_2026_71844_MOESM2_ESM.pdf]

nature portfolio

Peer Review File

Intrinsic Annealing in a Hybrid Memristor-Magnetic Tunnel Junction Ising Machine

Corresponding Author: Dr Damien Querlioz

This file contains all reviewer reports in order by version, followed by all author rebuttals in order by version.

Version 0:

Reviewer comments:

Reviewer #1

(Remarks to the Author)
The manuscript by Iftakher et al. presents a hybrid hardware Ising machine that integrates hafnium-oxide memristor crossbar arrays with stochastic magnetic tunnel junctions (SMTJs) to achieve intrinsic voltage-controlled annealing. The memristor crossbar array is used for MAC operation, while sMTJ is used for stochastic switching. Both parts have been well studied before. While combining them might indeed bring some benefits, the overall approach is relatively straightforward. The experimental demonstrations on MAX-CUT and graph coloring problems are encouraging, but several critical issues need to be addressed before recommending this work for publication in Nature Communications.

First, the authors claim that ADC is not needed in this system, which would be a significant advantage. However, has the analog connection between memristor crossbar array and SMTJ been experimentally demonstrated? The current setup appears to use separate dies with PCB connections and lab equipment in between. If this direct analog coupling has been achieved, the authors should provide detailed characterization. If not, this should be experimentally realized as it represents the core innovation of this paper.

The annealing method used in the paper tuned the Vread applied to the array. However, changing Vread might also have an impact on the signal-to-noise ratio (SNR). Will this effect influence the system performance? The authors should plot how the SNR changes with different Vread (similar plots with Fig. 2b but under different Vread). This is crucial because if SNR degrades significantly at low Vread (high temperature regime), it could compromise the exploration phase of annealing, while poor SNR at high Vread could affect the final solution quality.

As noted, the demonstrated problems are sparse (85.4% and 82% zero couplings). Many real-world optimization problems have dense connectivity. Is there any limitation for the system with the problem?em density ? If no, could the author demonstrate a denser problem?

The demonstrated problems are relatively small (24-node MAX-CUT and 10-node graph coloring). For a technology claiming to offer "a scalable pathway toward compact, fast, and energy-efficient large-scale Ising solvers,". Could the authors demonstrate the approach on larger problem instances, or at least provide a clear analysis of what limits the current implementation from scaling?

While the authors claim potential nanosecond operation and energy efficiency advantages, no actual timing or energy measurements are provided. The current implementation uses sequential Gibbs sampling with 50 µs pulses, which seems far from the claimed nanosecond capability. What is the actual time-to-solution and energy consumption for the demonstrated problems? How does this compare with state-of-the-art digital or other hardware Ising solvers (e.g., memristor+CMOS, CMOS+sMTJ)?

The manuscript mentions "significant die-to-die variability" in SMTJs but doesn't analyze how this impacts system performance. In a scaled system with thousands of SMTJs, how would such variability affect solution quality and convergence? Similarly, what about memristor conductance variations and drift over time?

Reviewer #2

(Remarks to the Author)
The manuscript presents a novel hardware-based approach to solving combinatorial optimization problems. The authors propose an Ising machine architecture that integrates two distinct nanodevices: a memristor array for multilevel coupling and bias storage, and superparamagnetic magnetic tunnel junctions (sMTJs) functioning as probabilistic bits (p-bits) to enable stochastic computation. Notably, the annealing parameter is internally controlled by tuning the voltage applied to the sMTJs, which is directly derived from the current outputs of multiply-accumulate (MAC) operations performed by the memristor crossbar array. The system is demonstrated on small-scale optimization tasks, including a 24-node Maxcut problem and a 10-node graph coloring problem with bias constraints. The work is original and provides meaningful insights into hybrid memory-based Ising machines. However, the authors need to satisfactorily address the questions and suggested improvements outlined below before the work can be reevaluated for publication.

While the authors successfully demonstrate the solution of small-scale optimization problems, it remains unclear how the proposed system would scale to larger problem sizes. The performance should be evaluated as a function of problem size or complexity to assess scalability. In addition, the authors should test on more complex or standardized benchmark problems and compare their method with existing solvers. Specifically, how would the architecture perform when scaled to accommodate a significantly higher number of nodes or p-bits?

Given practical limitations in memristor crossbar array sizes (typically projected to be on the order of 128×128 or 256×256), the authors can provide projections on the scalability and architectural feasibility for larger problems (e.g., Maxcut instances with 100 nodes or more).
At such scales, would the intrinsic annealing approach via voltage modulation based on crossbar MAC outputs remain effective?

There is no discussion on energy consumption, speed, or area of the proposed system. Nor is there a comparison with other state-of-the-art Ising solvers. Without these key metrics, it is difficult to evaluate the practical advantages of the approach. A quantitative analysis of the energy consumption and performance characteristics of the proposed hybrid system would be beneficial. Estimation of the energy required to solve the optimization problems presented and how power and latency are influenced by the interaction between the memristor crossbar array and the sMTJs.

In addition to this, it would be useful to understand how these metrics vary with graph sparsity. While the authors mention that the test graphs used are approximately 80% sparse, a broader evaluation across different graph densities ranging from fully connected to highly sparse would help clarify how sparsity affects the hybrid system.

In the present prototype, a host computer, a microcontroller, and two Keysight B1530A SMUs sequentially (i) apply Vread, (ii) measure cross-bar currents, (iii) bias a single sMTJ via a probe station, and (iv) update the schedule via Python. This remains an instrument-in-the-loop demonstration. The claim of “nearly circuit-free annealing” is overstated; an integrated system that hard-wires the resistor path and voltage ramp would be more appropriate, especially given that stand-alone memristor and MTJ systems have been widely demonstrated.

Figure 1a gives the misleading visual impression of a fully integrated on-chip loop, yet the SMUs, microcontroller, and host computer are omitted. A workflow diagram of the complete experimental path should be included.

The graph coloring problem is demonstrated using a 30×30 memristor array, raising the question of whether a 30-node Maxcut problem could also be implemented to explore the upper limits of the system's problem size. The current 24-node limitation may stem from the use of three distinct multilevel coupling weights. If this is the case, the authors should explicitly discuss the tradeoffs between the number of weight levels that can be represented and the number of nodes that can be accommodated within the current hardware constraints.

Clarifying these limitations would help in understanding the design space and scalability of the system. Such tradeoff analysis could complement the projections regarding system scaling. Furthermore, shedding light on programming negative weights would ascertain the flexibility for implementing various problems sets.

Only three MTJs are sampled for the MAX-CUT instance; error bars across multiple devices are necessary. The known optimal solutions should be explicitly marked in Figures 3 and 4.

One of the highlights of this work is the intrinsic annealing strategy employed by the system as mentioned by the authors. However, the manuscript lacks sufficient detail on how the annealing schedule specifically the β (inverse temperature) parameter is modulated and implemented in hardware. It is unclear whether β is controlled digitally, or if it is inherently embedded within the operation of the memristor crossbar and its associated peripheral circuitry.

The authors briefly mention that an annealing scheduler modulates +VRead and −VRead via global DACs, but the description is superficial and lacks technical elaboration. It would be helpful if the authors clarified whether this modulation involves lookup tables, programmable voltage ramps, or other control logic, and how flexible or reconfigurable the annealing schedule is in practice.

The intrinsic annealing eliminates the need for ADCs between the two nanodevice-based memory modules as proposed by the authors. However, in the current experimental setup, these voltages are generated and modulated via an external instrument (B1530A), which reads currents and outputs corresponding voltages. It remains unclear how faithfully this voltage signal would be reproduced at the circuit or silicon level in a fully integrated system. Furthermore, as the system scales,

potential variations and drops in the analog voltage path may significantly affect the fidelity of the annealing process. The authors can provide further discussion or analysis on such effects.

The authors should include brief details regarding the formation, SET, and RESET processes of the fabricated memristor bitcells used in their system. Providing representative current vs voltage (I-V) characteristics and examples of the programming pulses applied during device operation would details of the electrical behavior.

How many devices were fabricated and utilized in the experiments? While the authors mention executing the Maxcut problem on three different devices, it is not clear whether these devices are connected in an array formation or operated individually. If possible, the authors could expand on multiple sMTJ devices mapped to represent Ising spins, even if they are updated sequentially, to demonstrate the scalability.

Assigning a unique Rα to each SMTJ poses significant scalability challenges. Characterizing millions of MTJs individually would be impractical. Can the authors explore the possibility of using fixed or nominal Rα values? If so, how does this simplification affect the algorithm's performance?

SMTJs typically have low energy barriers and are thus sensitive to temperature fluctuations. How does device heating influence experimental results and whether any thermal mitigation strategies were employed?

The manuscript references the presence of both slow and fast sMTJs, but does not provide sufficient detail on how these categories are defined. Are the devices classified based on the minimum pulse width required to capture a complete switching probability curve within a given voltage range?

If such device-level variations exist, it raises issues with the reliability of computation in larger systems, leading to degraded solution quality when intrinsic annealing voltages are applied. The authors should discuss how these variations are managed or whether calibration per bitcell would be necessary in a scaled implementation.

The abbreviation sMTJ appears to refer to both superparamagnetic MTJ and stochastic MTJ in different parts of the manuscript. As stochastic MTJ is a more generic term, please clarify.

In Figure 1(a), the interconnections between the two memory components are not clearly illustrated. As the methods section references the use of external instrumentation these connections could be explicitly depicted in the schematic. This would help bridge the gap between the current setup and the claim of on-chip integration of the two memories.

Fig. 1a shows n-output currents from the memristor array, yet only a single MTJ is illustrated. It confuses how these outputs are experimentally connected to a single MTJ. The authors should clarify this mechanism in the text and revise the figure accordingly.

The coupling and bias matrices used for the Maxcut and graph coloring problems are not standard benchmark problems, it would be helpful if the authors could provide the matrices either in the supplementary material or as a code/data repository link for reproducibility.

The inverse temperature (β) to vary linearly, VRead must follow an exponential schedule. While this is visually suggested in Figure 3(b), it is not explicitly discussed in the main text.

In Figure 4(b), a legend can be included to indicate that the three curves correspond to independent iterations.

In Table 1, under the Problems Solved (size) row, it would be informative to also include the number of spins or p-bits used, coupling and bias matrix size in each implementation to have a consistent comparison across platforms, as different platforms solve various NP-hard problems.

In Supplementary Figure S1, the architecture diagram can include the global DACs mentioned in the main text.

Long-term stability is unsubstantiated. The MTJ is simulated for the repeat test. The authors must show repeated solve runs that include the MTJ.

Reviewer #3

(Remarks to the Author)
This manuscript presents an experimental demonstration of probabilistic computing using stochas-
tic magnetic tunnel junctions (sMTJs) to solve complex combinatorial problems such as MAX-CUT and graph coloring. The authors implement single-spin Metropolis-Hastings dynamics by reusing a single sMTJ for repeated updates, and benchmark their approach on representative graph problems. The work is technically sound, and of interest to the fields of spintronics, unconventional computing, and hardware acceleration for optimization.

I am generally positive about the work and recommend publication after the authors address the following points for clarification and completeness.

Major Comments

1. Details of the Benchmark Graphs:

More detail is needed on the graphs used for the MAX-CUT and 3-coloring experiments. In particular:

• What is the average connectivity C of the graph used in the 3-coloring experiment? If $C < 3$, the problem can be easily solved by a greedy coloring algorithm, which would make it a relatively weak benchmark.

• The graphs appear visually sparse. Sparse graphs tend to be easier to solve; this should be acknowledged and discussed.

• Please specify how the graphs are generated. Are they drawn from a random ensemble, or are they structured instances?

2. Single vs. Multiple Graph Instances:

The paper reports results for what appears to be a single instance of each graph problem. Why were multiple graphs not used for each problem type? Providing average performance across an ensemble would better validate the robustness and generality of the method.

Minor Questions

1. Spin Storage Architecture:
In the hardware implementation (Fig. 1a), a single sMTJ is used to emulate spin-flip dynamics. Where are the values of all other spins stored? The "Spins vector" is referenced, but is this a separate digital memory component or an array of sMTJs?

2. Programmability of Couplings Jij :
Can the crossbar architecture support both positive and negative couplings Jij ? In the MAX-CUT problem, all couplings are negative. In the coloring experiment, the couplings from each node are either all positive or all negative—a rather special case. Is it possible to realize arbitrary signed Jij in this hardware, and if not, what modifications would be required to support this functionality?

Summary

This is an interesting paper demonstrating a proof-of-concept for probabilistic computing using spintronic devices. The experimental system is well designed, and the benchmarks show promising results. I recommend publication after the authors address the above points, particularly those regarding the complexity and generality of the benchmark graphs and the programmability of the hardware.

Version 1:

Reviewer comments:

Reviewer #1

(Remarks to the Author)
I thank the authors for their detailed response and the significant effort put into revising the manuscript. The addition of the analog coupling experiment, the characterization of signal-to-noise ratio versus read voltage, and the variability simulations in Supplementary Note 1 have substantially strengthened the proposed device concepts and the overall quality of the work.

While Figure 2 successfully demonstrates that the read voltage can modulate the sMTJ switching probability via an operational amplifier, this experiment appears to be a characterization rather than a functional demonstration of the system solving a problem. Based on the authors' response, the actual Max-Cut and graph coloring demonstrations still relied on a computer-in-the-loop configuration where a Python script determined the feedback voltage. Consequently, the claim that the solver operates with intrinsic analog annealing has not been fully experimentally realized for an optimization task. The manuscript should be revised to explicitly clarify that the problem-solving results rely on software-mediated feedback and that the purely hardware-based analog loop has so far been limited.

Furthermore, there are remaining concerns regarding scalability that the current simulations do not fully address. More effects need to be considered when the problem is scaled for a fully analog system, such as systematic IR drop in a crossbar array that alters effective coupling values and interconnect parasitics like RC delay, which can introduce synchronization issues. Additionally, the simulations are currently limited to 100-node instances, which are not computationally too difficult for digital computers. Since these results are generated via simulation and are not constrained by fabrication limits, the authors should evaluate significantly larger instances (e.g., 800 to 2000 nodes) to verify that the analog dynamics and signal margins remain robust at a scale where this technology would offer a genuine advantage.

Reviewer #2

(Remarks to the Author)

The authors have adequately addressed the majority of the comments by providing detailed clarifications, additional figures in the main text, and expanded experimental explanations in the supplementary material. While large-scale hardware integration beyond simulation remains as future work, the manuscript clearly demonstrates a functional prototype leveraging SMTJ and RRAM devices for Ising machines. This work represents a meaningful contribution to the field. I therefore recommend this work for publication.

Version 2:

Reviewer comments:

Reviewer #1

(Remarks to the Author)
The manuscript has been improved through clarifications added throughout, with additional discussion that may be useful to readers; accordingly, it is ready for publication in its present form.

Open Access This Peer Review File is licensed under a Creative Commons Attribution 4.0 International License, which permits use, sharing, adaptation, distribution and reproduction in any medium or format, as long as you give appropriate credit to the original author(s) and the source, provide a link to the Creative Commons license, and indicate if changes were made.
In cases where reviewers are anonymous, credit should be given to 'Anonymous Referee' and the source.
The images or other third party material in this Peer Review File are included in the article's Creative Commons license, unless indicated otherwise in a credit line to the material. If material is not included in the article's Creative Commons license and your intended use is not permitted by statutory regulation or exceeds the permitted use, you will need to obtain permission directly from the copyright holder.
To view a copy of this license, visit https://creativecommons.org/licenses/by/4.0/

Response to reviewers

Reviewer 1

The manuscript by Iftakher et al. presents a hybrid hardware Ising machine that integrates hafnium-oxide memristor crossbar arrays with stochastic magnetic tunnel junctions (SMTJs) to achieve intrinsic voltage-controlled annealing. The memristor crossbar array is used for MAC operation, while sMTJ is used for stochastic switching. Both parts have been well studied before. While combining them might indeed bring some benefits, the overall approach is relatively straightforward. The experimental demonstrations on MAX-CUT and graph coloring problems are encouraging, but several critical issues need to be addressed before recommending this work for publication in Nature Communications.

We thank Reviewer 1 for their thorough and detailed review. We appreciate the recognition of our experimental demonstrations combining memristive crossbar arrays and stochastic magnetic tunnel junctions, and we acknowledge the reviewer's thoughtful comments regarding integration, scalability, and system-level benchmarking. We have revised the manuscript accordingly and provided detailed responses to each point below.

1. First, the authors claim that ADC is not needed in this system, which would be a significant advantage. However, has the analog connection between memristor crossbar array and SMTJ been experimentally demonstrated? The current setup appears to use separate dies with PCB connections and lab equipment in between. If this direct analog coupling has been achieved, the authors should provide detailed characterization. If not, this should be experimentally realized as it represents the core innovation of this paper.

We thank the reviewer for this important question. It highlights the importance of experimentally demonstrating the analog connection between the memristor crossbar and the SMTJ, to support our claim that no ADC is required on the critical computation path.

In the revised manuscript, we have added a new dedicated experiment and a new main-text figure (Fig. 2, reproduced below) that explicitly demonstrates this full analog coupling and that it supports intrinsic annealing by modulating the memristor read voltage. First, the CMOS-integrated memristor crossbar is physically connected to an SMTJ through an operational amplifier wired as a transimpedance (current-to-voltage) converter, which implements in hardware the mapping

$$V_{\mathrm{mtj}} = V_{0.5} + I_{\mathrm{MAC}} R_{\alpha}$$

described in the Methods. No analog-to-digital conversion is used anywhere along this path.

Second, we program a set of memristors to two conductance levels (99 and 66 µS), apply six representative spin configurations to the crossbar, and measure the SMTJ antiparallel probability P_{AP} as a function of the effective V_{mtj} for three read amplitudes $V_{\mathrm{read}} = 20, 50, 100$ mV. As shown in Fig. 2b, the same set of spin configurations produces different I_{MAC} and thus different V_{mtj} as V_{read} is increased, and the measured P_{AP} points move along the SMTJ's sigmoid from a highly stochastic regime (probabilities near 0.5 at 20 mV) to almost deterministic plateaus (probabilities near 0 or 1 at 100 mV). This directly demonstrates that a single global analog knob, V_{read}, controls the effective inverse temperature β in the Gibbs sampler, and that annealing is achieved purely through the analog coupling between the crossbar and the SMTJ.

To avoid confusion, we now make the following clarifications in the Results, Methods, and Supplementary Information:

- We explicitly state that the intrinsic annealing mechanism is realized with a fully analog chain from the memristor MAC current to the SMTJ input voltage, without any ADC in the memristor-to-spin path.
- We clarify that the computer-in-the-loop configuration used for the longer MAX-CUT and graph-coloring experiments was chosen only for flexibility (complete waveform logging and safe voltage compliance for a first proof-of-concept), and not because an ADC is required by the architecture.
- Supplementary Note 2 and Supplementary Figs. 5 and 6 now detail both the purely analog-coupled experiment and the benchtop setup, clearly distinguishing the architectural requirement (no ADC on the compute path) from the convenience of the measurement infrastructure.

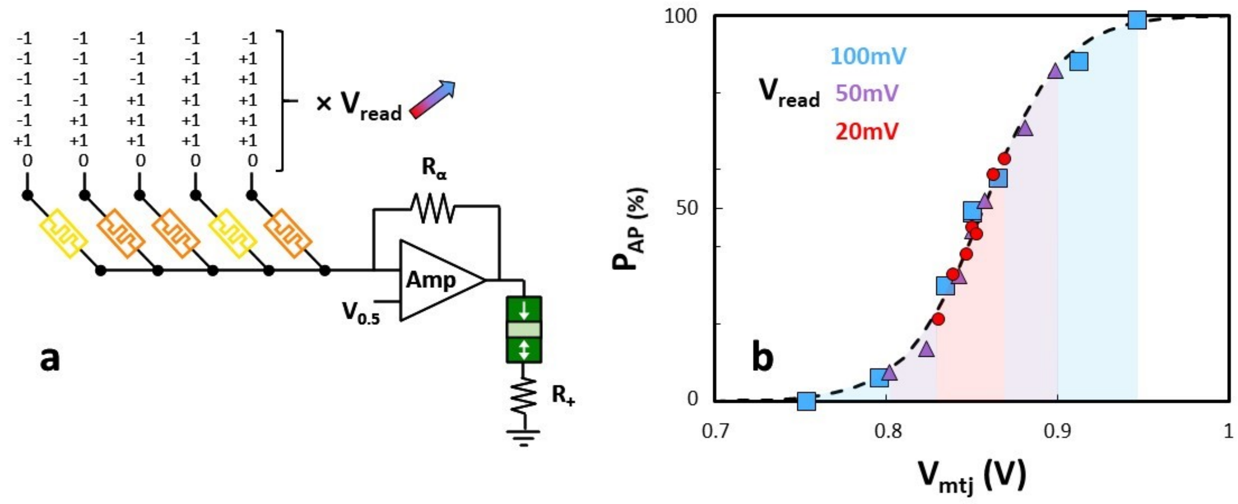

Figure 2. Principle enabling intrinsic annealing demonstrated with full analog coupling. a Schematic of the intrinsic annealing experiment. A partial row of memristors from the memristor array (Fig. 1b), programmed with the color-coded conductances (yellow: 99 µS, orange: 66 µS), are activated with spin inputs, applied as small offsets around a common reference level $V_{0.5}$ (spin × V_{read}). An operational amplifier wired as current-to-voltage converter biases the SMTJ, and the resulting probability P_{AP} is obtained from time traces of the current through the load resistor R_+ (see Methods). $V_{0.5}$ is the voltage at which the SMTJ states are equiprobable. **b** Measured probability of the anti-parallel state P_{AP} as a function of the effective MTJ voltage V_{mtj}, inferred from the memristor array (I_{MAC}) and the feedback resistor R_α. Data are shown for different read voltages: lower V_{read} values produce a more stochastic response, whereas higher V_{read} values drive the system toward deterministic plateaus. Thus, V_{read} acts as an inverse pseudo-temperature and allows intrinsic annealing. The dashed line is a sigmoidal fit to guide the eye.

2. The annealing method used in the paper tuned the V_{read} applied to the array. However, changing V_{read} might also have an impact on the signal-to-noise ratio (SNR). Will this effect influence the system performance? The authors should plot how the SNR changes with different V_{read} (similar plots with Fig. 2b but under different V_{read}). This is crucial because if SNR degrades significantly at low V_{read} (high temperature regime), it could compromise the exploration phase of annealing, while poor SNR at high V_{read} could affect the final solution quality.

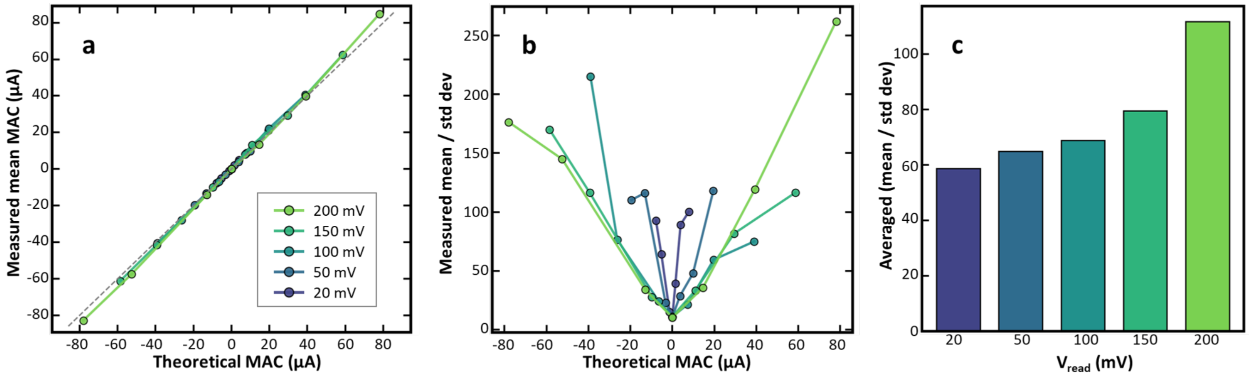

Supplementary Figure 7. Read-voltage dependence of MAC accuracy and noise. a Measured multiply-accumulate (MAC) currents compared with their ideal values for several read voltages V_{read}. **b** Effective SNR (μ/σ) extracted for each input-conductance combination and plotted against the corresponding ideal MAC value, illustrating how signal quality varies both with MAC magnitude and with V_{read}. **c** Average μ/σ for each V_{read}, providing a summary of the overall SNR behaviour as the read voltage is varied.

We thank the Reviewer for pointing out this important aspect. We have now characterized how the MAC signal-to-noise ratio depends on V_{read} and added a new Supplementary Note 4 and Supplementary Fig. 7 (reproduced here).

Concretely, we measured the MAC current for many input-conductance combinations and several values of V_{read}. For each combination, we extracted the mean MAC current μ and its standard deviation σ, and used μ/σ as an effective SNR. Supplementary Fig. 7a shows that the mean MAC response follows the ideal linear behaviour for all V_{read} values considered. Supplementary Fig. 7b displays μ/σ as a function of the ideal MAC current, and Supplementary Fig. 7c summarizes the average μ/σ for each V_{read}, given the same input-conductance combinations. The main conclusions are:

- As expected for an analog MAC, the SNR increases with V_{read}: at low V_{read} the absolute currents are small, so relative noise is larger; at higher V_{read} the signal grows faster than the noise and μ/σ improves.
- For the range of V_{read} used in the experiments (20-250 mV), the SNR remains high enough that the MAC non-idealities do not dominate the dynamics.
- The more noisy regime at low V_{read} actually coincides with the initial, high-temperature phase of the anneal, where the dynamics are intentionally stochastic and small inaccuracies in evaluating the local field f_i are acceptable (and can even enhance exploration). As V_{read} increases, both the pseudo-temperature decreases and, at the same time, the SNR increases, so the system enters a progressively more deterministic regime.

In a finalized integrated system, the effective SNR might be further degraded compared to our benchtop experiments, because fluctuations of the read amplitude V_{read} can arise from supply noise or shared-bias distribution. To address this point, we presented simulations in the new Supplementary Note 1 that include such V_{read} noise on larger MAX-CUT instances than those solved experimentally (Suppl. Figs. 2 and 4). For dense 100-node graphs, we find that such noise smooths the sigmoid. In the noise-free case, the nominal annealing schedule yields a 96% success rate. Introducing a 15 mV standard deviation on V_{read} reduces this to 68%. A simple compensation recovers the performance: by uniformly rescaling the pseudo-temperatures according to the ratio of slopes between the zero-noise and $\sigma_V = 15\,\text{mV}$ conditions, the success rate is restored

to 95%, indicating that the impact of V_{read} noise can be largely mitigated by an adjustment of the annealing schedule.

3. As noted, the demonstrated problems are sparse (85.4% and 82% zero couplings). Many real-world optimization problems have dense connectivity. Is there any limitation for the system with the problem density? If no, could the author demonstrate a denser problem?

4. The demonstrated problems are relatively small (24-node MAX-CUT and 10-node graph coloring). For a technology claiming to offer "a scalable pathway toward compact, fast, and energy-efficient large-scale Ising solvers,". Could the authors demonstrate the approach on larger problem instances, or at least provide a clear analysis of what limits the current implementation from scaling?

We thank the reviewer for these closely related comments on sparsity and problem size, which both touch on the question of scalability.

To address the reviewer's concern, we have added a scalability study in a new Supplementary Note 1, where we simulate our architecture on larger and denser weighted MAX-CUT instances, including the BiqMac instance *pw09* (100 nodes, 90% density, 10 weight levels) and additional `rudy`-generated graphs. In these simulations, we explicitly inject device variability (memristors and SMTJ) and circuit noise that we saw in our experiments. We show that the solver maintains high success rates on these dense 100-node instances for realistic levels of memristor programming dispersion, SMTJ parameter spread, and V_{read} noise (see Supplementary Figs. 1-4), indicating that density does not introduce a qualitative limitation and that our proposal is not limited to the instance size that we demonstrated experimentally.

Overall, we find that the largest challenges when scaling our approach are memristor variability, and one particular type of variation of the SMTJ (see the new Suppl. Fig. 4, reproduced here). We outline straightforward mitigation strategies for these two issues (tighter program-and-verify, multi-cell weights for memristors, and per-device $V_{0.5}$ calibration for the SMTJ). We now very explicitly discuss these points in the Discussion of the main article.

We have also expanded the Discussion and added a dedicated scalability section (Supplementary Note 6) to clarify what currently limits experimental size and how the architecture would scale in a realistic fully integrated implementation. In particular:

- The present prototype is update-rate-limited by instrumentation ($\sim 50\ \mu$s pulses on a single SMTJ). Using fast SMTJs with nanosecond-scale stochastic switching (which we now demonstrate experimentally, see next question), and on-chip peripheral circuitry, would push the intrinsic p-bit update rate in the nanosecond regime and enable parallel updates across many spins.

- The memristor MAC itself does not require an ADC, so the energy and latency per update scale primarily with array size, line resistance, and device conductance range. We discuss how tiling moderate-size crossbars and using 2.5D / 3D integration (interposers, hybrid bonding, monolithic BEOL stacking) can support much larger graphs with short interconnects (main Discussion and Supplementary Note 6).

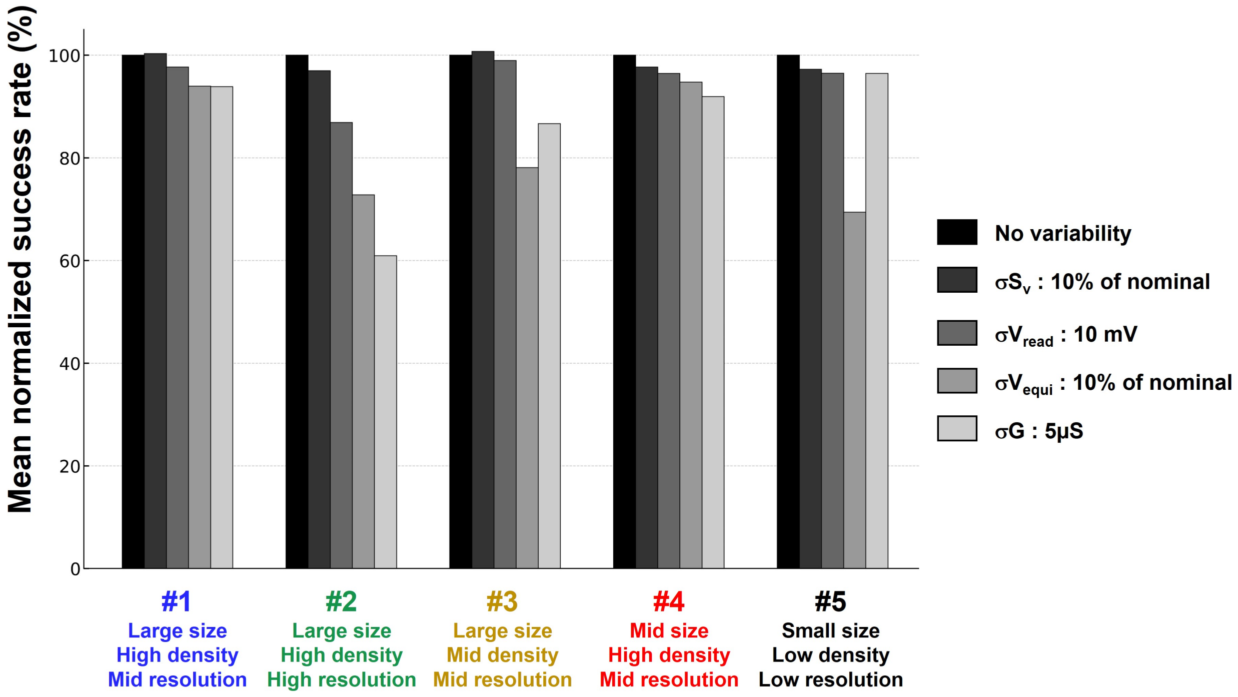

Supplementary Figure 4. Impact of device variability sources on solver performance for weighted MAX-CUT instances. Mean success rate normalized to the nominal (no-variability) case for five representative problem instances (#1–#5), characterized by different graph sizes, densities, and weight resolutions (as indicated below each group). Each bar shows the impact of a specific variability source: slope factor variability $\sigma_{S_v} = 10\%$ of the nominal value, read voltage noise $\sigma_{V_{\text{read}}} = 10$ mV, equilibrium voltage variability $\sigma_{V_{\text{equi}}} = 10\%$ of the nominal value, and conductance variability $\sigma_G = 5\ \mu$S. The un-normalized success rate values corresponding to the black bars all lie between 90% and 100%, as shown in Suppl. Figs. 3b,c, with differences between instances partly attributable to the use of a single, common annealing schedule.

5. While the authors claim potential nanosecond operation and energy efficiency advantages, no actual timing or energy measurements are provided. The current implementation uses sequential Gibbs sampling with 50 µs pulses, which seems far from the claimed nanosecond capability. What is the actual time-to-solution and energy consumption for the demonstrated problems? How does this compare with state-of-the-art digital or other hardware Ising solvers (e.g., memristor+CMOS, CMOS+sMTJ)?

We thank the reviewer for this important point. In the revised manuscript and in the new Supplementary Note 6, we now clearly separate (i) the measured behaviour of our current benchtop prototype from (ii) the projected performance of a fully integrated, nanosecond-class implementation, and we explicitly report time-to-solution and energy estimates.

1. Time-to-solution.

In the present experiments, all spins are updated sequentially using 50 µs read pulses. This choice is intentionally conservative and dictated by practical considerations: (i) accommodating the spread in switching times of the academic SMTJ process used here, and (ii) staying comfortably within the bandwidth and compliance limits of the external instruments and PCB wiring. It is not a fundamental limitation of either the SMTJs or the memristor array.

For the 24-node weighted MAX-CUT instance, we perform 300 full sweeps of the 24 spins, i.e., 7,200

sequential spin updates. The intrinsic pulse-level time-to-solution of the hardware loop is therefore $7{,}200 \times 50\ \mu\text{s} \approx 0.36$ s. For the *10-node, 3-color graph-coloring* instance, we perform 50 sweeps of the 30 spins, i.e., 1 500 updates, corresponding to $1{,}500 \times 50\ \mu\text{s} \approx 0.075$ s. These numbers are now stated explicitly in the Discussion of the revised article. We also clarify that the wall-clock runtime of the computer-in-the-loop setup (Python orchestration, inter-instrument communication) is much longer, but this overhead is purely due to external instrumentation.

In an integrated system, the time-to-solution can be reduced by orders of magnitude for two reasons. First, although our prototype uses standard Gibbs sampling (one spin updated at a time), the memristor array already computes the local fields of many spins in parallel. Realizing physical parallelism, therefore, only requires providing multiple p-bits that can switch concurrently. SMTJs are particularly well suited for this, since a single junction plus a minimal selector forms one p-bit, whereas CMOS-only p-bit circuits require thousands of transistors of overhead[1]. This opens the door to massively parallel sampling schemes such as chromatic Gibbs sampling based on graph coloring[2,3], replica-based tempering[4,5], or fully asynchronous dynamics[6–8], which have been shown to accelerate convergence significantly.

Second, the relevant devices can in principle operate at nanosecond timescales. Our CMOS-integrated SMTJs already enable p-bit sampling down to about 130 ns in the same technology node. In addition, we have non-integrated SMTJs in our laboratory that switch in the nanosecond regime (others have reported equally fast SMTJs). Measurements on such devices from our lab are now shown in Suppl. Fig. 10. CMOS-integrating these faster junctions is a technical, not a conceptual, challenge. The memristor crossbars used to implement the spin-spin couplings can also operate at nanosecond read times, and, crucially, they do not require any ADC at their outputs in our architecture: the MAC current directly biases the SMTJ. Together, these considerations support the claim that a parallel, integrated implementation of our scheme could achieve nanosecond-scale spin updates. Previous studies by the Camsari group at UCSB have shown that being able to sample spins in parallel, at nanosecond timescales, can reduce time to solution by several orders of magnitude compared with conventional electronic implementations[1,2].

2. Energy consumption and comparison to other Ising solvers.

The absolute energy consumption of the present benchtop experiment is dominated by lab instruments and long PCB traces and is therefore not meaningful for comparison with integrated Ising solvers. Instead, in the Discussion of the revised manuscript, we provide order-of-magnitude estimates for a realistic integrated system and discuss how our architecture compares qualitatively to existing approaches.

In our scheme, the dominant energy contributions in an integrated implementation are:

1. the energy dissipated in the p-bit circuits (including the SMTJ),
2. the energy dissipated in the memristor array during the MAC operation, and
3. the energy of the simple analog front-end (op-amp-based circuit) that converts the MAC current into the voltage applied to the SMTJ (Fig. 2a, main text).

For (1), prior work from the Camsari group has shown that, with nanosecond-class SMTJs, p-bit sampling energy can be pushed down to the femtojoule-per-bit range[1]. For (2), the absence of an ADC at the array output is a key advantage of our architecture: in standard memristor-based accelerators, the MAC energy is often dominated by the ADC, whereas here the MAC current directly biases the SMTJ. For representative parameters (10 ns read time, device resistance on the order of 50 kΩ and moderate read voltages), the energy per device read is on the order of a few femtojoules, i.e., $\sim$ fJ $\times N$ per p-bit for an $N \times N$ problem. For (3), simple sizing of an op-amp in a commercial 22 nm CMOS process suggests that, for dense arrays, the

front-end energy can be made comparable to the energy dissipated in the memristor array itself. These are deliberately conservative, order-of-magnitude estimates, but they are sufficient to highlight the potential efficiency of the proposed architecture.

Qualitatively, this leads to the following comparison with state-of-the-art Ising solvers:

- Pure CMOS approaches incur high energy and area costs in both MAC operations and random-number generation (RNG)[1].
- Hybrid CMOS+SMTJ approaches reduce the RNG cost, but still rely on energy-intensive CMOS MACs and data movement between memory and compute[6,8–10].
- Memristor- or FeFET-based Ising machines reduce the MAC energy by computing in memory, but typically require both high-resolution ADCs at the array output and additional RNG circuitry[11–15].
- Our hybrid memristor+SMTJ architecture simultaneously removes the ADC overhead and embeds the stochasticity directly in the non-volatile p-bit devices at the periphery of the array, thereby reducing both MAC and RNG costs in principle.

Given the diversity of problem sizes, encodings, and reporting methodologies in the current literature on Ising machines, direct, fair energy-per-solution comparisons remain difficult. As discussed, for instance, at the 2025 International Workshop on Ising Machines in Chicago, the community is moving toward more standardized benchmarks, but this effort is still ongoing. For this reason, and to avoid over-claiming, we chose to present our energy analysis in the revised Discussion and Supplementary Note 6 as a qualitative, architecture-level comparison supported by conservative order-of-magnitude estimates, rather than as precise numerical speed-up factors with respect to specific prior implementations.

6. The manuscript mentions "significant die-to-die variability" in SMTJs but doesn't analyze how this impacts system performance. In a scaled system with thousands of SMTJs, how would such variability affect solution quality and convergence? Similarly, what about memristor conductance variations and drift over time?

We thank the reviewer for highlighting the crucial question of device variability and its impact on large-scale behavior. In the revised manuscript, we now address both SMTJ and memristor variability in detail, combining new simulations with additional experimental data.

First, we have added a new Supplementary Note 1: Variability and Scalability, where we explicitly study how different variability sources affect solution quality and convergence on larger, denser weighted MAX-CUT instances, including the BiqMac benchmark *pw09* (100 nodes, 90% density, 10 weight levels) and several additional graphs generated with `rudy`. These simulations use experimentally calibrated models: the memristor conductance statistics and the voltage-probability sigmoid of the SMTJs are taken from measurements, and we inject variability and noise in a way that matches the hardware.

For SMTJs, we distinguish two main device-to-device variations: (i) shifts of the equiprobable point V_{equi} (offset), and (ii) changes in the sigmoid steepness S_v (slope). As shown in Supplementary Fig. 3, success rate is largely insensitive to realistic slope variations (up to 10% standard deviation), because late-stage updates occur on the deterministic plateaus of the sigmoid. In contrast, offset variability has a more noticeable impact, since it effectively introduces a device-specific bias on the local field. However, this effect is straightforward to mitigate in a scaled system: a simple per-device or per-row calibration of $V_{0.5}$ (the voltage at which $P_{\mathrm{AP}} = 0.5$)

recenters each sigmoid and restores the nominal mapping. We now state this explicitly in the Discussion and in Supplementary Note 1.

We also directly probe SMTJ temporal stability in Supplementary Note 5. Supplementary Fig. 8 shows that the measured sigmoid of a representative SMTJ, re-characterized after five days, exhibits a shift of less than 2 mV in its equiprobable point—well within the tolerance of the annealing schedule and easily compensated by the same $V_{0.5}$ calibration.

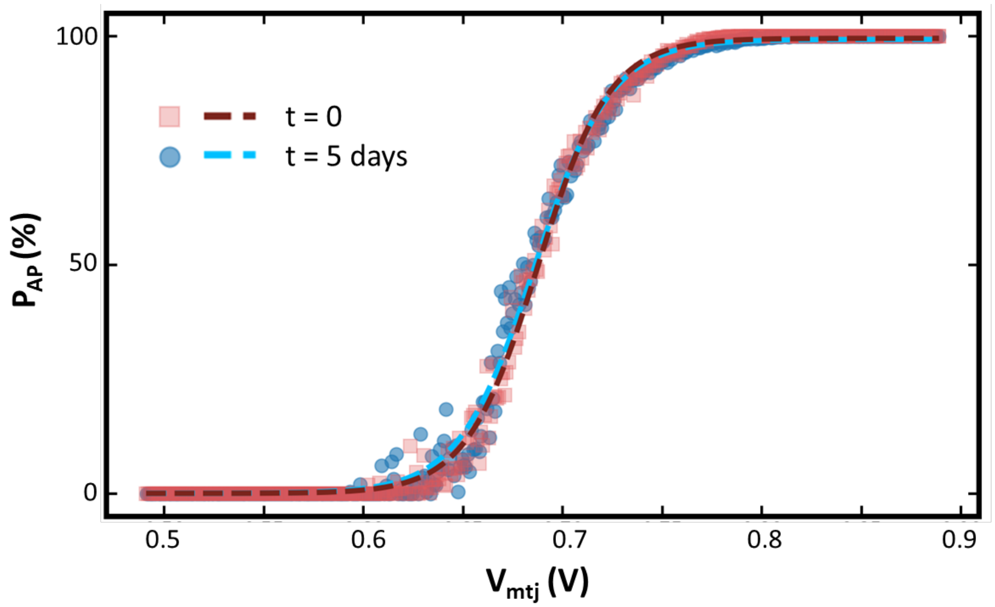

Supplementary Figure 8. SMTJ drift experiment. Switching probability of the SMTJ as a function of applied voltage (V_{mtj}). Red squares correspond to measurements at $t = 0$, while blue circles correspond to measurements after 5 days. Both datasets are fitted with sigmoidal functions (dark-red and cyan dashed lines), showing a negligible shift (< 2 mV) in the interpolated equiprobable bias point over time.

For memristors, we consider both programming dispersion and long-term drift. Programming variability is modeled as Gaussian conductance noise with standard deviation $\sigma_G \in [0, 5]$ μS, comparable to or slightly exceeding our measured dispersion (main Fig. 3a). Supplementary Fig. 1 shows that, for dense 100-node instances, the success rate remains high for realistic σ_G values, especially when the problem uses a limited number of weight levels (e.g., 5 levels). Variability becomes more critical when very fine weight resolution is required, because it can blur the distinction between nearby levels; this is reflected in the comparison between five-level and ten-level problems in the summary histogram of Supplementary Fig. 4. We discuss in the revised Discussion that higher-precision analog devices or multi-cell weight encoding (already standard in analog neural accelerators) can provide the necessary margin for such demanding instances.

Regarding drift, the main text reports a one-month retention experiment on the graph-coloring benchmark (Fig. 5e,f): the couplings and biases drift by less than ~ 15 μS over 10^3 h at room temperature with RESET-based programming, and the same memristor array, re-used without reprogramming, still drives the solver to the optimal solution with essentially identical convergence traces. This demonstrates that the observed drift is modest relative to the conductance spacing and does not degrade solution quality for the considered task.

Finally, Supplementary Fig. 2 analyzes the impact of V_{read} noise (modeled as Gaussian with $\sigma_{V_{\mathrm{read}}} = 10$-20 mV) on the effective SMTJ response. For dense 100-node graphs, such noise slightly smooths the sigmoid but leads only to a small reduction in success rate; in practice, it behaves like a modest temperature jitter

that can be compensated by a slightly deeper or slower final annealing stage.

Altogether, the new simulations and experiments show that: (i) SMTJ slope variability is benign, SMTJ offset variability can be compensated by simple $V_{0.5}$ calibration, and SMTJ drift is small; (ii) memristor programming dispersion at the level of our current hardware has a limited impact on convergence for realistic weight alphabets and can be further mitigated with improved programming schemes or multi-cell weights; and (iii) long-term memristor drift and moderate V_{read} noise do not prevent the solver from reaching optimal solutions on month timescales.

We have updated the Discussion and added cross-references to Supplementary Note 1 (variability analysis) and Supplementary Note 5 (SMTJ stability) to make these conclusions more explicit and to clarify how variability would be handled in a scaled system with thousands of SMTJs and large memristor arrays.

Reviewer 2

The manuscript presents a novel hardware-based approach to solving combinatorial optimization problems. The authors propose an Ising machine architecture that integrates two distinct nanodevices: a memristor array for multilevel coupling and bias storage, and superparamagnetic magnetic tunnel junctions (sMTJs) functioning as probabilistic bits (p-bits) to enable stochastic computation. Notably, the annealing parameter is internally controlled by tuning the voltage applied to the sMTJs, which is directly derived from the current outputs of multiply-accumulate (MAC) operations performed by the memristor crossbar array. The system is demonstrated on small-scale optimization tasks, including a 24-node Maxcut problem and a 10-node graph coloring problem with bias constraints. The work is original and provides meaningful insights into hybrid memory-based Ising machines. However, the authors need to satisfactorily address the questions and suggested improvements outlined below before the work can be reevaluated for publication.

We thank Reviewer 2 for their positive and comprehensive evaluation of our work. We appreciate the acknowledgment of the originality of our approach and the valuable suggestions to clarify system architecture, scalability, and energy-performance considerations. These comments have been carefully addressed through expanded discussion, additional data, and schematic updates in the revised manuscript.

1. While the authors successfully demonstrate the solution of small-scale optimization problems, it remains unclear how the proposed system would scale to larger problem sizes. The performance should be evaluated as a function of problem size or complexity to assess scalability. In addition, the authors should test on more complex or standardized benchmark problems and compare their method with existing solvers. Specifically, how would the architecture perform when scaled to accommodate a significantly higher number of nodes or p-bits?

Given practical limitations in memristor crossbar array sizes (typically projected to be on the order of 128×128 or 256×256), the authors can provide projections on the scalability and architectural feasibility for larger problems (e.g., Maxcut instances with 100 nodes or more). At such scales, would the intrinsic annealing approach via voltage modulation based on crossbar MAC outputs remain effective?

We thank the reviewer for raising the crucial issue of scalability and benchmarking. Our original submission focused on a proof-of-concept hardware demonstration with conservative, instrument-limited experiments. In the revised manuscript, we now make our scalability claims more quantitative by (i) adding a dedicated variability-and-scalability simulation-based study on larger, denser instances (new Suppl. Note 1), and (ii) clarifying in a new Suppl Note 6 and in the Discussion how a parallel, integrated version of the architecture would scale in practice.

First, we have introduced a new Supplementary Note 1: Variability and Scalability, where we simulate our hybrid memristor-SMTJ Ising machine on larger weighted MAX-CUT problems, using experimentally calibrated device models. In particular, we include the standardized benchmark BiqMac instance *pw09* (100 nodes, 90% density, 10 weight levels) and several additional graphs generated with `rudy`, spanning different densities and weight alphabets. The MAC operation, SMTJ sigmoid dependence, and noise sources are all parameterized from measurements. We report success rates and cost-versus-iteration traces under realistic memristor programming dispersion, SMTJ parameter spread, and V_{read} noise. These results show that the architecture retains high success probability on dense 100-node instances and that performance is primarily limited by memristor conductance dispersion when very fine weight resolution is required and a particular

type of SMTJ imperfection. We summarize these trends across instances in Supplementary Fig.4. We outline straightforward mitigation strategies for these two issues (tighter program-and-verify, multi-cell weights for memristors, and per-device $V_{0.5}$ calibration for the SMTJ).

Second, we have expanded the Discussion and added a new Supplementary Note 6: Design and device assumptions for projections to clarify how the architecture scales with the number of spins/p-bits in an integrated implementation. The key points are:

- In the present demonstrator, a single SMTJ is time-division-multiplexed across all spins, with ~ 50 μs pulses enforced by the characterization instrument. As a result, wall-clock time grows linearly with the number of spins, and this is the main reason we restricted the hardware experiments to 24 and 30 spins. In a scaled system, each row would be equipped with its own SMTJ and a simple pre-amplifier/comparator chain (illustrated in Supplementary Fig. 9), allowing fully parallel updates across N spins.

- Fast SMTJs with nanosecond-scale stochastic switching at femtojoule energies have already been demonstrated in non-CMOS processes (we now show some from our lab in the new Suppl. Fig. 10). Coupled with the fact that our memristor MAC does not require an ADC, this implies that a realistic integrated system could, in principle, operate with nanosecond-scale update times per spin and exploit parallel Gibbs-sampling variants (chromatic updates, tempering, asynchronous dynamics), which we now discuss explicitly.

- We also outline a concrete integration roadmap (2.5D interposers, 3D hybrid bonding, and monolithic BEOL stacking), explaining how it removes centimeter-scale PCB traces and bench instruments that currently dominate latency and energy in our setup. This progression shrinks interconnects from millimeters to sub-micrometers, bringing the effective time-to-solution closer to the intrinsic nanosecond dynamics of SMTJs.

In this work, our primary goal in this work is to demonstrate a new device-level mechanism (intrinsic annealing via a single shared V_{read}) and a hybrid architecture that unifies couplings, spins, and annealing in hardware. The focus is therefore on validating the physical principles, not on outperforming advanced classical software or specialized architectures. Table 1 situates our approach within the landscape of existing nanotechnology-based Ising machines, highlighting that most prior demonstrations rely on CMOS-emulated spins or digitally implemented couplings. The additional simulations on 100-node BiqMac-style benchmarks in Supplementary Note 1 show that the proposed architecture maintains high success probability under realistic device variability, supporting the scalability of the underlying mechanism without relying on assumptions specific to our small-scale hardware experiments.

2. There is no discussion on energy consumption, speed, or area of the proposed system. Nor is there a comparison with other state-of-the-art Ising solvers. Without these key metrics, it is difficult to evaluate the practical advantages of the approach. A quantitative analysis of the energy consumption and performance characteristics of the proposed hybrid system would be beneficial. Estimation of the energy required to solve the optimization problems presented and how power and latency are influenced by the interaction between the memristor crossbar array and the sMTJs.

This comment closely overlaps with Reviewer 1, Question 5. For completeness, we summarize here the main points of our response; further details are provided in our answer to Reviewer 1, Question 5.

In the revised manuscript, we now clearly distinguish (i) the measured behaviour of our benchtop prototype from (ii) the projected performance of a fully integrated implementation described in the new Supplementary Note 6, and we provide explicit time-to-solution and energy estimates.

Speed and time-to-solution. In our present instrument-in-the-loop setup, spins are updated sequentially with 50 µs read pulses. With this pulse duration, the 24-node MAX-CUT instance requires 7,200 updates (300 sweeps), corresponding to an intrinsic hardware time of ≈ 0.36 s, while the 10-node, 3-coloring instance requires 1,500 updates (50 sweeps), i.e. ≈ 0.075 s. These values are now stated explicitly in the Discussion. The long wall-clock time of the Python/instrument loop is not representative of the hardware.

In a final architecture like the one proposed in Supplementary Note 6, Supplementary Figure 9, all rows of the memristor array can be read in parallel, and each row is paired with its own SMTJ-based p-bit. This makes it possible to update many spins concurrently, e.g. with chromatic or asynchronous updates, which would further reduce time-to-solution. CMOS-integrated SMTJs in our technology node already reach ~ 130 ns sampling times. In addition, we have standalone (i.e., not CMOS-co-integrated) SMTJs in our laboratory that switch in the nanosecond regime (others have reported equally fast SMTJs). Measurements on such devices are now shown in Supplementary Note 6, Supplementary Figure 10. Because our architecture requires no ADC at the array output, spin-update times are ultimately limited by SMTJ dynamics and by the global V_{read} ramp.

Energy consumption. Absolute energy numbers from the benchtop system are dominated by SMUs and long PCB traces, so we instead provide architectural-level estimates for a realistic integrated design. The dominant contributions are: (i) SMTJ-based p-bit sampling, (ii) the memristor MAC, and (iii) a simple analog front-end converting MAC current into the SMTJ input. Prior work shows that nanosecond SMTJs can operate in the femtojoule range; memristor reads with no ADC consume only a few femtojoules per device; and the analog front-end can be designed to comparable levels in scaled CMOS. These estimates are now provided and discussed in the main text and in a dedicated section of the new Supplementary Note 6.

Area considerations. Area is dominated by the memristor crossbar itself, which naturally scales as $N \times N$. Although each SMTJ p-bit includes a small preamplifier and comparator, the overall footprint remains much lighter than CMOS-only stochastic-bit generators or the per-row ADCs typically required in analog in-memory computing.

Comparison to state-of-the-art Ising solvers. We now clarify this point in the Discussion and in Suppl. Note 6. CMOS-only Ising machines incur high energy and area for both MACs and RNG; CMOS+SMTJ architectures keep the RNG energy low but rely on CMOS MACs; and FeFET/memristor-based Ising machines reduce MAC energy but typically require ADCs and separate RNG circuitry. Our hybrid approach removes both ADC and RNG overheads by exploiting the analog MAC current and the intrinsic stochasticity of the SMTJs. Because reporting conventions differ widely across platforms, we intentionally frame these comparisons at the architectural level using conservative, order-of-magnitude estimates.

3. In addition to this, it would be useful to understand how these metrics vary with graph sparsity. While the authors mention that the test graphs used are approximately 80% sparse, a broader evaluation across different graph densities ranging from fully connected to highly sparse would help clarify how sparsity affects the hybrid system.

We agree that the impact of graph sparsity on performance and robustness is important to clarify, and we have expanded the manuscript accordingly.

To assess the effect of graph density, we now include in Supplementary Note 1 a systematic study of weighted MAX-CUT instances up to 100 nodes for several densities, including dense (90%) graphs. The instances are drawn from the BiqMac library and from `rudy`, and we use experimentally calibrated models for the memristor MAC and SMTJ sigmoid dependence, with realistic noise and variability.

Overall, instance density has little impact on our approach. The main impact is seen in the presence of V_{read} noise. In that situation, increasing density has two main effects: (i) the total conductance per row grows, which increases the gain from V_{read} to the SMTJ input and amplifies the impact of any V_{read} noise; and (ii) as a result, the effective p-bit response is slightly "flattened" (the sigmoid is modestly smoothed), which can reduce the success rate if the annealing schedule is kept fixed. However, we show that by adjusting the annealing schedule can compensate this flattening effect, and allow recovering near-equivalent success rate than in the absence of V_{read} noise.

We now make this point explicit in the Discussion: there is no intrinsic algorithmic limitation that restricts our hybrid memristor-SMTJ Ising machine to sparse graphs. Sparsity mainly reduces programming effort and dynamic power, while denser graphs require appropriately dimensioned readout circuitry and, in practice, a slightly adapted annealing schedule.

4. In the present prototype, a host computer, a microcontroller, and two Keysight B1530A SMUs sequentially (i) apply V_{read}, (ii) measure cross-bar currents, (iii) bias a single sMTJ via a probe station, and (iv) update the schedule via Python. This remains an instrument-in-the-loop demonstration. The claim of "nearly circuit-free annealing" is overstated; an integrated system that hard-wires the resistor path and voltage ramp would be more appropriate, especially given that stand-alone memristor and MTJ systems have been widely demonstrated.

We agree with the reviewer that the present prototype is an instrument-in-the-loop demonstrator, and we have clarified this explicitly in the revised manuscript. Our intent in the original wording was not to suggest that the benchtop setup contains no circuitry, but rather to emphasize that the annealing mechanism itself does not rely on per-column ADCs, complex digital control, or waveform synthesis. A single global analog voltage V_{read} controls both the MAC amplitude and the effective sampling temperature, which is the sense in which the approach is "analog-native."

To address the reviewer's comment, we implemented several changes in the revision.

- We added a new experiment and a new main-text figure (Fig. 2) demonstrating full analog coupling between the CMOS/memristor crossbar and an SMTJ. In this setup, the memristor array's output current I_{MAC} is routed through a simple transimpedance amplifier that produces the voltage $V_{0.5} + I_{\text{MAC}} R_\alpha$ directly across the SMTJ, without any ADC or digital reconstruction. Sweeping a *single* global V_{read} moves the measured SMTJ probability from stochastic to nearly deterministic, confirming experimentally that annealing is implemented through the analog hardware itself.

- We added Supplementary Note 2, which documents the experimental wiring of both the new experiment of Fig. 2, as well as for solving the MAX-CUT and graph coloring taks in Figs. 4 and 5.

In light of the reviewer's feedback, we have also replaced the phrase "nearly circuit-free annealing" in the abstract and the Discussion with the term *analog-native annealing*, to emphasize that the advantage lies in the device-level control simplicity rather than in the temporary presence or absence of laboratory instrumentation.

5. Figure 1a gives the misleading visual impression of a fully integrated on-chip loop, yet the SMUs, microcontroller, and host computer are omitted. A workflow diagram of the complete experimental path should be included.

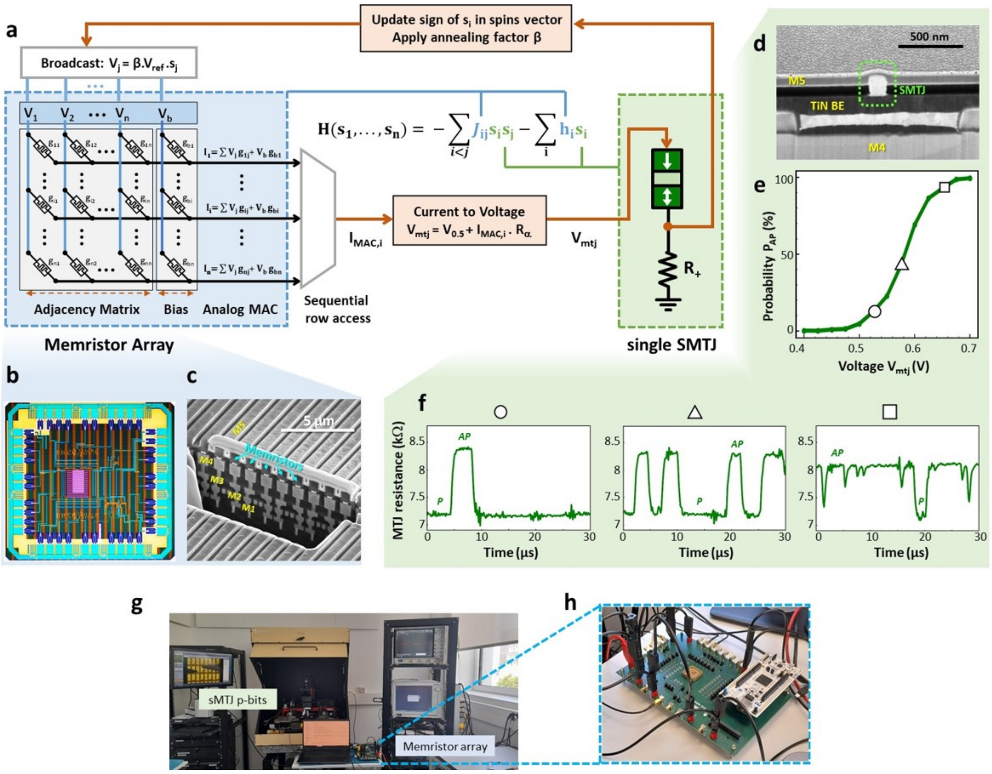

Figure 1. Hybrid memristor-SMTJ Ising-machine architecture and device primitives. a System-level concept: A hafnium-oxide memristor crossbar computes the weighted sum of neighbouring spins, while a stochastic magnetic tunnel junction (SMTJ) converts that current into a probabilistic spin update. This schematic highlights the core computational primitives of the Ising machine. The detailed experimental setups including mixed-signal circuitry and external instrumentation are presented in Supplementary Note 2. **b** Die micrograph of the 32×64 memristor crossbar integrated above CMOS. **c** Scanning Electron Microscopy (SEM) image highlighting the integration of the $TiN/HfO_2/Ti$ memristors above the fourth layer of metal interconnects. **d** SEM image showing a perpendicular SMTJ integrated in the back-end-of-line (BEOL). **e** Experimentally determined switching probability of an SMTJ versus applied voltage, showing a sigmoid dependence that implements Gibbs-sampling. **f** Sample measured time responses of the SMTJ above at V_{mtj} = 525 mV (circle), 575 mV (triangle), 650 mV (square). **g** Broader view of the experimental setup. **h** The memristor array is mounted on a custom-printed circuit board (PCB) and interfaced with a microcontroller for addressing and communication with the host computer.

We thank the reviewer for pointing out this possible source of confusion. We have made several revisions to the manuscript to make the experimental workflow explicit.

First, we have updated Fig. 1a in the main text so that it is clearly presented as a system-level concept rather than as the exact experimental implementation. The blocks in the Figure have been changed to focus on function. The caption now explicitly indicates that the memristor crossbar and SMTJ are shown as the core hardware primitives of the Ising machine.

Second, we have added a dedicated Supplementary Note 2: Experimental setups, which includes two workflow-style diagrams (new Supplementary Figs. 5 and 6). These figures detail:

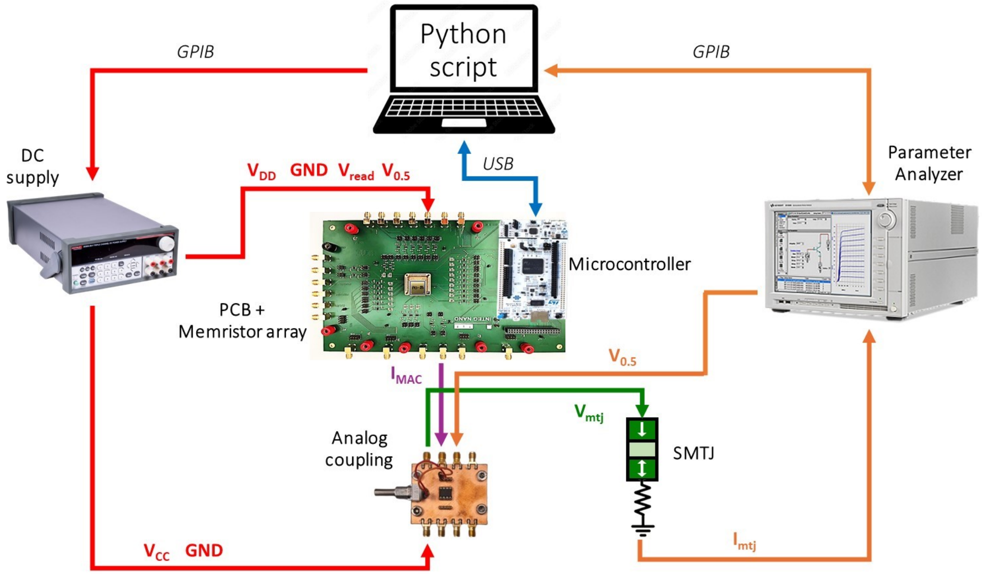

Supplementary Figure 5. Experimental configuration enabling analog coupling between the memristor array and the SMTJ. Computer-in-the-loop measurement setup used for the experiment in Fig. 2. The DC supply (Keithley 2230G) provides the array bias voltages ($V_{\rm DD}$ and $V_{\rm read}$) and the reference level ($V_{0.5}$), while the parameter analyzer (Keysight B1530A) independently delivers an identical reference voltage ($V_{0.5}$) to the operational amplifier, and measures the magnetic tunnel junction (SMTJ) current ($I_{\rm mtj}$). An MCP6002 operational amplifier, powered by an independent supply voltage ($V_{\rm CC}$), converts the array output current ($I_{\rm MAC}$) into the voltage ($V_{\rm mtj}$) driving the SMTJ. An STM32 microcontroller loads the test patterns onto the array, and instrument control and data acquisition are automated through a Python script via GPIB and USB interfaces.

- the instrument-in-the-loop configuration used to demonstrate full analog coupling between the memristor array and SMTJ (Fig. 2 of the main text), and
- the complete experimental path used for the MAX-CUT and graph-coloring demonstrations, explicitly showing the roles of the host computer, microcontroller, SMUs, probe station, and their signal flow.

In the revised Results section, we now refer readers explicitly to Supplementary Note 2 when describing the experimental setup, so that the distinction between the conceptual hardware architecture (Fig. 1a) and the practical benchtop realization (Suppl. Figs. 5 and 6) is unambiguous.

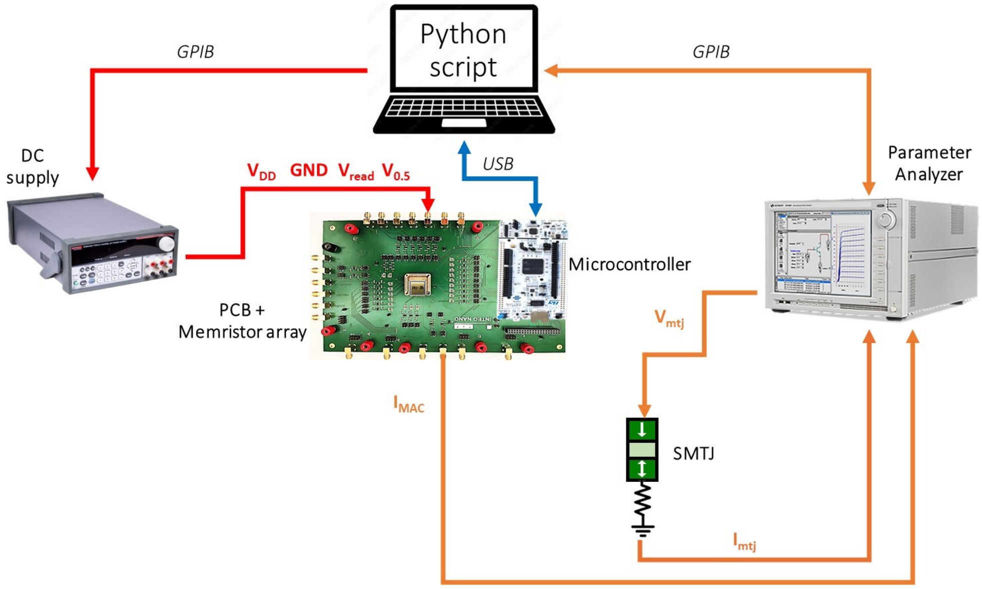

Supplementary Figure 6. Experimental configuration for computer-mediated coupling between the memristor array and the SMTJ. Computer-in-the-loop measurement setup used for the experiments in Figs. 4 and 5. The DC supply (Keithley 2230G) provides the array bias voltages (V_{DD} and V_{read}) and the reference level ($V_{0.5}$), while the parameter analyzer (Keysight B1530A) drives the magnetic tunnel junction (SMTJ) with (V_{mtj}) and measures I_{mtj}. The printed circuit board (PCB) hosts the memristor array and an STM32 microcontroller that loads the test patterns. The analog output current (I_{MAC}) from the array is sent to the B1530A, which forwards it to the control computer; the Python script then determines the corresponding V_{mtj} to be applied by the B1530A. Instrument communication and data acquisition are managed through GPIB and USB interfaces.

6. The graph coloring problem is demonstrated using a 30×30 memristor array, raising the question of whether a 30-node Maxcut problem could also be implemented to explore the upper limits of the system's problem size. The current 24-node limitation may stem from the use of three distinct multilevel coupling weights. If this is the case, the authors should explicitly discuss the tradeoffs between the number of weight levels that can be represented and the number of nodes that can be accommodated within the current hardware constraints. Clarifying these limitations would help in understanding the design space and scalability of the system. Such tradeoff analysis could complement the projections regarding system scaling.

To address the reviewer's concern, we have added a scalability study in Supplementary Note 1, where we simulate our architecture on larger and denser weighted MAX-CUT instances, including the BiqMac instance *pw09* (100 nodes, 90% density, 10 weight levels) and additional rudy-generated graphs. In these simulations, we explicitly inject device variability (memristors and SMTJ) and circuit noise that we saw in our experiments. We show that the solver maintains high success rates on these dense 100-node instances for realistic levels of memristor programming dispersion, SMTJ parameter spread, and V_{read} noise (see Supplementary Figs. 1-4), indicating that density does not introduce a qualitative limitation and that our proposal is not limited to the instance size that we demonstrated experimentally.

Overall, we find that the largest challenges when scaling toward larger and denser tasks are memristor variability, and one particular type of variation of the SMTJ. We outline straightforward mitigation strategies for these two issues (tighter program-and-verify, multi-cell weights for memristors, and per-device $V_{0.5}$ calibration for the SMTJ). We now very explicitly discuss these points in the Discussion of the main article.

We have also expanded the Discussion and added a dedicated scalability section (Supplementary Note 6) to clarify what currently limits experimental size and how the architecture scales in a realistic integrated implementation. In particular:

- The present prototype is update-rate-limited by instrumentation ($\sim 50\ \mu$s pulses on a single SMTJ). Using fast SMTJs with nanosecond-scale stochastic switching, already demonstrated in non-CMOS processes, and on-chip peripheral circuitry, would push the intrinsic p-bit update rate above 10 MHz and enable parallel updates across many spins.
- The memristor MAC itself does not require an ADC, so the energy and latency per update scale primarily with array size, line resistance, and device conductance range. We discuss how tiling moderate-size crossbars and using 2.5D / 3D integration (interposers, hybrid bonding, monolithic BEOL stacking) can support much larger graphs with short interconnects (main Discussion and Supplementary Note 6).

7. Furthermore, shedding light on programming negative weights would ascertain the flexibility for implementing various problems sets.

We agree that the handling of negative weights is central to the flexibility of the architecture, and we have clarified this point both in the main text and in a new Supplementary Note 6.

In the current experiments we use two complementary strategies, depending on whether all coefficients in a given column share the same sign:

- **MAX-CUT benchmark (main text, Fig. 4).** All non-zero couplings J_{ij} are negative. We therefore represent weights with a single memristor per edge and encode the sign through the read-voltage polarity: spins are applied as $\{-V_{\text{read}}, +V_{\text{read}}\}$, so that the resulting current contribution $g_{ij}s_jV_{\text{read}}$ has the correct sign for a negative coupling.
- **Graph-coloring benchmark (main text, Fig. 5).** The coupling matrix J is non-positive, while all on-site fields h_i are positive. We exploit the column-wise separation between J and h: all J_{ij} are read with a negative voltage ($-V_{\text{read}}$), and the bias column is read with a positive voltage ($+V_{\text{read}}$). This is the regime explicitly mentioned in the revised main text: encoding the sign in the read-voltage polarity is valid as long as all coefficients in a given column share the same sign.

For general Ising instances with mixed-sign coefficients within the same column, we now describe in detail (new Supplementary Note 6) how to implement signed weights using a standard differential scheme, as routinely employed in memristor-based neural network accelerators:

- Each logical weight J_{ij} is mapped to a pair of conductances (g^+_{ij}, g^-_{ij}), programmed such that

$$J_{ij} \propto g^+_{ij} - g^-_{ij}$$

- Two complementary input lines (or two stacked sub-arrays) carry $\pm V_{\text{read}}$; the resulting differential MAC current implements the signed local field $f_i = \sum_j J_{ij}s_j + h_i$ without requiring ADCs.
- The same principle applies to h_i if signed biases are needed, again using a pair of memristors per bias entry.

This differential encoding doubles the number of devices per logical weight (as in analog neural accelerators), but does not alter the core annealing mechanism: the MAC current is still proportional to V_{read}, and the same voltage-controlled pseudo-temperature is seen by the SMTJs.

In the revised manuscript we:

- explicitly state in the Results section how negative couplings and positive biases are currently implemented via read-voltage polarity and column separation, and
- refer to Supplementary Note 6 for the general mixed-sign case, where we spell out the differential mapping and discuss its area/programming overhead and compatibility with the proposed architecture.

8. Only three MTJs are sampled for the MAX-CUT instance; error bars across multiple devices are necessary.

We thank the reviewer for this suggestion. Simulated annealing deliberately introduces stochastic exploration, so even repeated runs on the same device follow different cost trajectories. In Figs. 4b and 5b, two of the trajectories correspond to runs on the same SMTJ, while the third comes from a second SMTJ. The spread observed across the three traces therefore reflects both intrinsic sampling noise and device-to-device variability, and in this regime the two contributions are comparable: based on the trajectories alone, one cannot tell which curve corresponds to which device. For this reason, we chose to show individual representative runs rather than error bars in Figs. 4b and 5b, and to independently assess the impact of SMTJ variability more systematically in the new Supplementary Note 1, where we inject experimentally-calibrated parameter spread into simulations of larger, denser MAX-CUT instances and report aggregate success rates (Supplementary Figs. 3 and 4). There, we find that realistic SMTJ variability has only a modest influence on performance, and that its effect can be mitigated by simple per-device $V_{0.5}$ calibration in a scaled implementation (see Reviewer 2, Question 14).

9. The known optimal solutions should be explicitly marked in Figures 3 and 4.

In the revised manuscript we now explicitly indicate the known optimal solution in the optimization plots (which are now Figs. 4 and 5).

10. One of the highlights of this work is the intrinsic annealing strategy employed by the system as mentioned by the authors. However, the manuscript lacks sufficient detail on how the annealing schedule specifically the β (inverse temperature) parameter is modulated and implemented in hardware. It is unclear whether β is controlled digitally, or if it is inherently embedded within the operation of the memristor crossbar and its associated peripheral circuitry.

We thank the reviewer for raising this question. To make the implementation of the annealing schedule fully explicit, we have revised the manuscript and added new experimental data.

In the current computer-in-the-loop setup, the annealing parameter β is defined in software and mapped to a corresponding read voltage V_{read} according to the chosen schedule. This voltage is generated by a power supply and applied globally to the crossbar. The resulting MAC current sets the SMTJ input voltage

$V_{\mathrm{MTJ}} = V_{0.5} + I_{\mathrm{MAC}} R_{\alpha}$, and the SMTJ responds through its intrinsic voltage-dependent stochastic switching. In this way, the schedule is specified digitally, but its effect is realized entirely through the analog device physics of the memristor crossbar and SMTJ.

To directly demonstrate how this analog realization operates in practice, we added a new experiment and a new main-text figure (Fig. 2). In this experiment, the memristor crossbar drives the SMTJ input entirely in the analog domain, without any analog-to-digital conversion. Varying V_{read} shifts the SMTJ's switching probability along its sigmoid response, exactly as required for implementing a decreasing effective temperature (increasing β). These measurements explicitly confirm that annealing is achieved through the analog coupling between the crossbar and the SMTJ, rather than through digital interpolation.

Finally, we clarify in the Methods ("Annealing schedule" subsection) and in Supplementary Note 6 that in an integrated version, the same control would be performed on chip using a simple low-resolution DAC. This DAC is also shown explicitly in Suppl. Fig. 9.

11. The authors briefly mention that an annealing scheduler modulates $+V_{\mathrm{Read}}$ and $-V_{\mathrm{Read}}$ via global DACs, but the description is superficial and lacks technical elaboration. It would be helpful if the authors clarified whether this modulation involves lookup tables, programmable voltage ramps, or other control logic, and how flexible or reconfigurable the annealing schedule is in practice.

In our response to the reviewer's previous question, we discussed how we implemented the annealing schedule in our experimental demonstration: in the experiment, the annealing schedule is generated in software and applied through external instrumentation. In our response to this question, we discuss how the annealing schedule can be implemented in a prospective integrated architecture. We have now included more information about this in our revised Discussion and in the the new Supplementary Note 6 (and in particular in Suppl. Fig. 9).

In the architecture shown in Suppl. Fig. 9, the effective inverse pseudo-temperature β is implemented as a global read amplitude V_{read} applied to the columns. A convenient hardware realization could be to represent β as a k-bit digital word and convert it to V_{read} using an on-chip R-2R ladder DAC. Each bit controls a switch associated with a binary-weighted branch of the ladder; with k stages, the DAC generates 2^k levels between 0 and a maximum value $V_{\mathrm{read}}^{\mathrm{max}}$, with step size

$$\Delta V_{\mathrm{read}} = \frac{V_{\mathrm{read}}^{\mathrm{max}}}{2^k}$$

A digitally defined schedule on the β word directly produces the desired $V_{\mathrm{read}}(t)$, which can be updated at every clock cycle or at a slower rate. In this scheme, the DAC provides a single global analog control, while the instantaneous spin configuration is imposed locally at the columns by digital selection of the column voltages. This separation of concerns keeps the architecture scalable: the annealing logic does not grow with problem size, while the array and column drivers scale with the number of spins.

12. The intrinsic annealing eliminates the need for ADCs between the two nanodevice-based memory modules as proposed by the authors. However, in the current experimental setup, these voltages are generated and modulated via an external instrument (B1530A), which reads currents and outputs corresponding voltages. It remains unclear how faithfully this voltage signal would be reproduced at the circuit or silicon level in a fully

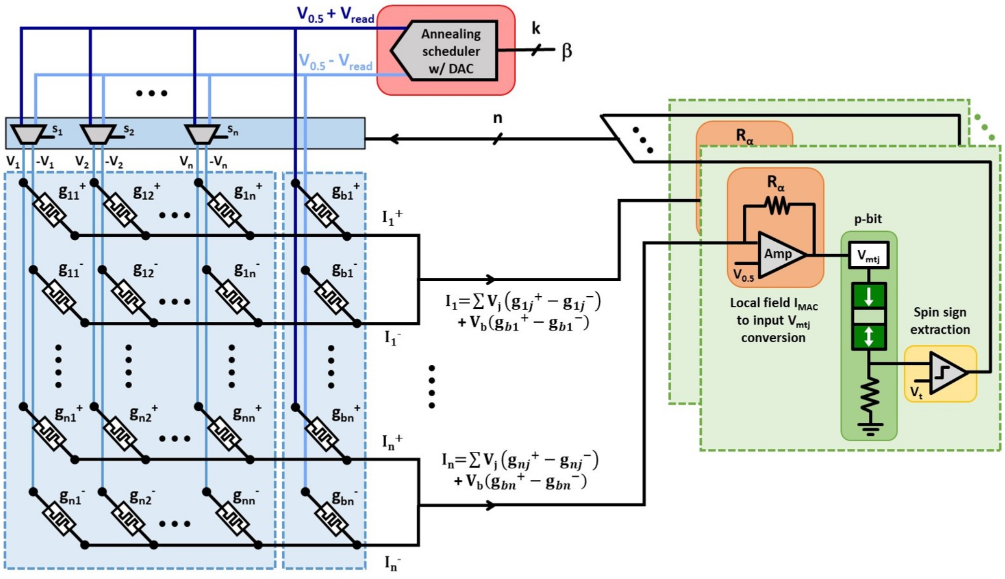

Supplementary Figure 9. Projected circuit architecture enabling parallel updates. The crossbar array (blue) stores each coupling J_{ij} in a differential memristor pair (g_{ij}^+, g_{ij}^-). For column j, the spin value s_j controls a differential driver that routes the read voltage V_{read} with the appropriate polarity, applying $(+V_j, -V_j)$ with $V_j = s_j V_{\text{read}}$ to the two columns. (In practice, these voltages are implemented as small offsets around the common reference level $V_{0.5}$, consistent with the optimization-mode operation described in the Methods.) The bias term is handled by the same differential mechanism. Each pair of rows collects the resulting currents I_i^+ and I_i^-, whose difference implements the local field. This current is converted into the SMTJ control voltage V_{mtj} by a local transimpedance stage (orange), and the SMTJ output is digitised by the comparator block (yellow) to produce the updated spin s_i. Each row includes its own amplifier, p-bit (green), and comparator, enabling fully parallel updates. When the problem is expressed in its QUBO form, only a single positive V_{read} is required, and each column driver selects between V_{read} (for $x_j = 1$) and the reference level $V_{0.5}$ (for $x_j = 0$). The annealing scheduler modulates V_{read} according to the chosen temperature schedule.

integrated system. Furthermore, as the system scales, potential variations and drops in the analog voltage path may significantly affect the fidelity of the annealing process. The authors can provide further discussion or analysis on such effects.

We thank the reviewer for highlighting this important point. To address it directly, we have added a new experiment and a new main-text figure (Fig. 2) in which the memristor crossbar drives the SMTJ input entirely in the analog domain through a simple amplifier stage, without any ADC or digital feedback (no B1530A between the two nanodevices). This measurement explicitly demonstrates that the intrinsic-annealing mechanism operates correctly when the two nanotechnologies are coupled purely via analog hardware, confirming the feasibility of the proposed ADC-free interface.

More technically: the data in the new Fig. 2 show that different spin configurations programmed into the memristor crossbar generate different MAC currents, which the analog stage converts into SMTJ input voltages. As the global read amplitude V_{read} is varied, the measured P_{AP} values move along the SMTJ's sigmoid in a manner consistent with the expected change in effective inverse temperature. This validates that annealing is implemented directly through analog coupling, and does not rely on digital interpolation from the instrumentation used for the longer MAX-CUT and graph-coloring experiments.

Regarding scaling, we agree that voltage variations and IR drops along the analog path become relevant in

larger systems, and, in a finalized integrated system, the effective SNR might be degraded compared to our benchtop experiments. To address this point, we extended the simulations in Supplementary Note 1 to include noise on V_{read} on larger MAX-CUT instances than those solved experimentally. We find that, over a broad range of realistic V_{read} fluctuations, the solver success rate is only weakly affected as long as the resulting V_{mtj} distribution remains within the sigmoid transition region. This additional analysis is now discussed in Supplementary Note 1.

We added a short paragraph in the Discussion to emphasize that the V_{read}-dependent MAC noise is not a pathological side-effect; it tracks the intended pseudo-temperature schedule, and within the measured range it does not compromise the final solution quality.

13. The authors should include brief details regarding the formation, SET, and RESET processes of the fabricated memristor bitcells used in their system. Providing representative current vs voltage (I-V) characteristics and examples of the programming pulses applied during device operation would details of the electrical behavior.

We have added a new Supplementary Note 7 ("Pulse-based programming for mapping matrices") that describes in detail the forming, SET, and RESET procedures of the 1T1R memristor bitcells used in our system, including pulse amplitudes, durations, and rise times, as well as the drift-resilient reset-based tuning scheme. In addition, we now provide representative pulsed current-voltage characteristics of devices (Suppl. Fig. 11, reproduced here) together with schematic examples of the SET and RESET programming pulses used in practice (Suppl. Fig. 12, reproduced here). These additions clarify the electrical behavior and operating conditions of the memristor bitcells.

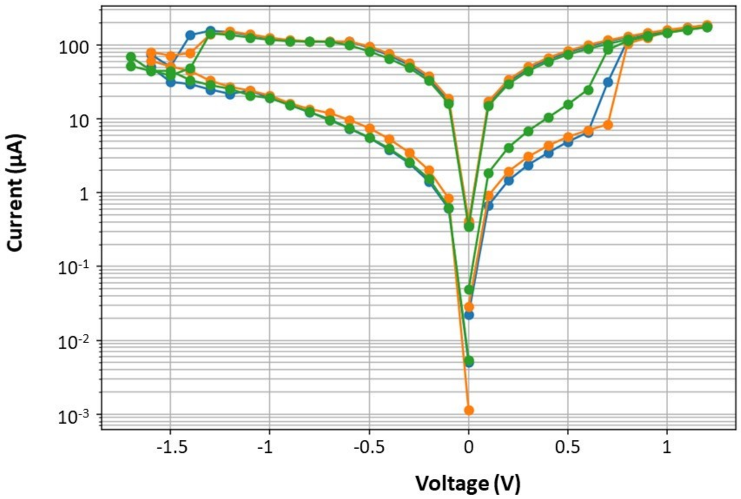

Pulsed I-V curve of three memristors in a 1T1R structure. Positive side (set process): WL voltage fixed at 2.5 V, SL at 0 V, BL sweeped back and forth from 0 V to 1.3 V at 0.1 V interval. Negative side (reset process): WL voltage fixed at 4.2 V, BL at 0 V, SL sweeped back and forth from 0 V to 1.7 V at 0.1 V interval. All pulses are 20 μs-long. The three memristors were chosen randomy in one of our memristor arrays.

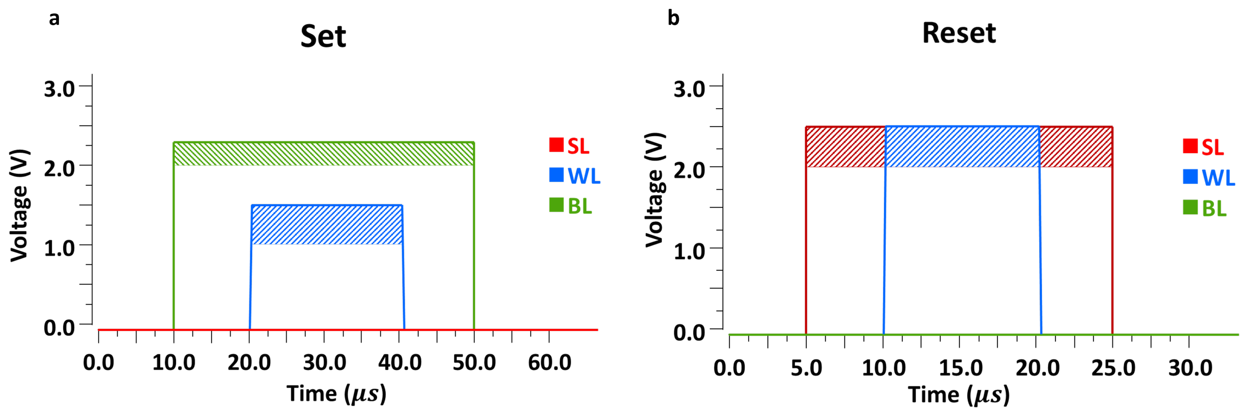

Supplementary Figure 12. Programming techniques to map the matrices. a Set-based programming technique. **b** Reset-based programming technique.

14. How many devices were fabricated and utilized in the experiments? While the authors mention executing the Maxcut problem on three different devices, it is not clear whether these devices are connected in an array formation or operated individually. If possible, the authors could expand on multiple sMTJ devices mapped to represent Ising spins, even if they are updated sequentially, to demonstrate the scalability.

On the spintronic chip, many SMTJs are available. In this work, however, we operate them one at a time on a probe station. In both Figs. 4b and 5b, two of the trajectories correspond to runs performed on the same SMTJ, while the third comes from a second SMTJ. This has now been clarified in the captions of Figs. 4 and 5 and in the main text. Because the devices are probed individually, using a probe station is convenient for this study. We have also added this clarification to the revised Methods section.

To address the reviewer's question regarding scalability and the use of multiple SMTJs, we have added a new Supplementary Note 1: Variability and Scalability, where we explicitly study how different sources of device variability affect convergence and solution quality on larger, denser weighted MAX-CUT instances. For SMTJs, we distinguish two dominant device-to-device variations: (i) shifts of the equiprobable point V_{equi} (offset), and (ii) changes in the sigmoid steepness S_v (slope).

As shown in Supplementary Fig. 3, success rate is largely insensitive to realistic slope variations (up to 10% standard deviation), because late-stage updates occur on the deterministic plateaus of the sigmoid. Offset variability has a more noticeable impact, as it introduces a device-specific bias on the local field. However, this effect is straightforward to mitigate in a scaled system: a simple per-device or per-row calibration of $V_{0.5}$ (the voltage at which $P_{\text{AP}} = 0.5$) recenters each sigmoid and restores the nominal mapping. This mitigation strategy is now mentioned explicitly in both the Discussion and Supplementary Note 1.

15. Assigning a unique R_α to each SMTJ poses significant scalability challenges. Characterizing millions of MTJs individually would be impractical. Can the authors explore the possibility of using fixed or nominal R_α values? If so, how does this simplification affect the algorithm's performance?

Using a fixed (nominal) R_α per row or per array is feasible. In practice, R_α sets the transimpedance that maps the summed current to the SMTJ input V_{mtj}, fixing it simply shifts device-to-device mismatch into the SMTJ's effective slope dispersion. This corresponds to the variability model analyzed in our response to

Question 14 by the same reviewer and in Supplementary Figs. 3 and 4, where we varied the slope parameter S_v and found that, even with 10-12.5% standard deviation, success rates remain above 90% across representative MAX-CUT instances.

Some variability in R_α itself can also be expected, but its effect is functionally equivalent to additional spread in S_v, and is therefore already captured by this analysis. In practice, per-device calibration of R_α is not required, modest global tuning of the gain or pseudo-temperature is sufficient to maintain convergence.

16. SMTJs typically have low energy barriers and are thus sensitive to temperature fluctuations. How does device heating influence experimental results and whether any thermal mitigation strategies were employed?

We appreciate the reviewer's question regarding thermal sensitivity. SMTJs are indeed thermally activated devices, and understanding how temperature affects their stochastic transfer characteristics is important. In our experiments, no dedicated thermal management was used beyond standard laboratory temperature control (23° C), but we have now carried out an explicit analysis of how temperature-induced variations would influence solver performance.

As discussed in the literature (e.g., H. Haneko *et al.*, *Appl. Phys. Express* **17**, 063001 (2024)), elevated temperature lowers the effect of the energy barrier ΔE, which broadens the sigmoid response and reduces the dwell times τ_P and τ_{AP}. In our framework, temperature changes and barrier variations enter through the slope factor S_v defined in the new Supplementary Note 1 (Eq. (6)), which we derive from the Néel–Brown expressions. Variations in T, ΔE, or V_c therefore appear as variations of S_v that modify the sigmoid steepness. Supplementary Fig. 3b examines solver robustness under realistic dispersion of S_v, covering the broadening expected from moderate temperature fluctuations. Within this range, the success rate remains essentially unchanged because late-stage updates occur on the deterministic plateaus of the sigmoid. This new analysis therefore confirms that the solver is resilient to the thermal effects that manifest as slope variations in the SMTJ response.

17. The manuscript references the presence of both slow and fast sMTJs, but does not provide sufficient detail on how these categories are defined. Are the devices classified based on the minimum pulse width required to capture a complete switching probability curve within a given voltage range?

The reviewer's intuition is correct: the distinction between slow and fast SMTJs indeed reflects differences in the minimum pulse width needed to capture a complete switching-probability curve within a given voltage range. We have clarified this point in the manuscript: fast refers to devices whose response saturates under sub-microsecond pulses, and those requiring microsecond-scale pulses are labeled slow. This clarification has been added at the end of the *Methods - Measurements of stochastic magnetic tunnel junctions* section, and a brief note has been included in the caption of Fig. 3(d,e) (formerly Fig. 2).

18. If such device-level variations exist, it raises issues with the reliability of computation in larger systems, leading to degraded solution quality when intrinsic annealing voltages are applied. The authors should discuss how these variations are managed or whether calibration per bitcell would be necessary in a scaled implementation.

This concern is addressed in our response to Reviewer 1 (Question 6) and to Question 14 by the same reviewer, where we discuss device-to-device variability and its impact on solver reliability. In practice, differences in SMTJ timescales can be managed by adapting the update pace to the slowest device, without the need for individual calibration. Since the solver operates through probabilistic exploration, the exact instantaneous value of the intrinsic annealing voltage is not critical. Determining a suitable annealing schedule is largely empirical, and small global adjustments of pseudo-temperature or update timing are sufficient to maintain convergence and solution quality.

19. The abbreviation sMTJ appears to refer to both superparamagnetic MTJ and stochastic MTJ in different parts of the manuscript. As stochastic MTJ is a more generic term, please clarify.

We thank the reviewer for catching this. The devices used here operate under bias currents of several tens of microamperes and are therefore not strictly in the superparamagnetic regime. We have revised the manuscript to consistently use the term SMTJ (stochastic magnetic tunnel junction) and removed references to superparamagnetism.

20. In Figure 1(a), the interconnections between the two memory components are not clearly illustrated. As the methods section references the use of external instrumentation these connections could be explicitly depicted in the schematic. This would help bridge the gap between the current setup and the claim of on-chip integration of the two memories.

In the revised version, we have modified Fig. 1a to make clear that it is intended as a concept-level illustration of the hybrid architecture rather than a full wiring diagram. To address the reviewer's request for explicit connections, we now provide detailed circuit schematics in the new Supplementary Note 2. There, we show how the memristor crossbar and the SMTJ are interconnected in the experimental setups used for the MAX-CUT and graph coloring demonstrations (Figs. 4 and 5), as well as in the new experiments of Fig. 2 where the two memories are directly coupled through an operational amplifier. These schematics make explicit the role of the external instrumentation in the present benchtop implementation and clarify how the demonstrated connections correspond to a straightforward path toward on-chip integration of the two nanotechnologies.

21. Fig. 1a shows n-output currents from the memristor array, yet only a single MTJ is illustrated. It confuses how these outputs are experimentally connected to a single MTJ. The authors should clarify this mechanism in the text and revise the figure accordingly.

In the revised manuscript, we have updated Fig. 1a to explicitly indicate that, in our present prototypes, the n row currents are processed sequentially and time-division-multiplexed to a single SMTJ.

22. The coupling and bias matrices used for the Maxcut and graph coloring problems are not standard benchmark problems, it would be helpful if the authors could provide the matrices either in the supplementary material or as a code/data repository link for reproducibility.

In the revised manuscript, all coupling and bias matrices used in the experiments and simulations have been compiled in the new Supplementary Note 3.

23. The inverse temperature (β) to vary linearly, V_{Read} must follow an exponential schedule. While this is visually suggested in Figure 3(b), it is not explicitly discussed in the main text.

The precise V_{Read} schedule for each task is now detailed in the Methods section of the revised manuscript.

24. In Figure 4(b), a legend can be included to indicate that the three curves correspond to independent iterations.

We thank the reviewer for the suggestion. A legend has been added to the Figure (which is now called Fig. 5) to clarify that each curve corresponds to a different run.

25. In Table 1, under the Problems Solved (size) row, it would be informative to also include the number of spins or p-bits used, coupling and bias matrix size in each implementation to have a consistent comparison across platforms, as different platforms solve various NP-hard problems.

We thank the reviewer for the suggestion. We now include in Table 1 the coupling matrix dimensions and/or the number of hardware spins (p-bits) used in each implementation, to enable a more consistent comparison across platforms.

26. In Supplementary Figure S1, the architecture diagram can include the global DACs mentioned in the main text.

In the revised manuscript, we have edited this Figure (which is now called Suppl. Fig. 9), so that it shows the global DAC explicitly.

27. Long-term stability is unsubstantiated. The MTJ is simulated for the repeat test. The authors must show repeated solve runs that include the MTJ.

SMTJs are surprisingly stable in time, even in our academic process. In the revised manuscript, we now include a dedicated Supplementary Note on SMTJ stability, mentioned in the Discussion section, where we experimentally characterize the same MTJ five days after the initial measurement. In this new analysis, we repeatedly record the stochastic transfer function, i.e., the probability of being in the antiparallel state $P_{\text{AP}}(V_{\text{mtj}})$, for identical bias conditions. We show that both the slope and offset of this sigmoid characteristic remain stable over many repetitions and over the duration of our experiments, with no systematic drift beyond the run-to-run statistical fluctuations.

We further note that, in practice, any slow drift in the SMTJ offset voltage $V_{0.5}$ (or V_{equi}) could be automatically compensated by a periodic electronic calibration of $V_{0.5}$, which is straightforward to implement and does not require reprogramming the memristor crossbar weights.

Reviewer 3

This manuscript presents an experimental demonstration of probabilistic computing using stochastic magnetic tunnel junctions (sMTJs) to solve complex combinatorial problems such as MAX-CUT and graph coloring. The authors implement single-spin Metropolis-Hastings dynamics by reusing a single sMTJ for repeated updates, and benchmark their approach on representative graph problems. The work is technically sound, and of interest to the fields of spintronics, unconventional computing, and hardware acceleration for optimization. I am generally positive about the work and recommend publication after the authors address the following points for clarification and completeness.

We thank Reviewer 3 for their positive and constructive assessment. We appreciate the recognition of the technical soundness and relevance of our demonstration to spintronics and unconventional computing, as well as the helpful remarks concerning benchmark complexity and hardware programmability, which we have addressed in detail below.

Major Comments

1. Details of the Benchmark Graphs: More detail is needed on the graphs used for the MAX-CUT and 3-coloring experiments. In particular:

- What is the average connectivity C of the graph used in the 3-coloring experiment? If C < 3, the problem can be easily solved by a greedy coloring algorithm, which would make it a relatively weak benchmark.

The 3-coloring instance corresponds to a 10-vertex graph with an average connectivity $C = 3.4$. We verified that a simple greedy coloring does not yield a correct solution for this instance. We acknowledge that the problem itself is not particularly hard; it was selected primarily to demonstrate the mixed-sign coupling configuration (even though the sign is uniform within each column) and the implementation of a bias vector within the same hardware framework.

While Supplementary Figs. 1-4 focus on graph partitioning problems, in simulation we have also successfully solved the *Les Misérables* network, which belongs to the same class of constraint-satisfaction problems as graph coloring and provides a more challenging benchmark for the solver (see Fig. R1). Each vertex represents a character from Victor Hugo's *Les Misérables*, and edges connect pairs of characters who co-occur in the same chapter, so the coloring enforces that no two characters appearing together share the same color. The corresponding instance involves a 10-coloring of a 77-vertex (770-node) graph with significantly higher average connectivity ($C = 15.6$). When applying a simulated annealing schedule consistent with the hardware implementation, the system converged to a known optimal coloring configuration.

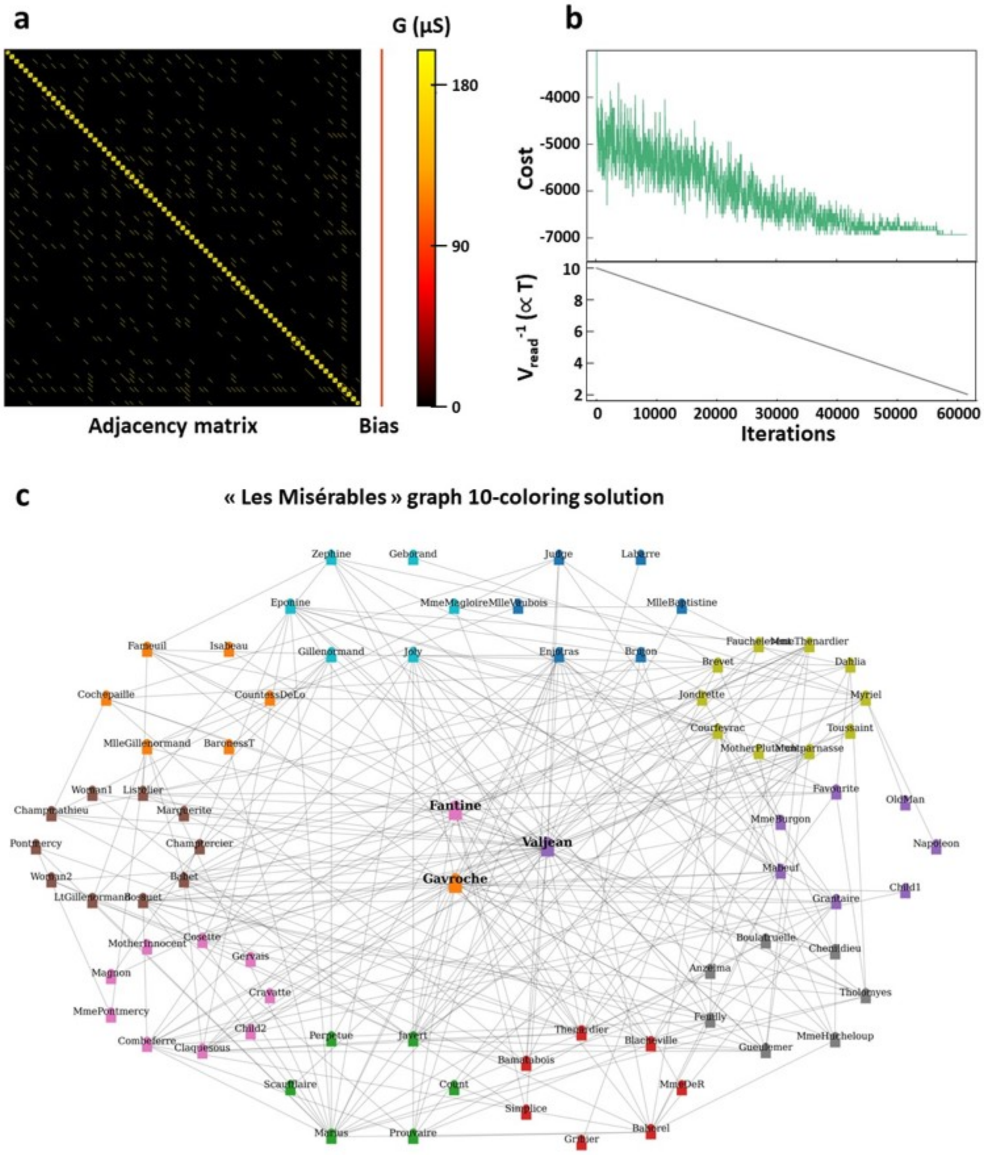

Figure R1. Graph-coloring benchmark solved with the same hardware. a One-hot encoding of a seventy-seven-node, ten-color problem known as *Les Misérables*, mapped to a sparse 770×770 conductance matrix (180 μS negative couplings) plus a 90 μS positive bias column. **b** Linear temperature schedule ($1/\beta$) applied by ramping the read voltage and evolution of Ising energy during 61600 sequential spin updates. **c** Final color configuration forming ten clusters of characters from Victor Hugo's *Les Misérables*, within which characters do not co-occur in the same chapter.

• The graphs appear visually sparse. Sparse graphs tend to be easier to solve; this should be acknowledged and discussed.

We agree that the impact of graph sparsity on performance and robustness is important to clarify, and we have expanded the manuscript accordingly.

To assess the effect of graph density, we now include in Supplementary Note 1 a systematic study of weighted MAX-CUT instances up to 100 nodes for several densities, including dense (90%) graphs. The instances are drawn from the BiqMac library and from `rudy`, and we use experimentally calibrated models for the memristor MAC and SMTJ sigmoid, with realistic noise and variability.

Overall, instance density has little impact on our approach. The main impact is seen in the presence of V_{read} noise. In that situation, increasing density has two main effects: (i) the total conductance per row grows, which increases the gain from V_{read} to the SMTJ input and amplifies the impact of any V_{read} noise; and (ii) as a result, the effective p-bit response is slightly "flattened" (the sigmoid is modestly smoothed), which can the reduce success rate if the annealing schedule is kept fixed. However, we show that adjusting the annealing schedule can compensate for this flattening effect, and allow for recovering a near-equivalent success rate as in the absence of V_{read} noise.

We now make this point explicit in the Discussion: there is no intrinsic algorithmic limitation that restricts our hybrid memristor-SMTJ Ising machine to sparse graphs. Sparsity mainly reduces programming effort and dynamic power, while denser graphs require appropriately dimensioned readout circuitry and, in practice, a slightly adapted annealing schedule.

• Please specify how the graphs are generated. Are they drawn from a random ensemble, or are they structured instances?

The graphs used in the experimental demonstrations were generated randomly. We first defined a weight granularity (three non-zero levels) and a target density, the latter limited mainly by the programming time discussed in other responses. Graphs were then drawn from a random ensemble under these constraints, following a procedure similar to that implemented in the `rudy` executable of the BiqMac library.

For the simulation-based scaling study, which we now added in Supplementary Note 1, we used the `rudy` tool itself to generate random weighted MAX-CUT instances of varying size, density, and number of levels.

We also added a short section on the graph generation procedure in the Methods of the revised manuscript, detailing the parameters used for both the MAX-CUT and graph-coloring instances.

2. Single vs. Multiple Graph Instances: The paper reports results for what appears to be a single instance of each graph problem. Why were multiple graphs not used for each problem type? Providing average performance across an ensemble would better validate the robustness and generality of the method.

The experimental demonstrations were performed on single representative instances of each problem type, since full programming and readout are time-intensive on our prototype. The goal was to show complete and functional hardware implementations of MAX-CUT and graph coloring, rather than to perform statistical averaging across many instances. To assess robustness more broadly, we have now carried out ensemble studies in simulation (new Supplementary Section 1: Variability and Scalability, Supplementary Figs. 1-4), featuring multiple random weighted MAX-CUT problems. In these simulations, we used a single annealing schedule.

Minor Questions

3. Spin Storage Architecture: In the hardware implementation (Fig. 1a), a single sMTJ is used to emulate spin-flip dynamics. Where are the values of all other spins stored? The "Spins vector" is referenced, but is this a separate digital memory component or an array of sMTJs?

We thank the reviewer for this question. In the present hardware prototype of Fig. 1a, only one physical SMTJ is used, and the full spin configuration is stored as digital bits in the control electronics, which we now clarify in the Methods. At each update, the selected spin value is converted into the corresponding input of the memristor crossbar, and the updated value is written back to this digital "Spins vector". In the projected integrated architecture (discussed in the revised Discussion and Supplementary Note 6), this digital storage would be replaced by an array of SMTJ p-bits, with one SMTJ per spin, associated with registers. This is discussed in the new Supplementary Note 6.

4. Programmability of Couplings J_{ij}: Can the crossbar architecture support both positive and negative couplings J_{ij}? In the MAX-CUT problem, all couplings are negative. In the coloring experiment, the couplings from each node are either all positive or all negative—a rather special case. Is it possible to realize arbitrary signed J_{ij} in this hardware, and if not, what modifications would be required to support this functionality?

We have included a dedicated subsection within the new Suppl. Note 6 addressing these questions. In the present prototype, each coupling is stored in a single memristor, and its sign is imposed at the column level during the MAC operation by selecting the polarity of V_{read}. A natural extension for future chips is to use a differential pair (g^+, g^-) per coupling, so that the effective weight is given by their difference

$$J_{ij} \propto g_{ij}^+ - g_{ij}^-$$

This scheme provides full per-weight sign control and improves robustness to slow drifts that are common to both devices (e.g., temperature-dependent shifts), as these are largely rejected in the differential operation.

The spin variables must be supplied dynamically, since they are updated at every sampling step. Several encoding options are available:

- *Ising encoding $s_j \in \{\pm 1\}$ with signed column voltages.* Each column driver directly applies $V_j = s_j V_{\text{read}}$, selecting between $+V_{\text{read}}$ and $-V_{\text{read}}$. The weight sign is then set by (g^+, g^-), and no additional per-cell routing is required.
- *Ising encoding with local routing.* All column voltages remain positive, and a small local switch routes either g^+ or g^- to the row summation line depending on s_j. This keeps the column drivers single-ended but introduces per-cell routing overhead.
- *QUBO encoding $x_j \in \{0, 1\}$.* Spins are encoded as x_j, and the problem is mapped via $s_j = 2x_j - 1$. Columns are either driven at V_{read} (for $x_j = 1$) or disconnected/held at reference (for $x_j = 0$), while the sign of the coupling is entirely carried by the differential pair (g^+, g^-).

All three options are compatible with the MAC operation; the choice depends primarily on circuit complexity, routing constraints, and whether the algorithm is formulated directly in Ising form or in QUBO form.

Summary This is an interesting paper demonstrating a proof-of-concept for probabilistic computing using spintronic devices. The experimental system is well designed, and the benchmarks show promising results. I recommend publication after the authors address the above points, particularly those regarding the complexity and generality of the benchmark graphs and the programmability of the hardware.

References

1. Singh, N. S., Kobayashi, K., Cao, Q., Selcuk, K., Hu, T., Niazi, S., Aadit, N. A., Kanai, S., Ohno, H., Fukami, S. *et al.* Cmos plus stochastic nanomagnets enabling heterogeneous computers for probabilistic inference and learning. *Nat. Commun.* **15**, 2685 (2024).
2. Aadit, N. A., Grimaldi, A., Carpentieri, M., Theogarajan, L., Martinis, J. M., Finocchio, G. & Camsari, K. Y. Massively parallel probabilistic computing with sparse ising machines. *Nat. Electron.* **5**, 460–468 (2022).
3. Nikhar, S., Kannan, S., Aadit, N. A., Chowdhury, S. & Camsari, K. Y. All-to-all reconfigurability with sparse and higher-order ising machines. *Nat. Commun.* **15**, 8977 (2024).
4. Isakov, S., Zintchenko, I., Rønnow, T. & Troyer, M. Optimised simulated annealing for ising spin glasses. *Comput. Phys. Commun.* **192**, 265–271 (2015).
5. Lee, K., Chowdhury, S. & Camsari, K. Y. Noise-augmented chaotic ising machines for combinatorial optimization and sampling. *Commun. Phys.* **8**, 35 (2025).
6. Borders, W. A., Pervaiz, A. Z., Fukami, S., Camsari, K. Y., Ohno, H. & Datta, S. Integer factorization using stochastic magnetic tunnel junctions. *Nature* **573**, 390–393 (2019).
7. Aadit, N. A., Grimaldi, A., Finocchio, G. & Camsari, K. Y. Physics-inspired ising computing with ring oscillator activated p-bits. In *2022 IEEE 22nd International Conference on Nanotechnology (NANO)*, 393–396 (2022).
8. Si, J., Yang, S., Cen, Y., Chen, J., Huang, Y., Yao, Z., Kim, D.-J., Cai, K., Yoo, J., Fong, X. *et al.* Energy-efficient superparamagnetic ising machine and its application to traveling salesman problems. *Nat. Commun.* **15**, 3457 (2024).
9. Kaiser, J., Borders, W. A., Camsari, K. Y., Fukami, S., Ohno, H. & Datta, S. Hardware-aware in situ learning based on stochastic magnetic tunnel junctions. *Phys. Rev. Appl.* **17**, 014016 (2022).
10. Yin, J. *et al.* Scalable ising computer based on ultra-fast field-free spin orbit torque stochastic device with extreme 1-bit quantization. In *2022 International Electron Devices Meeting (IEDM)*, 36.1.1–36.1.4 (IEEE, 2022).
11. Cai, F., Kumar, S., Van Vaerenbergh, T., Sheng, X., Liu, R., Li, C., Liu, Z., Foltin, M., Yu, S., Xia, Q., Yang, J. J., Beausoleil, R., Lu, W. D. & Strachan, J. P. Power-efficient combinatorial optimization using intrinsic noise in memristor hopfield neural networks. *Nat. Electron.* **3**, 409–418 (2020).
12. Jiang, M., Shan, K., He, C. & Li, C. Efficient combinatorial optimization by quantum-inspired parallel annealing in analogue memristor crossbar. *Nat. communications* **14**, 5927 (2023).
13. Kim, K., Youn, S., Park, J. & Kim, H. Ising solver using weight profile of memristor crossbar array for combinatorial optimization. In *2024 IEEE International Electron Devices Meeting (IEDM)*, 1–4 (IEEE, 2024).
14. Shan, K., Jiang, M., Du, Z., Xiao, Y., Tong, Y., Li, Z., Yang, S.-H., He, C., Mao, R., Lin, P. *et al.* One-step combinatorial optimization solver with fully integrated analog memristors and annealing module. In *2024 IEEE International Electron Devices Meeting (IEDM)*, 1–4 (IEEE, 2024).
15. Yin, X., Qian, Y., Vardar, A., Günther, M., Müller, F., Laleni, N., Zhao, Z., Jiang, Z., Shi, Z., Shi, Y. *et al.* Ferroelectric compute-in-memory annealer for combinatorial optimization problems. *Nat. Commun.* **15**, 2419 (2024).

Response to reviewers

Reviewer 1

I thank the authors for their detailed response and the significant effort put into revising the manuscript. The addition of the analog coupling experiment, the characterization of signal-to-noise ratio versus read voltage, and the variability simulations in Supplementary Note 1 have substantially strengthened the proposed device concepts and the overall quality of the work.

We thank the Reviewer for carefully reading the revised manuscript and for the constructive comments. We appreciate the positive feedback on the additional experiments and analyses, and we address the remaining points below.

1. While Figure 2 successfully demonstrates that the read voltage can modulate the sMTJ switching probability via an operational amplifier, this experiment appears to be a characterization rather than a functional demonstration of the system solving a problem. Based on the authors' response, the actual Max-Cut and graph coloring demonstrations still relied on a computer-in-the-loop configuration where a Python script determined the feedback voltage. Consequently, the claim that the solver operates with intrinsic analog annealing has not been fully experimentally realized for an optimization task. The manuscript should be revised to explicitly clarify that the problem-solving results rely on software-mediated feedback and that the purely hardware-based analog loop has so far been limited.

We thank the Reviewer for this important point. To remove any ambiguity, we have revised the manuscript in multiple locations to state that the MAX-CUT and graph coloring results (Figs. 4-5) were obtained in a computer-in-the-loop configuration. Concretely:

- The caption of Fig. 2 says explicitly that it uses the analog coupling setup presented in Suppl. Fig. 5, and the captions of Figs. 4-5 say explicitly that they use the computer-in-the-loop coupling setup presented in Suppl. Fig. 6
- In the Abstract, we now specify that the MAX-CUT and graph coloring optimization results are obtained using an *"externally implemented feedback loop"*.
- In the section "Solving a weighted MAX-CUT instance", we now describe the principle of computer-mediated coupling used in Figs. 4–5: *"Note that in this experiment, to facilitate data analysis, the coupling between SMTJ and memristor is now computer-mediated: the local field is computed by the memristor array, read out by a computer/controller, and the corresponding feedback/read voltage is then applied to the SMTJ (see Methods and Suppl. Fig. 6.)."* (In the previous version of the manuscript, this explanation was only in the Methods section.)

2. Furthermore, there are remaining concerns regarding scalability that the current simulations do not fully address. More effects need to be considered when the problem is scaled for a fully analog system, such as systematic IR drop in a crossbar array that alters effective coupling values and interconnect parasitics like RC delay, which can introduce synchronization issues. Additionally, the simulations are currently limited to

100-node instances, which are not computationally too difficult for digital computers. Since these results are generated via simulation and are not constrained by fabrication limits, the authors should evaluate significantly larger instances (e.g., 800 to 2000 nodes) to verify that the analog dynamics and signal margins remain robust at a scale where this technology would offer a genuine advantage.

IR drop and interconnect parasitics. We thank the Reviewer for raising this important question regarding scalability and crossbar/interconnect nonidealities. We now discuss these questions explictly in the Discussion of the revised manuscript: *"Scalability is also bounded by the practical size of analog in-memory computing tiles: interconnect resistance and capacitance cause IR drops and RC delays that distort the effective couplings and slow the analog settling dynamics. These limitations are well known in in-memory neural-network accelerators[1]. A standard mitigation is to avoid very large crossbars and instead tile the coupling matrix into moderate-sized arrays, then synthesize larger MAC operations by aggregating per-tile partial sums, optionally with per-tile calibration to compensate systematic attenuation and offsets[2]. Such composition can be realized either with reconfigurable, fully analog interconnect fabrics that avoid ADCs[3] or with hybrid analog/digital schemes that digitize at tile boundaries[4]. These approaches are highly effective: in-memory-computing neural networks with millions of weights can approach software accuracy[3–5]. For the present Ising machine, the ADC-less approach of Ref.[3] is particularly appealing because the analog sums can directly bias SMTJ p-bits, preserving end-to-end array-to-spin analog coupling while keeping annealing under global control."*

Larger-scale instance. To further explore the behavior of the proposed dynamics at larger scale within these constraints, we extended the numerical study to an ≈800-spin instance: a 77-vertex real-world network (Les Misérables) formulated as a 10-coloring problem, which expands via one-hot encoding to 770 spins. The results of this study are presented in a new Supplementary Note 8, where we report solver performance and robustness as a function of conductance variability, which directly impacts effective coupling margins.

At this scale, the large number of nominally zero couplings in the sparse interaction matrix leads to an accumulation of small residual conductance offsets, which can become non-negligible when summed over many connections. In Supplementary Note 8, we discuss this effect and show that the differential two-submatrix mapping introduced in Supplementary Note 6 enables accurate centering of near-zero weights, thereby preserving high success rates (>90%) for experimentally achievable variability levels.

These results illustrate how scale-dependent non-idealities emerge as problem size increases and how appropriate architectural choices can mitigate their impact on optimization performance. While we feel that a full parasitic-aware co-simulation of a large-scale integrated system is beyond the scope of the present work, the additional simulations reported here provide layout-agnostic evidence for the robustness of the proposed stochastic dynamics and intrinsic annealing mechanism at substantially larger scales.

Reviewer 2

The authors have adequately addressed the majority of the comments by providing detailed clarifications, additional figures in the main text, and expanded experimental explanations in the supplementary material. While large-scale hardware integration beyond simulation remains as future work, the manuscript clearly demonstrates a functional prototype leveraging SMTJ and RRAM devices for Ising machines. This work represents a meaningful contribution to the field. I therefore recommend this work for publication.

We thank the Reviewer for their careful evaluation of the revised manuscript and for the positive assessment of the work.

References

1. Hu, M., Strachan, J. P. & Li, C. Memristor-based analog computation and neural network acceleration. *Nat. Electron.* **1**, 324–332 (2018).

2. Xue, C.-X., Chiu, Y.-C., Liu, T.-W., Huang, T.-Y., Liu, J.-S., Chang, T.-W., Kao, H.-Y., Wang, J.-H., Wei, S.-Y., Lee, C.-Y. *et al.* A cmos-integrated compute-in-memory macro based on resistive random-access memory for ai edge devices. *Nat. Electron.* **4**, 81–90 (2021).

3. Ambrogio, S., Narayanan, P., Okazaki, A., Fasoli, A., Mackin, C., Hosokawa, K., Nomura, A., Yasuda, T., Chen, A., Friz, A. *et al.* An analog-ai chip for energy-efficient speech recognition and transcription. *Nature* **620**, 768–775 (2023).

4. Le Gallo, M., Khaddam-Aljameh, R., Stanisavljevic, M., Vasilopoulos, A., Kersting, B., Dazzi, M., Karunaratne, G., Brändli, M., Singh, A., Mueller, S. M. *et al.* A 64-core mixed-signal in-memory compute chip based on phase-change memory for deep neural network inference. *Nat. Electron.* **6**, 680–693 (2023).

5. Khwa, W.-S., Wen, T.-H., Hsu, H.-H., Huang, W.-H., Chang, Y.-C., Chiu, T.-C., Ke, Z.-E., Chin, Y.-H., Wen, H.-J., Hsu, W.-T. *et al.* A mixed-precision memristor and sram compute-in-memory ai processor. *Nature* **639**, 617–623 (2025).
